# Introducing an Optimization- and explicit Runge-Kutta- based Approach to Perform Dynamic Flux Balance Analysis

**DOI:** 10.1038/s41598-020-65457-4

**Published:** 2020-06-08

**Authors:** Wheaton L. Schroeder, Rajib Saha

**Affiliations:** Department of Chemical and Biomolecular Engineering, University of Nebraska - Lincoln, Lincoln, USA

**Keywords:** Computer modelling, Plant sciences, Computational models, Time series

## Abstract

In this work we introduce the generalized **O**ptimization- and explicit **R**unge-**K**utta-based **A**pproach (**ORKA**) to perform **d**ynamic **F**lux **B**alance **A**nalysis (**dFBA**), which is numerically more accurate and computationally tractable than existing approaches. ORKA is applied to a four-tissue (leaf, root, seed, and stem) model of *Arabidopsis thaliana*, p-ath773, uniquely capturing the core-metabolism of several stages of growth from seedling to senescence at hourly intervals. Model p-ath773 has been designed to show broad agreement with published plant-scale properties such as mass, maintenance, and senescence, yet leaving reaction-level behavior unconstrainted. Hence, it serves as a framework to study the reaction-level behavior necessary for observed plant-scale behavior. Two such case studies of reaction-level behavior include the lifecycle progression of sulfur metabolism and the diurnal flow of water throughout the plant. Specifically, p-ath773 shows how transpiration drives water flow through the plant and how water produced by leaf tissue metabolism may contribute significantly to transpired water. Investigation of sulfur metabolism elucidates frequent cross-compartment exchange of a standing pool of amino acids which is used to regulate the proton flow. Overall, p-ath773 and ORKA serve as scaffolds for dFBA-based lifecycle modeling of plants and other systems to further broaden the scope of *in silico* metabolic investigation.

## Introduction

The use of synthetic biology for the engineering of uni- and multi-cellular organisms to enhance desirable phenotypes in microbe, plant, and animal systems, has been well established and has been capable of affecting the lives of millions of individuals, such as in the case of artemisinin production in yeast or enhancing nutritional value of agricultural products^1,2^. Synthetic biology techniques have been applied to many plant systems such as tomatoes^3^, rice^4^, and maize^3^ to produce enhanced phenotypes often with application to human nutrition^2^, pest resistance^5^, and resilience to abiotic stresses^6^. Many of these efforts have focused on a genetic understanding and manipulation of the plant system (or plant tissue) in question, having relied on intuitive interventions such as changes in regulation, insertion of new gene(s), and deletion of gene(s) from competing pathway(s)^2,5,6^.

**Figure 1:**
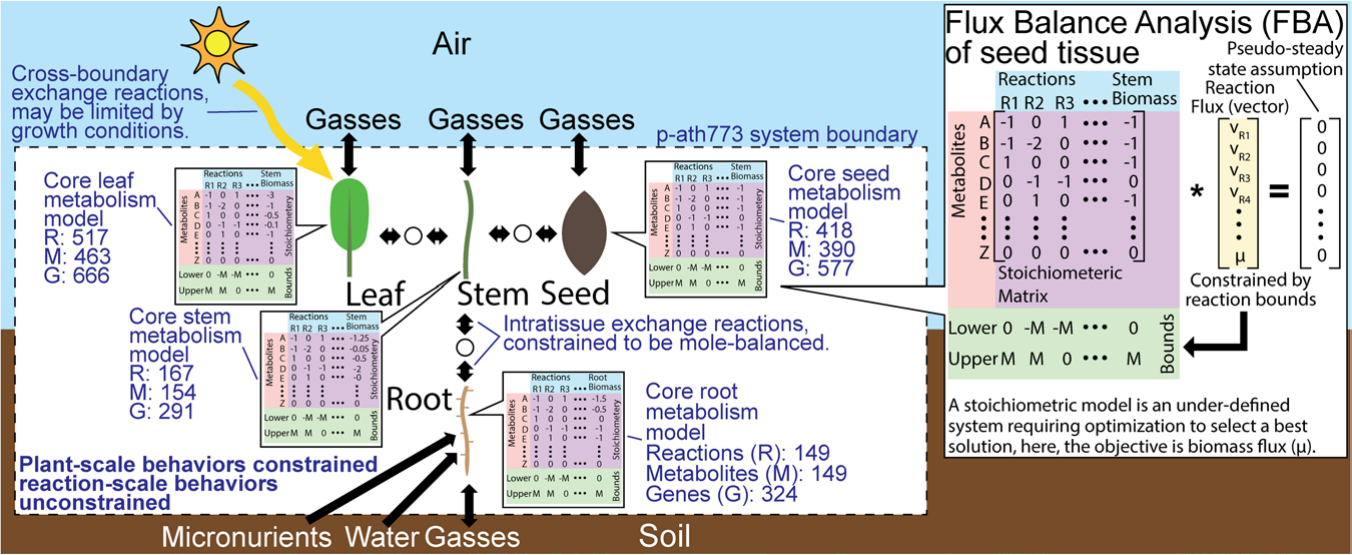
The p-ath773 system model. This figure emphasizes the individual nature of each of the four core tissue models (leaf, root, seed, and stem), formally defines the modeled system boundary (dashed black line), defines cross-boundary exchange reactions, intra-tissue exchange reactions, and gives the generic formulation for Flux Balance Analysis (FBA) applied to the seed tissue model.

Alternatively, computation-based systems biology approaches such as **S**toichiometric **M**odels (abbreviated as **SM**s; a list of all abbreviations used can be found in the “abbreviations used” section in Text S1, though all abbreviations are still defined at first use) of metabolism have provided a more rigorous method of metabolic investigation^7,8^. The set of reactions in an SM is defined by the **G**ene-**P**rotein-**R**eaction (**GPR**) links^8,9^ in an organism^7^. When an SM encompasses the entire chemical reaction repertoire of an organism, it is called a **G**enome **S**cale **M**odels (abbreviated as **GSM** or GEM)^10^. Perhaps the most common analysis tool used with these models is **F**lux **B**alance **A**nalysis (**FBA**)^8,11^. Assuming that the system is under pseudo-steady state, FBA can find the extremum of a given growth objective (often minimizing uptake of some nutrient^12,13^, maximizing growth^8^, or maximizing a desired bioproduct^9^) which is defined by an objective function subject to mass balance, reaction directionality, and certain other constraints (generally restricting growth rate or nutrient uptake depending upon the objective function)^7–9,13^. The basic formulation of FBA can be found in Fig. 1. In addition, tools which expand on the functionality of the basic FBA formulation, such as **d**ynamic **FBA** (**dFBA**)^14^ can improve the predictive abilities of SMs. dFBA can perform FBA over windows of time by solving a dynamic non-linear or a static linear problem, both of which integrate system variables over discrete time windows to solve for metabolite concentrations, reaction fluxes, and system biomass^14,15^. In general, there are two approaches to dFBA. The first approach, the **S**tatic **O**ptimization-based **A**pproach (**SOA**), has been applied to *E. coli*^14^, mammalian cells^16,17^, *Saccharomyces cerevisiae* (bakers’ yeast)^17^, *Hordeum vulgare* (barley)^15,18^, and *Arabidopsis thaliana*^19^ (in addition to other systems). The second, the **D**ynamic **O**ptimization-based **A**pproach (DOA), has been applied to *E. coli* metabolism^14^ and signaling networks is *S. cerevisiae*^20^ (to name a few applications). These approaches have proven useful for investigating aspects of plant-scale metabolism, such as resource partitioning in *Arabidopsis*^19^. These works have inspired the development of our new approach to perform dFBA named as **O**ptimization- and explicit **R**unge-**K**utta –based **A**pproach (**ORKA**). ORKA significantly improves upon the SOA by utilizing the step-by-step solution approach of the SOA (as opposed to simultaneous solution of all times in the DOA) with increased accuracy and solution stability. These improved characteristics are due to both the implementation of a Runge-Kutta method (a multi-step numerical method for the solution of ordinary differential equations) to replace the first-order Taylor series approximation used by SOA and by replacing the assumption that the reaction rate is constant over each time interval with a trapezoid rule-based integral approximation.

*Arabidopsis thaliana* (hereafter *Arabidopsis*) has been selected as a test system for the application and demonstration of the ORKA framework, due to the fact that *Arabidopsis* is a model plant species with a highly characterized knowledgebase. The choice has also allowed demonstration of ORKA in a dynamic, multi-tissue system. To date, many stoichiometric models of plant metabolism, including *Arabidopsis*, have been developed. Some of these models including models of *Arabidopsis thaliana*^13,15,21,22^, *Zea mayz* (maize)^23^, *Sorghum bicolor* (sorghum)^12^, *Brassica napus* (rapeseed)^24^, and *Oryza sativa* (rice)^25^ have treated plants as single metabolic units. These models have sought to analyze metabolic maintenance, response to abiotic stimuli, enzyme regulation changes, and metabolism as a whole^12,13,15,21–25^. Tissue-specific models have been reconstructed for various *Arabidopsis* tissues^26^, a maize leaf^27^, and a barley seed^28^ to better understand how present metabolites, metabolic pathways, and nutrient (generally carbon and nitrogen) availability differ between tissues. Multi-tissue models have also been developed to characterize whole-plant metabolism for *Arabidopsis*^13,19^ and barley^18^ and subsequently to study whole-plant metabolic response to the diurnal cycle and the source-to-sink relationship of leaves and seeds^13,18^. These studies have considered metabolism at a single point (often in the exponential growth phase^12,18,21–23,25^) or a single diurnal cycle^13^ or have modeled only a portion of the *Arabidopsis* lifecycle^19^. The most complete dFBA work, in terms of modeling the full *Arabidopsis* lifecycle, models two tissues, leaf and root, across 30 days of vegetative growth (from 6 days to 36 days)^19^. Here, we have developed a core-carbon metabolic model of *Arabidopsis*, named **p-ath773** (**p**lant-scale core-metabolism *Arabidopsis thaliana* model with **773** genes included), to model the full lifecycle of *Arabidopsis* from germination to senescence by being embedded in the ORKA framework which captures metabolic interactions between four major tissues: leaf, root, seed, and stem. These four tissues have been chosen for model reconstruction to represent core plant functions: the root for nutrient uptake and growth; the leaf for photosynthesis, carbon fixation, and as a source tissue for plant nutrition; the seed for metabolite storage and a sink tissue for metabolic investment; and the stem for metabolic transport and acting as a conduit for all metabolic interactions between other tissues. Core-metabolism pathways that are included but not limited to photosynthesis; the citrate cycle; starch and sucrose synthesis; fatty acid synthesis and degradation; and amino acid synthesis. The p-ath773 model consists of 1251 total (and 631 unique, defined as having the same identifier across any number of subcellular compartments) reactions (R), 1155 total (and 276 unique) metabolites (M), and accounts for 773 genes (G) including 42 chloroplastic and 11 mitochondrial genes. Each of the modelled tissues including leaf (R: 517, M: 463, and G: 666), root (R: 149, M: 149, and G: 324), seed (R: 418, M: 390, and G: 577), and stem (R: 167, M: 154, and G: 291) has been reconstructed individually to allow for the different tissue mass ratios found across the lifecycle of the plant. A summary of the p-ath773 model is shown in Fig. 1. The ORKA framework determines biomass, metabolite concentrations, reaction flux, change in biomass, and changes in metabolic concentration (collectively defined as a metabolic “snapshot”) hourly across the lifecycle of *Arabidopsis* as modeled by p-ath773 under 12 hour light and 12 hour darkness growth conditions accounting for changes due to diurnal metabolic differences; changes in plant mass; metabolite storage and uptake (particularly carbohydrates); changes in plant tissue mass ratios; and changes in metabolism with respect to plant growth stage. The p-ath773 model is unique among *Arabidopsis* models for its focus on plant-scale behavior such as focus on achieving biomass levels which correspond with *in vivo* data; biomass-based maintenance and senescence drains; and the logical mole-balanced exchange of nutrients between tissues. While the plant-scale behavior is well-constrained in the p-ath773 model, reaction-scale behavior is unconstrained such that the model can be used to study the reaction-scale behavior necessary to explain observed macro-scale behavior.

When ORKA has been applied to the p-ath773 multi-tissue model, the order of error of both mass step and metabolite concentration estimates has been theoretically improved by approximately three order of magnitude as compared to that achieved in a previous model of *Arabidopsis* which utilized the SOA to perform dFBA^19^. This has been done by combining improved mass step and metabolite concentration estimates with smaller time step sizes (one hours as opposed to one day^19^). Further, with the inclusion of two more tissue types, stem and seed, and modeling the entire lifecycle, the p-ath773 model in the ORKA framework makes a significant improvement to current *Arabodipsis* dFBA-based models, despite only modelling central metabolism. It should be noted that for metabolic models with a single tissue, or single organism, O(*h*^3^) or better error order is possible depending on the Runge-Kutta method selected, compared to the O(*h*^2^) error order floor of the SOA method. This low error level has proved impossible to achieve with the p-ath773 model since the seed tissue appears and disappears over the course of the *Arabidopsis* lifecycle, causing difficulties due to the exponential nature of FBA-determined growth rates. The series of more accurate hourly metabolic “snapshots” produced by p-ath773 has given a framework for the investigation of the central metabolism of *Arabidopsis* across its lifecycle. Here, these “snapshots” have been used to investigate the diurnal patterns of water flow (from the root uptake to transpiration from the leaf), and sulfur metabolism (from root uptake to tissue biomass). Further, the p-ath773 model embedded in the ORKA framework has shown general agreement with macro-level experimental data found in the literature and is potentially useful as steppingstone for dynamic lifecycle modeling of other plant systems.

## Results

### Development of the ORKA to perform dFBA

**Figure 2:**
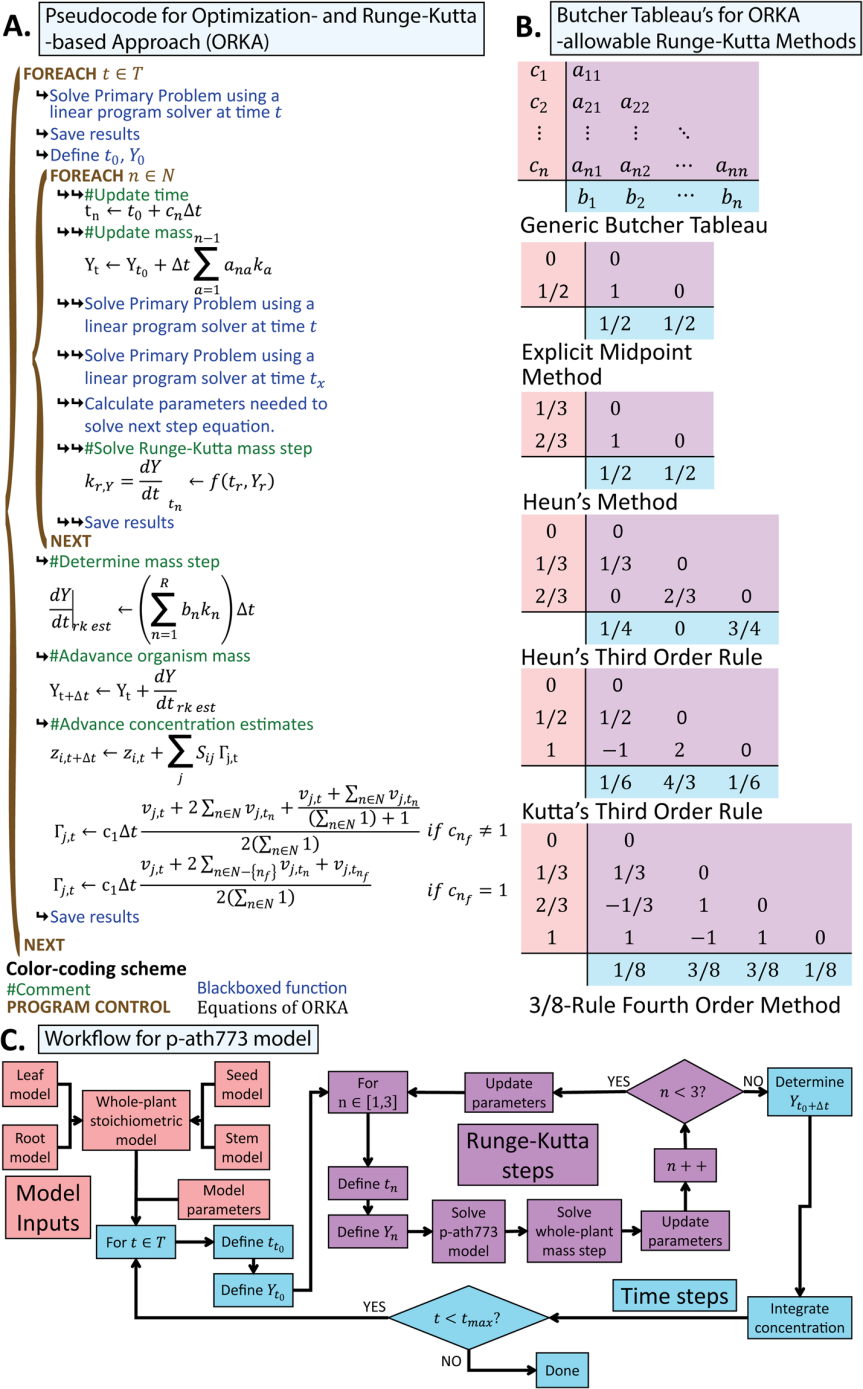
Pseudocode, acceptable Runge-Kutta methods, and workflow with p-ath773 related to ORKA. This figure shows simple pseudocode appropriate to the implementation of the generic ORKA method in (**A**), Runge-Kutta method appropriate for use with the ORKA method in (**B**), and the workflow used in the specific application of ORKA to the p-ath773 model in (**C**). Symbols are defined as follows: *t*_*n*_ is the current time, *t*_0_ is the initial time, Δ*t* is the time step, *c*_*n*_ are the steps in the independent variable made by the Runge-Kutta method chosen to use, *Y*_*t*_ is the current biomass concentration, *Y*_0_ is the initial biomass concentration, *a*_*na*_ is the weight of Runge-Kutta derivative estimate steps (*k*_*n*_) in the next derivative estimate, *b*_*n*_ is the weight of the Runge-Kutta derivative estimate steps in the full Runge-Kutta derivative estimate, ${\frac{dY}{dt}|}_{rkest}$ is the Runge-Kutta derivative estimate for the current timestep, *Y*_*t* + Δ*t*_ is the biomass concentration at the next time step, *z*_*i*,*t*_ is the concentration of metabolite *i* at time *t*, *z*_*i*,*t* + Δ*t*_ is the concentration of metabolite *i* at the next time step, *S*_*ij*_ is the stoichiometric coefficient of metabolite *i* in reaction *j*, Γ_*j*,*t*_ is the trapezoid rule-based integral estimate of the flux of reaction *j* at the current timestep, *v*_*j*,*t*_ is the rate of reaction *j* at time *t*, *v*_*j*,*tn*_ is the rate of reaction *j* at Runge-Kutta time step *n*, set *N* is the number of steps in the Runge-Kutta solution method (with *n* as the index), and *c*_*nf*_ is the final *c*_*n*_ value in the Runge-Kutta method used. Greater detail on the definition of each symbols used can be found in the “Symbols Used” section in Text S1. In (**A**), there are two control loops (brown text with brown left-handed braces), one looping over each time point in the set of times over which to apply ORKA (*t* ∈ *T*), and the other looping over each step in the selected Runge-Kutta method (*n* ∈ *N*). The former control loop is used to solve the model at the time point, define the starting points for the Runge-Kutta method, and, after the Runge-Kutta loop is finished, advance biomass and metabolite concentrations in the model. The inner control loop determines the values of the Runge-Kutta-based concentration and biomass step estimates. The various estimates used rely on evenly spaced points at which the estimates are made, limiting the selection of Runge-Kutta method. Some allowable Runge-Kutta methods are shown in (**B**). For this work, Heun’s Third Order Rule was selected. In (**C**), an overview of the workflow used to integrate the p-ath733 model (red) in the ORKA method (blue and purple) is shown.

The Optimization- and explicit Runge-Kutta- based Approach (ORKA) has been developed to make more accurate and stable estimates of the changes in biomass and metabolite concentration in a dynamic Flux Balance Analysis (dFBA). The basis of the ORKA is the same as SOA, to model a dynamic (i.e. time-dependent) metabolism across multiple time points, where each time point solution builds upon previous solutions. The pseudocode describing how the ORKA works can be found in Fig. 2A. Symbols are defined as follows: *t*_*n*_ is the current time, *t*_0_ is the initial time, Δ*t* is the time step, *c*_*n*_ are the steps in the independent variable made by the Runge-Kutta method chosen to use, *Y*_*t*_ is the current biomass concentration, *Y*_0_ is the initial biomass concentration, *a*_*na*_ is the weight of Runge-Kutta derivative estimate steps (*k*_*n*_) in the next derivative estimate, *b*_*n*_ is the weight of the Runge-Kutta derivative estimate steps in the full Runge-Kutta derivative estimate, ${\frac{dY}{dt}|}_{rkest}$ is the Runge-Kutta derivative estimate for the current timestep, *Y*_*t* + Δ*t*_ is the biomass concentration at the next time step, *z*_*i*,*t*_ is the concentration of metabolite *i* at time *t*, *z*_*i*,*t* + Δ*t*_ is the concentration of metabolite *i* at the next time step, *S*_*ij*_ is the stoichiometric coefficient of metabolite *i* in reaction *j*, Γ_*j*,*t*_ is the trapezoid rule-based integral estimate of the flux of reaction *j* at the current timestep, *v*_*j*,*t*_ is the rate of reaction *j* at time *t*, *v*_*j*,*tn*_ is the rate of reaction *j* at Runge-Kutta time step *n*, set *N* is the number of steps in the Runge-Kutta solution method (with *n* as the index), and *c*_*nf*_ is the final *c*_*n*_ value in the Runge-Kutta method. Greater detail on the definition of each symbols used can be found in the “Symbols Used” section in Text S1. ORKA expands upon the SOA approach^28^ by replacing the Taylor-series approximations (details in the methods section) used to advance biomass concentration in the SOA (Y_t_ in Fig. 2A) with a Runge-Kutta-based estimate for increased model accuracy and solution stability. The ORKA framework in this pseudocode formulation is left generic enough so that a variety of Runge-Kutta methods can be used, as long as *c*_*n*_ values are evenly spaced. Here examples of Runge-Kutta methods which such *c*_*n*_ values include those shown in Butcher Tableaus in Fig. 2. A detailed formulation of ORKA can be found in the Materials and Methods. A summary of the ORKA formulation is as follows. Begin with an SM; a set of time points over which to solve that SM; an initial condition related to the biomass of the system and metabolite concentrations; and a chosen Runge-Kutta method to use in the solution. For each time step, solve the SM using linear programming at the beginning of that time step and define the initial conditions (time, biomass, and metabolite concentrations). The chosen Runge-Kutta method is used to solve the change in those initial conditions over the time step. This is done by solving the SM using linear programming and saving all reaction rates for each solution for the given time step to determine the mass step estimate of the given Runge-Kutta step. Once all Runge-Kutta steps are complete, the final mass step estimate for the given time step is made. To advance metabolite concentration, the integral from the start of the time step to the end of the timestep is estimated using the multi-application Trapezoid rule, which in turn is used to estimate the change in metabolite concentrations. This is followed by applying that mass step and concentration change estimates and repeating the process for the next time step. This process is shown more technically by a pseudocode described in Fig. 2A and explained in full detail in Materials and Methods.

### Reconstruction of arabidopsis core metabolism in tissue-specific models

**Figure 3:**
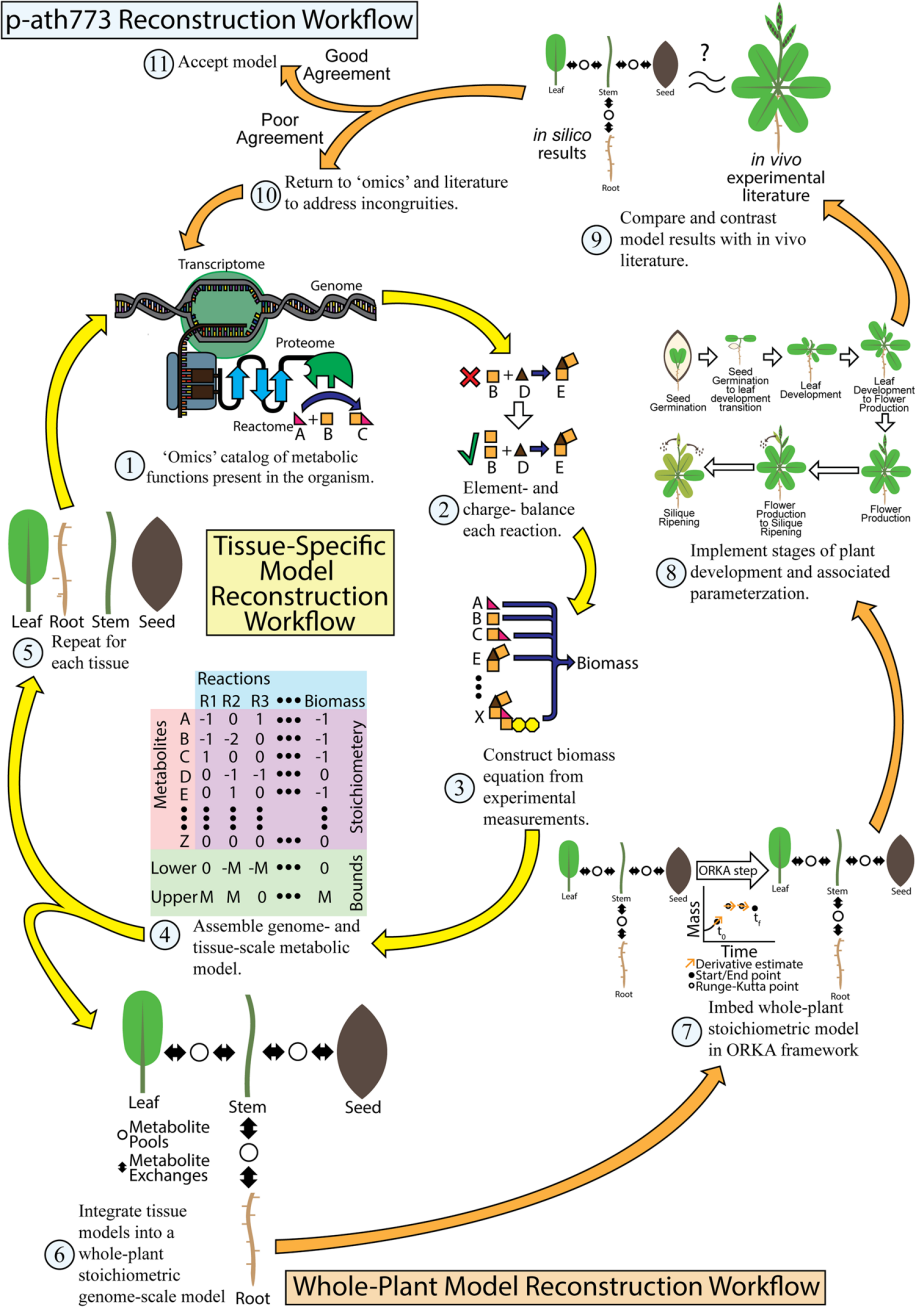
Workflow for p-ath773 model reconstruction. This figure shows the workflow used in the reconstruction and curation of individual tissue models (yellow arrows) and the integrated p-ath773 model as a whole (orange arrows). The reconstruction procedure begins by consulting published ‘omics’ data which helps identify which metabolic functions are present in a given tissue, followed by element- and charge- balancing the reactions representing those functions. A biomass equation is defined from literature evidence, and a stoichiometric model of the reconstruction is created. This is repeated for each tissue until a plant-scale model can be created. This model is then placed in the ORKA framework, and is used to simulate plant growth throughout its lifecycle. The results are compared with *in vivo* experimental results, such as those shown in Fig. 6. Incongruities are addressed at the tissue-level by re-consulting ‘omics’ level data. This process is repeated until an acceptable model is achieved.

**Figure 4:**
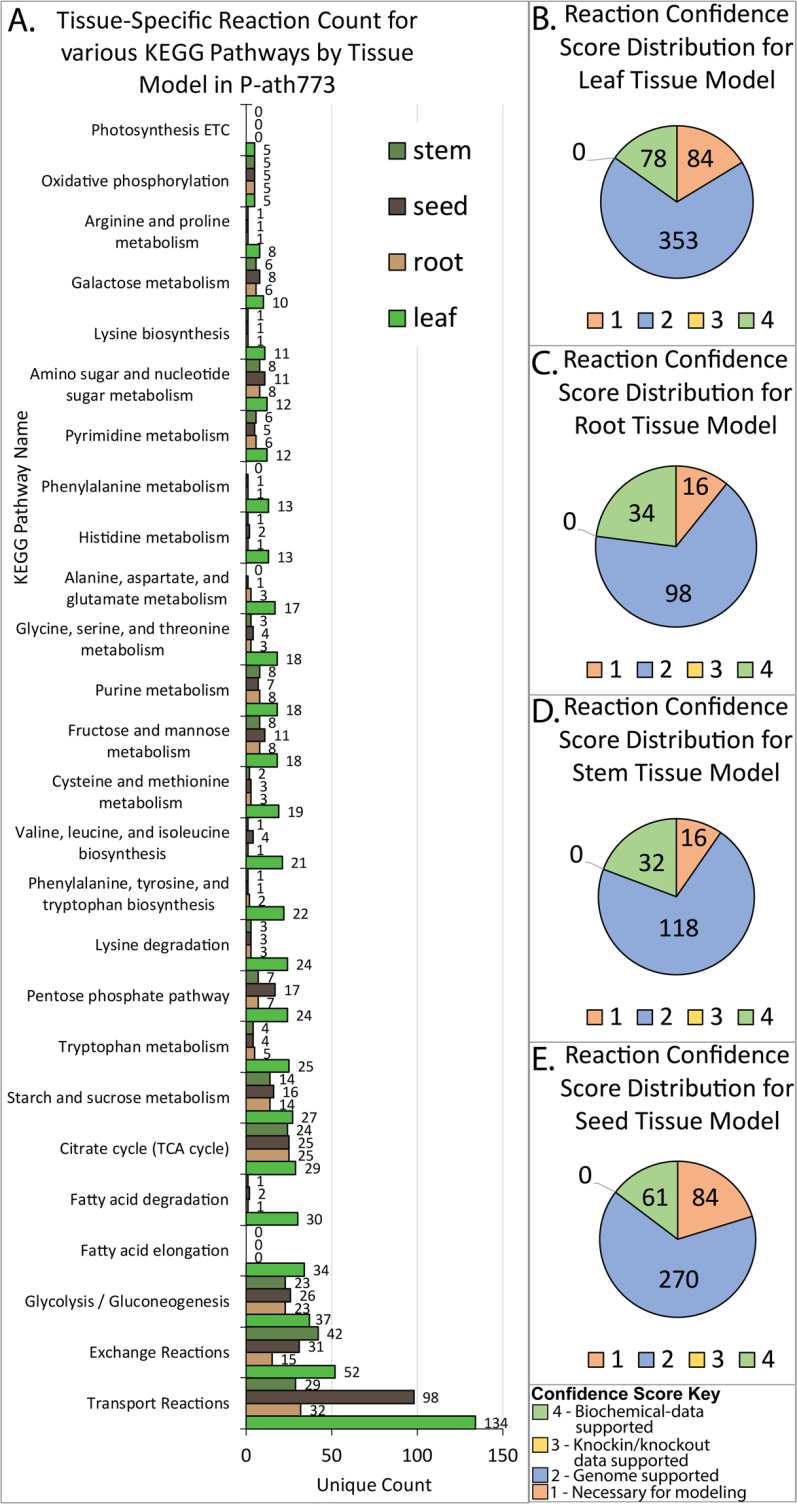
Statistics of tissue stoichiometric model reconstructions. Shown here are statistics related to the reconstruction of the leaf, root, seed, and stem models. (**A**) shows the types of reactions included in each of the four tissue models by counting the number of transport reactions, exchange reactions, and categorizing the remaining reactions based on the KEGG pathway(s) to which they belong. As shown here, the leaf model is the most complete and contains the most reactions is almost every category. Importantly, the leaf is the only tissue which contains reactions related to the photosynthetic electron transport chain (labeled “Photosynthesis ETC”). Figures (**B**) through (**E**) shows the rational for the inclusion of each reaction in each model using confidence scoring (see Thiele and Palsson for a definition and discussion of confidence scores). To summarize these figures, most reactions are included because there is evidence in the genome for these metabolic functions. The next most common reason for inclusion is being supported by biochemical literature data (e.g. a study has specifically identified the protein and determined its mechanism). The next most common reason for inclusion was modelling necessity (score of 1). No knockin/knockout studies where consulted in this work (score 3).

In order to track the important metabolic interactions and transactions within and between major tissues of *Arabidopsis* plant, namely seed, leaf, root, and stem, corresponding tissue-level metabolic models have been reconstructed. The seed and leaf tissue have been selected to model an important source-to-sink relationship, whereas the stem and root tissues have been included to model nutrient transport and nutrient uptake in *Arabidopsis*, respectively. Model files for each tissue can be found in the GitHub p-ath773 repository for this work (10.5281/zenodo.3735103). Details of model reconstruction can be found in Materials and Methods, but a synopsis is as follows. The seed model has been reconstructed first using the metabolic pathways shown in the *Arabidopsis* seed though ^13^C-labeled Metabolic Flux Analysis (MFA)^29^. The model reactions have been distributed among extracellular space, cytosol, non-green plastid, inner mitochondria, and outer mitochondria subcellular compartments in accordance with literature evidence (see list of works cited in Data S1). Next, transport and exchange reactions have been added to the model based on literature evidence (see list of works cited in Data S1) or to increase model connectivity^7^. The biomass composition of the seed has been determined from literature^29,30^. The resultant model is charge and element balanced, and has undergone multiple iterations of curation consistent with well-established GSM reconstruction protocols^7^. Once the seed model has been reconstructed, metabolic pathways common to both the seed and leaf tissue have been used as the starting point for reconstructing the leaf tissue model. To this model has been added additional amino acid syntheses (for xylem and phloem loading), photosynthesis, and gluconeogenesis as well as chloroplast and thylakoid subcellular compartments. The biomass of the leaf has been adapted slightly from that of a previously published *Arabidopsis* model, *i*RS1597^23^, by refocusing the biomass composition on primary metabolites. Similarly, by having extracted common reactions/pathways from the seed and leaf models as a starting point and adding functionalities particular to these tissues such as nitrogen reduction in the root and the transport of metabolites through the extracellular space of the stem, the root and stem models have been reconstructed. Root and stem models have been reconstructed with metabolic differences between the two such as the presence of amino acid synthesis and the conversion of ammonium to nitrate both in the root for xylem loading. Root and stem tissues are, however, largely focus on basic carbon metabolism and metabolite uptake (root) and transport (root and stem). In the absence of *Arabidopsis*-specific estimates, the dry weight compositions of switchgrass (*Panicum virgatum*) root and stem^31^ have been used to define root and stem biomass compositions. Both these models contain necessary transport/exchange reactions to ensure model connectivity and to facilitate their roles in the transport processes. The stem and root models have all the subcellular compartments present in the seed model. Once initial reconstructions have been accomplished, thermodynamically infeasible cycles in addition to atom and charge imbalances have been resolved^7^. Figure 3 shows the iterative process of model curation for tissue-specific model reconstructions used in this work (yellow arrow) and for the whole-plant iterative model curation (orange arrows). Figure 4 shows a summary of the distribution of model reactions across KEGG-defined pathways of each tissue model and an overview of reasons for reaction inclusion through confidence scoring (see Method section)^7^. Figure 4A summarizes the pathways common to all tissues and Figs. 4B through 4E graphically summarize the sources of reactions in each tissue model through confidence scores (see methods section)^7^. Once each tissue model has been reconstructed, these four models have been linked by the ORKA framework, and the lifecycle of the plant has been simulated. We have addressed the incongruities between these *in silico* simulation results and *in vivo* experimental data by adjusting their metabolism of individual tissue-specific models, tissue-tissue interactions, or by adjusting parameters (such as biomass yield, plant maintenance, and plant senescence) associated with the plant-scale behavior of the p-ath773 model. This portion of the workflow is illustrated in Fig. 3 (orange arrows).

### Development of constraints defining tissue-tissue interactions in the p-ath773 model

Once these core tissue models have been reconstructed and curated, sets of constraints have been defined to enforce logical links between tissues to facilitate the simulation of tissue metabolism. For instance, these links include ensuring that water travels from the root (source) to the leaves (sink) and that literature-supported amino acids travel from the leaf and root (sources) to the seed (sink) through the stem tissue (the link between these tissues). In addition, other constraints include environmental interactions such as with atmosphere and soil. These constraints include gas exchange in all tissues; uptake of micronutrients and water by the roots; and use of light by the leaves. These constraints are discussed in detail in the Materials and Methods. In summary, these constraints ensure that micronutrients and water are transported from the root tissue to other tissues via the stem; that sugars and amino acids travel from the leaf tissue to other tissues via the stem; that patterns of starch and sucrose storage in leaf and stem tissues are included in the model; and that the rates of tissue growth are linked in such a way that tissue mass ratios are preserved or changed in accordance with how these quantities change in an *Arabidopsis* plant as it passes through various stages of growth.

### Simulating stages of plant growth using p-ath773 and ORKA

**Figure 5:**
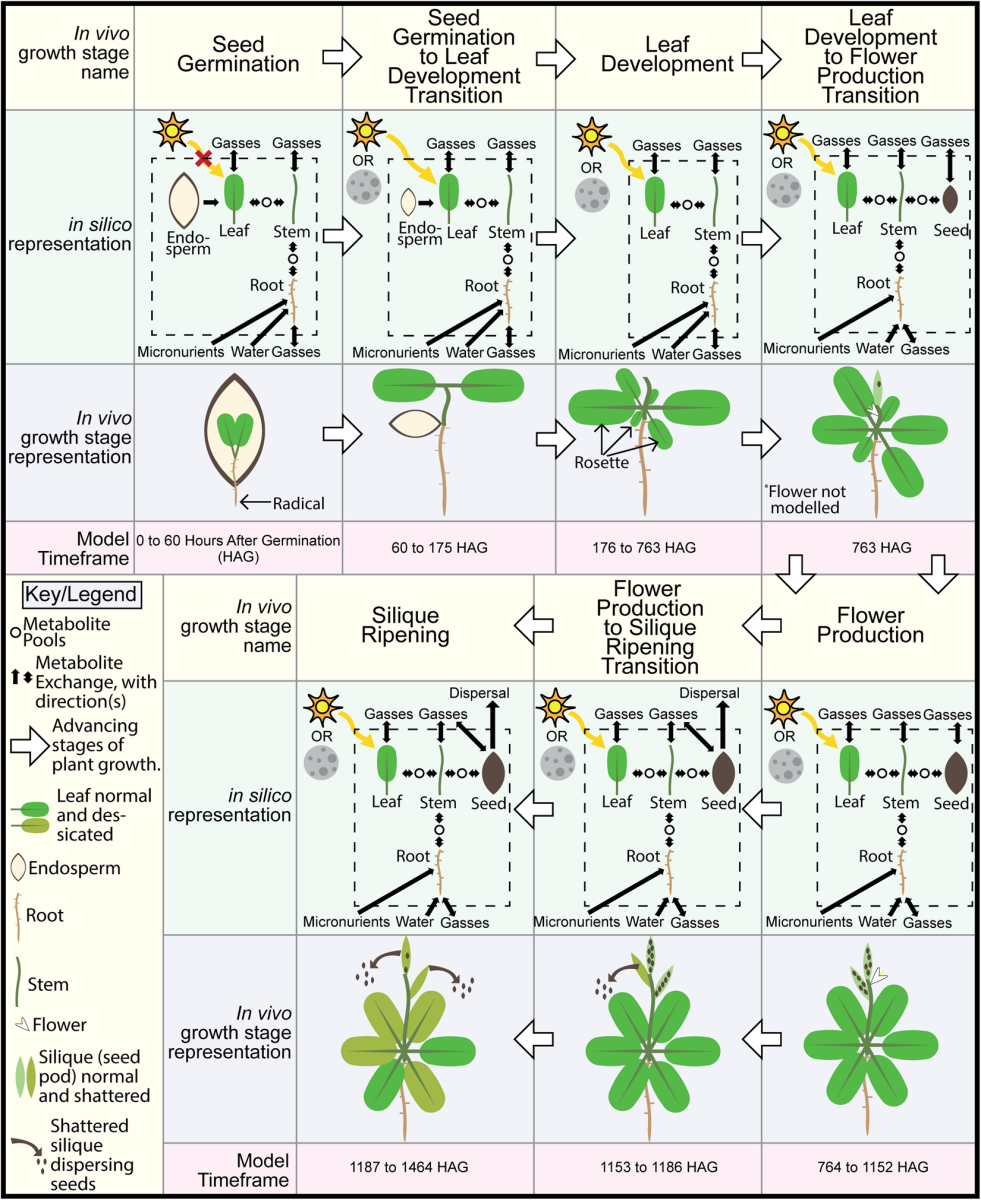
Seven growth stages in the p-ath773 model. Shown here are the labels given to each *in vivo* growth stage modeled *in silico* by p-ath773 (yellow headings), a sketch of the *in silico* representation (green rows) of the modeled plant system, a sketch of what the *in vivo* plant would look like at said growth stage (blue rows), and the timeframe in which the p-ath773 model simulates that growth stage as holding sway (red rows). White arrows indicate the progression of the system from germination to senescence. The *in silico* representation is a simplified drawing of what is occurring *in silico* showing major issue metabolite exchanges (black arrows), metabolite pools (open black circles) and interactions outside the system (black arrows crossing dashed-line box).

As discussed more extensively in Materials and Methods, the growth rate for an SM is an exponential growth rate. Due to this exponential nature of the growth rate, seed mass becomes problematic to model as there are points in the growth of the seed tissue where its mass is zero, is advanced from zero to a non-zero value, and is advanced from a non-zero value to zero. These changes are impossible to capture using an exponential function. Therefore, plant mass as a whole is tracked and advanced by the ORKA, and individual tissues masses are determined by multiplying total plant mass by tissue mass fraction. Since there is no whole-plant biomass function, this approach requires an approximation which defines the error floor by a second order backward difference approximation of the first derivative (see Materials and Methods for details) with an error order of *O*((*c*_2_ − *c*_1_)*h*^2^). Therefore, any Runge-Kutta method with error order less than that will suffice. In this work, Heun’s third-order Runge-Kutta rule is used. This is in part because of the limitation just described such that a higher-order Runge-Kutta method is not necessary. Further, this method has the advantage over Kutta’s third-order Runge-Kutta rule in that the step size between *c*_*n*_ values is one third (e.g. *c*_2_ − *c*_1_ = 1/3) as opposed to one half (e.g. *c*_2_ − *c*_1_ = 1/2) (see Fig. 2B), giving slightly lower error for trapezoid rule-based integration and backward difference approximation estimates. A simplified workflow of how p-ath773 is integrated into the ORKA framework is shown in Fig. 2C and a more detailed explanation is included in Materials and Methods. In summary, the p-ath773 model includes the four tissue models and tissue-tissue interactions, whereas the ORKA to perform dFBA is the approach used to simulate the model form one timepoint to the next. The simulations of the p-ath773 model has been advanced through several growth stages using time points for changes in growth stage taken from experimental data^32^, see Fig. 5. Fig. 5 highlights the timepoints spread out through the seven growth stages modeled here including seed germination, seed germination to leaf development transition, leaf development, leaf development to flower production transition, flower production, flower production to silique ripening transition, and silique ripening. Fig. 5 further provides sketches of the *in silico* and *in vivo* representations for each of these growth stages. In the seed germination stage, the uptake of fatty acids, sugars, and amino acids from seed storage (endosperm, see Seed Germination stage in Fig. 5) has been modeled as a rate of usage which results in all stored fatty and amino acids being depleted by the end of the seed germination to leaf development transition^30^. This rate has been determined such that it is constant in mmol/h (see Data S1) yet needed conversion to the mmol/gDW·h units used throughout the p-ath773 model. Therefore, the rate at which the endosperm is utilized is scaled by the gDW of the leaf tissue (as the leaf tissue is modeled as interacting directly with the endosperm). This scaling advantageously results in a gradual decrease of the rate of nutrients uptaken from the endosperm stores (in mmol/gDW·h), as would happen in a seedling as the plant mass begins to far exceed the mass of the endosperm. A 12:12 hour light:dark diurnal rhythm has been chosen to match experimental conditions for the studies on starch and sucrose storage/uptake dependence^33^. Diurnal metabolism affects the model at all growth stages except for Seed Germination, when the cotyledons (embryonic leaves) are shaded from light by the soil and/or seed coat. In growth stages when plant tissue ratios are constant (i.e., the vegetative stages such as Seed Germination through Leaf Development), the tissue mass ratio values have been taken from values typical for herbaceous plants (0.511 gDW leaf:0.0.267 gDW root:0.211 gDW stem after adjusting from fresh weight to dry weight)^33–35^ (See Data S1). In growth stages when the ratios between tissues change^32^ (i.e., seed production or dispersion stage), a linear biomass “slider” is used, where a single parameter, seeding (*s*), is used to progress tissue mass ratios (see Fig. 5). This ranges from *s* = 0 (normal vegetative tissue mass ratios) to *s* = 1 (mass ratios when maximum amount of seeds have been produced and have not yet been dispersed) and is linearly incremented from the point at which the first flower is produced to when all flowers are produced then decremented to when all silique (seed pods) are shattered, thus dispersing all seeds (see Data S1). A workflow showing how ORKA is applied to the p-ath773 model can be found in Fig. 2C. In addition to using ORKA to perform dFBA, Flux Variability Analysis (FVA) has been performed, at twelve points throughout the *Arabidopsis* lifecycle, selected to represent each growth stage and diurnal status in those stages (save the Leaf Development to Flower Production transitions which includes a single time point, see Fig. 5), subject to all growth constraints, and a growth rate equivalent to the optimal growth rate to evaluate the variability in the balanced flux estimates. Flux Variability Analysis is performed at 1 **H**our(s) **A**fter **G**ermination (**HAG**, seed germination stage, dark), 70 HAG (seed germination to leaf development transition, light), 90 HAG (seed germination to leaf development transition, dark), 177 HAG (leaf development stage, light), 181 HAG (leaf development stage, light), 770 HAG (flower production stage, light), 810 HAG (flower production stage, dark), 1155 HAG (flower production to silique ripening transition, light), 1170 HAG (flower production to silique ripening transition, dark), 1190 HAG (silique ripening stage, dark), and 1199 HAG (silique ripening stage, light). In summary, we incorporated the p-ath773 model in an ORKA framework to simulate *Arabidopsis* metabolism across the lifecycle of an individual plant.

### Design-build-test cycling of the p-ath773 model in the ORKA framework

**Figure 6:**
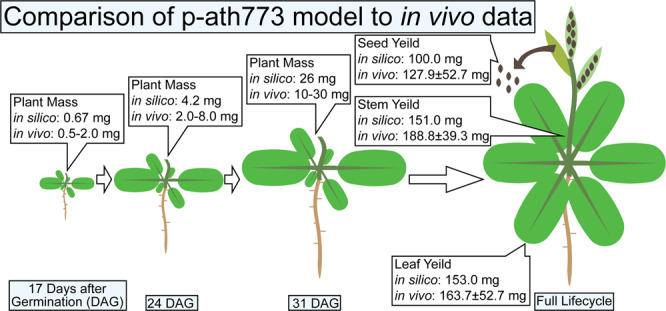
Comparison of plant-scale growth between *in vivo* data and the p-ath773 model. The figure shows some plant-scale growth check points which were used to verify the accuracy of the plant-scale growth pattern. The first three checkpoints were in the leaf development phase as 17 Days After Germination (DAG), 24 DAG, and 31 DAG, with *in vivo* experimental ranges for whole-plant mass and *in silico* whole-plant mass of the p-ath773 model shown in the callouts. The final image is for total tissue yield, where the reported *in silico* value is the maximum mass of each tissue during the entire lifecycle, and the *in vivo* value is the mean dry weight of the specified tissue at harvest plus or minus one standard deviation.

Once growth stages have been implemented with the p-ath773 model and the ORKA framework, the design-build-test cycle (shown in Fig. 3) has been used to iteratively improve and refine the p-ath773 model. The data points used to determine how well the model fits experimental literature include the mass of the whole plant at certain benchmark times and peak mass yields of leaf, seed, and stem tissues^32,34^. At 17, 24, and 31 Days After Germination (DAG) the total dry plant mass should be between 0.5 and 2.0 mg; 2 and 8 mg; and 10 and 30 mg, respectively^34^. Upon the completion of multiple iteration of design-build-test cycle, the p-ath773 model has been adequately refined, the p-ath773 model has shown a total dry plant mass of 0.676 mg at 17 days (408 hours), 4.20 mg at 24 days (576 hours), and 25.9 mg at 31 days (744 hours) after germination. Furthermore, mass-based growth targets include the peak dry weights of the leaves, the seeds, and the stems which have been reported as approximately 163.7 mg (standard deviation 52.0 mg), 127.9 mg (standard deviation 52.7 mg), and 188 mg (standard deviation 39.3 mg), respectively^32^. As the p-ath773 captures both plant growth and loss of seed (and other) mass in the silique ripening stage, the peak mass of each of these tissues has been comparted to this data. In the refined p-ath773 model, the peak masses of the leaves, seeds, and stems have been determined as 153 mg, 100 mg, and 151 mg, respectively, all of which are within one standard deviation of the experimental value^32^ (see the methods section for how tissue masses are determined). These comparisons are summarized in Fig. 6. In summary, *in silico* tissue and plant mass values are similar to *in vivo* data, thus showing strong agreement with respect to plant- and tissue- scale growth trends. This agreement has been achieved by tuning the rate of carbon dioxide and light availability to the plant system^34,36^ which the modeled plant is allowed to utilize as well as by tuning the plant biomass yield (defined as the fraction of plant growth that adds to the plant mass with the remainder addressing litter, tissue repair, and degradation)^37,38^. We have defined both carbon dioxide and light uptakes based on literature, with the former from the carbon assimilation rate^39^ and the Leaf Area Ratio (LAR) of *Arabidopsis*^40^ and the latter from the transmission spectrum of fluorescent light bulbs (used in *in vivo* experiments utilized in the p-ath773 model reconstruction)^36^, the absorption spectra of chlorophyll^37^, and the Leaf Area Ratio of *Arabidopsis*^40^. However, the value of biomass yield (for a given plant across its full lifecycle) has been experimentally identified as between 0.7 and 0.85^37^. Here, to achieve the best alignment between *in silico* and *in vivo* growth patterns, biomass yield has been defined as 0.51. There are several possible reasons for this, which are included in the Discussion section. All files necessary for p-ath773 have been included in the GitHub p-ath773 repository (10.5281/zenodo.3735103). The *in silico* results of the final p-ath773 model can be found in Data S2.

### Flow of water across plant lifecycle

**Figure 7:**
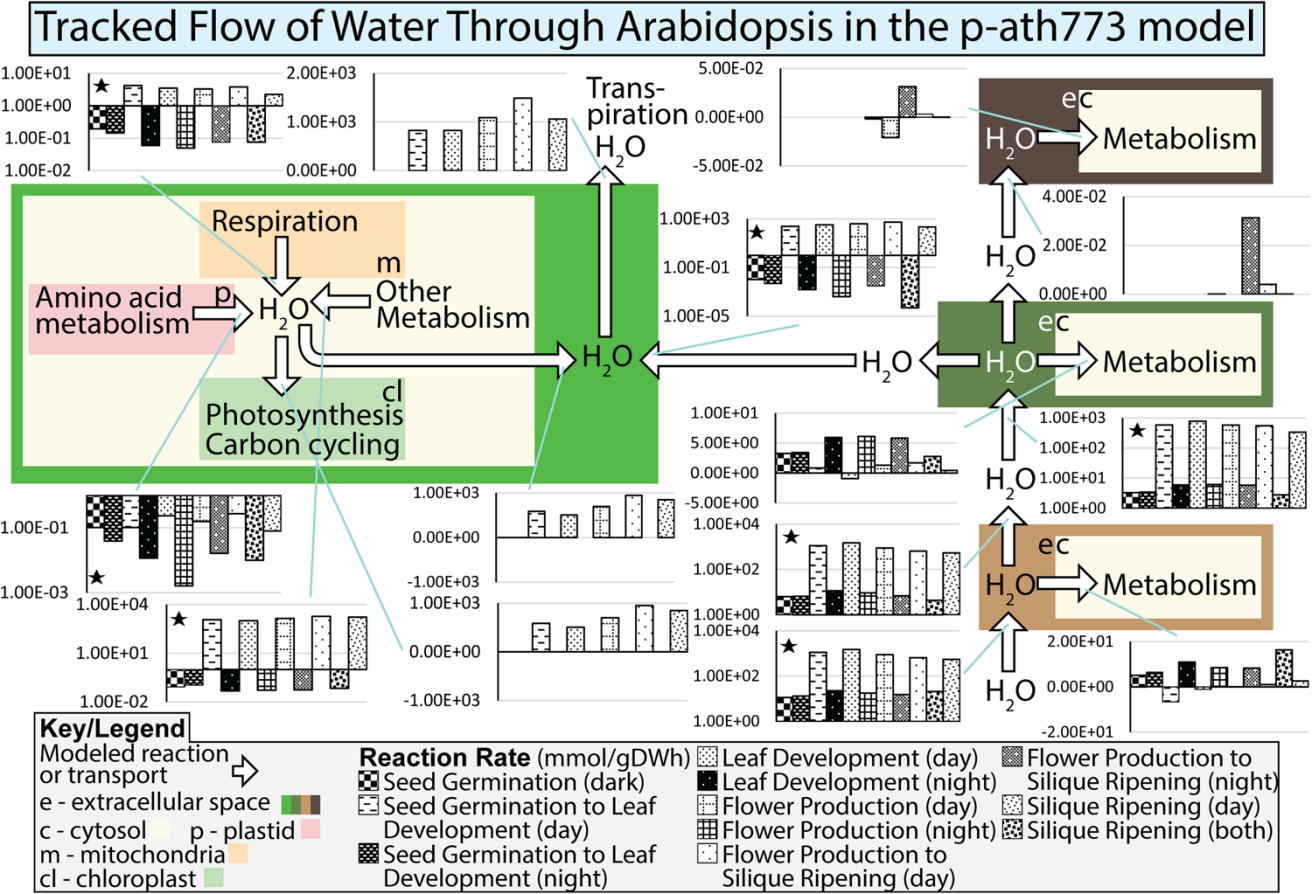
Tracked flow of water through *Arabidopsis* in the p-ath773 model. This figure shows the flow of water (white arrows) through the p-ath773 model by plotting the average reaction rate for each growth stage and each diurnal status of that growth stage, darker bars indicating growth at night and lighter bars indicating growth during the day, to highlight not only the stage-by-stage differences but also the diurnal differences. Flux rates are in units of mmol/gDW·h where gDW (grams Dry Weight) is in units of the dry weight of the individual tissue, rather than the plant as a whole causing incongruity as metabolites are exchanged between tissues as the flux rates must be scaled by the different tissues masses so none of a metabolite is gained or lost between tissue. Further, there are some hydrolysis reactions which occur in the extracellular compartment of each tissue, which accounts for the incongruity in the balance of water in tissue extracellular compartments (such as the in the seed tissue). This is generally a very small amount and therefore was not included in this figure. Further, logarithm-scale y-axes were used where possible (indicated by a small black star) because the day and night flux rates were generally orders of magnitude different.

Important to the life of a plant is the flow of water. Water carries various dissolved nutrients for transport (sugars, amino acids, nitrates, sulfates, et cetera) in addition to meeting the metabolic needs (such as photosynthesis) and physiological needs (such as transpiration) of tissues. Water flow through the plant has been selected as a case study which shows tissue-level insight into the general metabolic and transport processes modeled in p-ath773. The results of this analysis are shown in Fig. 7, where each bar graph represents a specific stage of growth as shown in Fig. 5. As can be seen in Fig. 7, the stem tissue is the center of water transport, accepting water from the root and its own metabolism, and transporting this water to the leaf for its use in photosynthesis and to meet the physiological demands imposed upon the leaf by transpiration in addition to transportation to the seed tissue to meet its metabolic demands. Arrowheads indicate the most common direction of water flow, and negative reaction flux indicates flow in the opposite direction. The p-ath773 model shows that the primary driving force pulling water through the plant is transpiration, and that this driving force results in water flow rates during the light periods of two orders of magnitude higher than that which occurs in the dark periods. This *in silico* observation replicates the physiological water potential gradient along which water flows in plants which is driven by transpiration^40^. Further, the pattern of water flow in the stem tissues being orders of magnitude higher during periods of light is consistent with *in vivo* data of other plant species^41^. While the role of transpiration in plant hydraulics is well known, the p-ath773 model framework in conjunction with the ORKA provides the opportunity to study the contribution of metabolic water to the flow of water in the plant system. In general, as modeled by p-ath773, it appears that root, stem, and seed tissues take up water and utilize it for their own metabolism, acting as water “sinks”. The leaf is however the largest water “sink” in the system since larger amount of water is transpired by the leaf tissue in comparison to that which is used by the metabolism of other tissues. However, the leaf cytosol is a net producer of metabolic water, and the water transported from the cytosol to the extracellular compartment where transpiration is modeled to occur contributes between 60% and 80% of water which is transpired. Major metabolic contributions to the cytosolic water pool appear to be related to various metabolic processes not contained in other tissue models such as nitrate reduction, fatty acid metabolism, and a large number of other metabolic transactions which involve water.

### Sulfur metabolism across plant lifecycle

**Figure 8:**
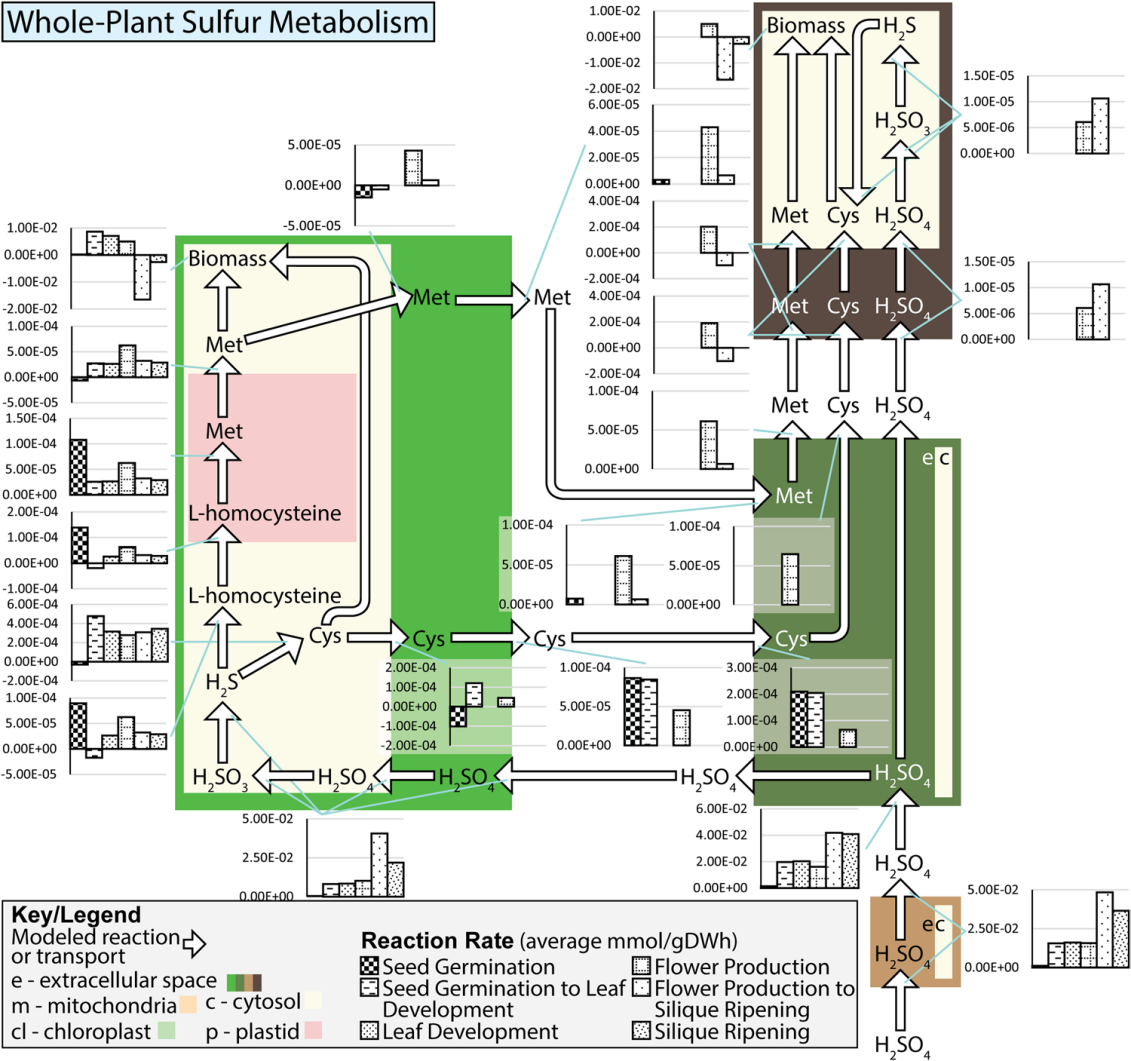
Rate of sulfur-utilizing reactions in *Arabidopsis* in the p-ath773 model. This figure is meant to accompany Fig. 9. This figure shows the evolution of the growth-stage mean reaction rates of reactions which transform or transport sulfur containing compounds in the p-ath773 model through the lifecycle of *Arabidopsis*. Flux rate values (black patterned bars) are in mmol/gDW·h, where **gDW** (**g**rams **D**ry **W**eight) is in units of the dry weight of the individual tissue, rather than the plant as a whole causing incongruity as metabolites are exchanged between tissues as the flux rates must be scaled by the different tissues masses so none of a metabolite is gained or lost between tissue. Further, as sulfur-containing compounds are allowed to be stored in the model by building concentration, reaction rates may not balance.

**Figure 9:**
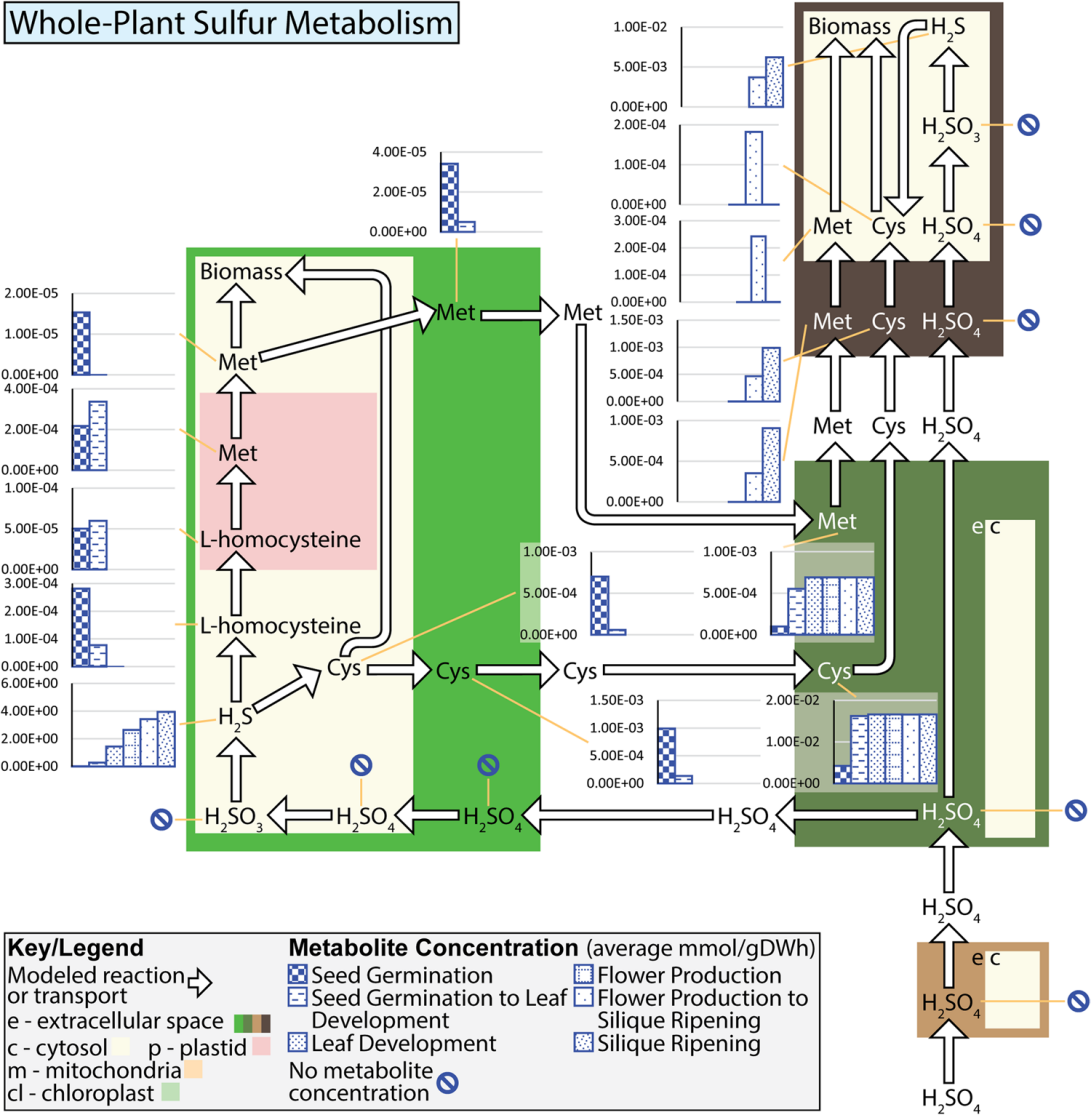
Concentration of sulfur-containing metabolites in *Arabidopsis* in the p-ath773 model. This figure is meant to accompany Fig. 8. This figure shows the evolution of the growth-stage mean concentration of sulfur containing compounds in the p-ath773 model through the lifecycle of *Arabidopsis*. Concentration values (blue patterned bars) are in mmol/gDW.

In addition to tracking the flow of water through the plant, the p-ath773 model has also been used to study and track sulfur metabolism and transport across the tissues and the lifecycle of the plant to provide an example of reaction-level window into the p-ath773 modelled plant metabolism. This has been done to provide unique insight into the core metabolism of a single micronutrient which is not as extensively studied as carbon and nitrogen metabolism^19,27,42^, yet sulfur still is important to plant growth. The results of this analysis are shown in Figs. 8 and 9, where the former reports mean reaction rates and the latter reports mean concentrations for each specific stage of growth as shown in Fig. 5. Sulfur is modeled as passing through the root and stem tissue and being distributed to the leaf and stem tissues. Some sulfur which has been distributed to the leaf tissue will be returned back to the stem, in the form of amino acids for distribution to the seed tissue, with the remainder being used to produce biomass. The seed accepts amino acids and sulfate from the stem tissue to produce biomass. Here it is evident that, in terms of sulfur metabolism, the seed serves as a “sink” tissue, the root as a “source” tissue, and the leaf as an intermediary. As is shown in Fig. 8, the demand by the plant for sulfur is highest in the latter three stages of growth, where seed tissue is present and growing rapidly, or being loosed and metabolic demand from the seed corresponds to the increased maintenance and senescence of the seed and leaf tissues. The presence of seed tissue as a sulfur “sink” also leads to a high flux rate through many reactions in the sulfur metabolism in the leaf as well as transport of sulfur-containing amino acids through the stem tissue. These observations are largely as expected. Unexpected results are those related to the generally high rate of flux through portions of the sulfur metabolism in the leaf during the seed germination growth stage, and the corresponding low fluxes through these pathways in the seed germination to leaf development transition. From closer observations of metabolite concentrations and reactions rates as shown in Figs. 8 and 9 (see Data S3), it appears that there are seemingly random switches between production, storage, and consumption of various metabolites such as L-homocysteine, methionine, and cysteine in the leaf in the early growth stages.

At some places in the Figs. 8 and 9 metabolic maps, there appear some metabolites which have no initial concentration, yet a high mean concentration in the first stage of growth and mean fluxes away from that metabolite. This seems counter-intuitive. For one such metabolite cysteine in the leaf tissue in the first 60 hours after germination (the seed germination stage), the reaction converting hydrogen sulfide to cysteine has positive flux (average positive flux of 2.72E-3 mmol/gDW·h) for 13 of those hours, negative flux (average negative flux of −1.69E-3 mmol/gDW·h) for 22 hours, and no flux for 25. It appears that the no flux points in particular are positioned such that they occur when cysteine concentration is high, skewing the mean concentration upward. Notably, when the stores are used, a number of negative flux rates occur in a row. This skews the average reaction rate downward. It is also shown that cytosolic and extracellular cysteine have high concentrations. This is achieved by near constant interchange of cysteine position through a proton antiport. In the first 60 hours after germination (the seed germination stage) this antiport flows in the direction of the extracellular space 21 of those hours (average flux 0.001337 mmol/gDW · h), in the direction of the cytosol 38 of those hours (average flux −0.00098 mmol/gDW·h), and has no flux only at the first hour when there is as of yet no concentration of cysteine in the cytosol.

## Discussion

In the current work, a novel Optimization- and explicit Runge-Kutta- based Approach (ORKA) to dynamic Flux Balance Analysis (dFBA) has been developed. Inspired by the Static Optimization Approach (SOA) to perform dFBA, it seeks to achieve higher levels of model accuracy and solution stability. ORKA differs from the SOA in that it replaces first-order Taylor-series approximations for biomass and concentration steps with Runge-Kutta- and Trapezoid rule-based integration. This provides lower error floors, from *O*(*h*^2^) in the SOA to *O*(*h*^4^) in the ORKA, depending on the Runge-Kutta method used in the ORKA. ORKA has been developed to be general enough that several different Runge-Kutta methods could be applied to biomass step estimates (Fig. 2A,B) dependent on the error level desired or which could be achieved in the modelled system.

As a test system for ORKA, a multi-tissue core metabolism stoichiometric model of *Arabidopsis thaliana* has been reconstructed (Fig. 3), which includes individual leaf, root, seed, and stem tissues models with unique metabolic roles (Fig. 2). This model, named p-ath773, has defined intra-tissue interactions, interactions with the environment, and certain growth-based parameters defined based on growth stage in an effort to model *Arabidopsis* growth across its lifecycle by defining several growth stages (Fig. 5). Once p-ath773 has been reconstructed, ORKA has then been applied (Fig. 2C) using Heun’s Third Order Rule. When the p-ath773 model using the ORKA (to perform dFBA) is compared to another *Arabidopsis* model utilizing the SOA (to perform dFBA)^19^, the p-ath773 model in theory has at least a three order of magnitude lower error floor due to the smaller step sizes, increased accuracy of the dFBA approach used, and inclusion of two more tissue types. However, similar comparison with the most recent dFBA work on *Arabidopsis* lifecycle^19^ is not entirely possible since these models are quite different in structure, goals, and results. For instance, the mass of the plant for the 6 to 36 days window of time is quite different between p-ath773 and the model produced by Shaw and Cheung (2018)^19^ (see Data S2 for details). In addition, comparing the rate of glutamine synthase in p-ath773 to that of Shaw and Cheung (2018), we find marginal agreement between the two models. One of the primary differences between the models is the direction of the flow of amino acids in the models. While Shaw and Cheung (2018) show nitrate flow from the root to the leaf and then amino acid flow from the leaf to the root, the p-ath773 model synthesizes some amino acids in the roots and those amino acids being transported to the leaf tissue for consumption. Therefore, the direction of amino acid flow is reversed from that of Shaw and Cheung (2018), yet  is similar to what is reported in literature^43–45^. Further, as the biomass equations are different between the two models, the p-ath773 model has a greater demand for amino acids and nitrogen atoms in its biomass composition than does Shaw and Cheung’s (2018). Therefore, by these models having different biomass, different flows of nitrogen, and different biomass composition and demands, it is very difficult to make a worthwhile comparison between the two models on the basis of accuracy as the structure is so different without strongly adapting one model or the other to be more similar to the other. Even though the p-ath773 model lacks a similar model in literature for the purposes of comparison, possibly because different literature sources and goals are used in model reconstructions, it is certain that when ORKA will be applied to a modeling framework comprising of all major tissues and can recapitulate and analyze real plant phenotypes. Further, these differences do not invalidate one model or the other, but rather might consider different metabolic states due to different growth conditions, thereby representing the flexibility of biological systems. In future, OKRA can be applied either by developing more tissue models (e.g., stem and seed) and adding to Shaw and Cheung’s model or extending the p-ath773 model to capture the secondary metabolism, and either approach, carefully informed by literature, could greatly add to knowledge of *Arabidopsis* metabolism.

Using the ORKA to perform dFBA, p-ath773 is able to simulate seven stages of *Arabidopsis* growth (Fig. 5) and showed agreement with literature on plant-scale growth (Fig. 6) and on some reaction-level metabolic characteristics such as transpiration being a driving force of water flow through the plant system (Fig. 7). One point on which there is lesser agreement between p-ath773 and *in vivo* plant-scale data is biomass yield, which is 51% in the p-ath773 model but for most species the value is between 70% and 85% *in vivo*^46^. This disparity is likely due to a few factors. The first is that the literature *in vivo* data generally accounts for factors such as harvesting and animal grazing^37,38^, which is beyond the scope of the p-ath773 model, allowing for more growth. Further, the metabolic costs of root exudates (metabolites exported by the root to support the root microbial community) are not modeled. This is another potentially considerable drain on plant resources which is not modeled in the p-ath773 model.

The modelled flux rates have been used to study the flow of water through the plant system, and in particular to investigate the contributions of metabolic water to that transpired (Fig. 7) and to investigate the whole-plant core metabolism of sulfur (Figs. 8 and 9). In the former case study, the p-ath773 model has showed that metabolic water may contribute significantly to the amount of water transpired, somewhere between 60% and 80% of the total, and that transpiration drives a strong diurnal pattern of water flow. We hypothesize that the metabolic contribution to the amount of water transpired *in vivo* is unlikely to be as significant as shown by the p-ath773 model but is still likely to make some contribution. This is because not all water dynamics are accounted for in the p-ath773 model, including factors such as the amount of water necessary to keep new biomass turgid (since what is modeled is dry weight not wet weight) and the amount of water produced or consumed by the plant’s extensive secondary metabolism. The former shortcoming is common to all SMs rather than to the p-ath773 model in particular, as all such models only model dry weight.

For the sulfur metabolism case study, it has been shown that part of the patterns of sulfur metabolism are as expected such as increased use of and metabolic demand for sulfur when the seed tissue is present. However, some unexpected behavior has also been observed such as higher fluxes through sulfur reactions and comparatively larger concentrations of sulfur-containing metabolites at early growth stages. It is nearly impossible to pinpoint a single cause for the unexpected metabolic behavior of the sulfur metabolism in the early growth stages. This is due to the links between sulfur and energy metabolisms, in that many steps use some type energy molecule. Sulfur metabolism is also closely linked to the proton budget of the plant, in that many transports are proton-coupled. Through links to both the energy metabolism and proton budget, sulfur metabolism is strongly connected with the rest of plant metabolism. Hypothetically, this unexpected metabolic behavior might therefore be advantageous to the plant in energy metabolism and the control of the flow of protons. Particularly in the first two growth stages when the seedling’s endosperm and cotyledons are not fully utilized and are therefore providing some amino acids (though notably not cysteine or methionine), fatty acids, and sugars. As modeled, these stores interact with the extracellular space of the leaf tissue, and often require facilitated transport (usually proton-coupling) into the cytosol for use or catabolism. It is therefore possible that these unexpected behaviors aid in the transport of nutrients from the endosperm, by having standing pools of metabolites which participate in proton-coupled transport to better regulate the cell’s proton budget. This hypothesis is supported by the fact that these unexpected metabolic behaviors are reduced in magnitude as the amount of nutrients uptaken from the endosperm are reduced, and indeed the concentration of metabolites such as cysteine sharply decrease. These unexpected behaviors then appear to cease all together when the endosperm is fully utilized. While the metabolic network of p-ath773 is too convoluted to prove this theory, it does highlight the usefulness of stoichiometric modelling to identify interactions which may be too complex to deduce through non-systems approaches.

While there are a number of constraints applied to the model, such as biomass yield; maintenance and senescence costs; and enforcing mass ratios between tissues, these constraints apply mostly to plant-scale behaviors. These behavioral constraints generally fall into two categories: whole-plant and tissue-tissue interactions. The former generally ensure that the pattern of modeled plant and tissue growth fits that of *in vivo* data. The latter generally ensure that mass balance is maintained when metabolites are transported between tissues since each flux rate is in units of mmol/gDW tissue·h and each tissue is of a different mass. Hence, such conversions are necessary. Other constraints which fall in the category of tissue-tissue interactions ensure that nutrient flow is in a logical and well-known direction (e.g. micronutrients travel up from the roots). Few constraints, with the exception of the enforced diurnal patterns of carbon storage, apply on the reaction rate- or metabolite concentration- levels, leaving a large number of system degrees of freedom at the micro-scale. Therefore, by constraining the macro-scale behavior to what is known, the p-ath773 model can be used to determine what is, or may be, occurring in the plant system with respect to reaction rates or metabolite concentrations. From the allowed uncertainty at the micro-scale level, a study of this level allows investigation of what metabolic processes support and explain the known macro-scale behavior.

This work provides the basis for much future development and sophistication, both in broadening the range of approaches which can be taken to dFBA, and in the potential to use p-ath773 as a basis for modeling other plant systems. Appying ORKA to perform dFBA may provide the framework for other step-by-step dFBA approaches utilizing other ODE solving methods such as Taylor Series, Linear Multistep, or even adaptive step size methods depending on the needs of the modeled system. The current p-ath773 model could be further sophisticated by adding the secondary metabolism of the plant system, which constitutes a significant portion of metabolism in many plant systems. Further, several simplifications have been made regarding tissues, particularly related to seed tissue, at present. For instance, the model currently assumes when the plant is flowering, that flower biomass and metabolism is roughly equivalent to that of the seed. While this results in a simpler model, this model cannot be used to investigate certain metabolic hypothesis such as the cost to the plant resulting from flower pigmentation, pollen, and nectar production. Future work will include developing models for other plant tissues, such as flowers. In addition, as this is a core carbon metabolism model, it is likely quite similar to the core metabolism of other plant systems. Therefore, the p-ath773 model can serve as a basis for the development of lifecycle models for other plant systems, particularly annual eudicots which are of agricultural interest, such as rice (*Oryza sativa*), potatoes (*Solanum tuberosum*), tomatoes (*Solanum lycopersicum*), and soybeans (*Glycine max*).

## Materials and Methods

### Development of the optimization and explicit runge-kutta -based approach to Perform dFBA

#### Static Optimization-Based dFBA Approach (SOA)

The **S**tatic **O**ptimization-Based dFBA **A**pproach (**SOA**) was first introduced in 2002 and is a method for solving for dynamic changes to a model system on a point-by-point basis (where those points are time), as opposed to the Dynamic Optimization-based Approach (DOA) which solves all points simultaneously^28^. The SOA, and variations thereon, have been applied to *Arabidopsis*^19^ and barley^47^ plant models, making it of particular interest for the study of plant metabolism. The SOA is defined as follows^28^:

1 $$Maximize\,{v}_{biomass,t}\,(or\,some\,other\,suitable\,objective\,function)$$

2 $$\begin{array}{cc}{z}_{i,t+\Delta t}\ge 0 & \forall t\in T;i\in I{\prime} \end{array}$$

3 $${v}_{j,t}^{LB}\le {v}_{j,t}\le {v}_{j,t}^{UB}$$

4 $$\begin{array}{cc}{z}_{i,t+\Delta t}={z}_{i,t}+\sum _{j}{S}_{ij}{v}_{j,t}\Delta t & \forall t\in T;i\in I{\prime} \end{array}$$

5 $$\begin{array}{cc}{Y}_{t+\Delta t}={Y}_{t}+{v}_{biomass}{Y}_{t}\Delta t & \forall t\in T\end{array}$$

$$other\,constraints$$

where symbols used are defined in the caption of Fig. 2, in the Results section, and in the “Symbols Used” section of Text S1. Note that *v*_*biomass*_ indicates the rate of reaction for the biomass production and is equivalent to the growth rate *μ*_*t*_ which will be used hereafter. It should also be noted that biomass concentration, *Y*_*t*_, is actually an element of the *I*′ set (the set of metabolites whose concentration is tracked). However, Eq. (5) is included here for *Y*_*t*_ to be consistent with definitions of SOA in previous works^28^. This is also necessary due to the fact that biomass concentration is of particular interest in stoichiometric modeling efforts. Therefore, even though Eq. (4) simplifies to Eq. (5), Eq. (5) is still explicitly stated. This simplification is accomplished by first recognizing that there is only a single *S*_*biomass*,*j*_ that is non-zero, namely *S*_*biomass*,*biomass*_ which has a value of 1. Making the substitution, the RHS of Eq. (4) reduces to *z*_*biomass*,*t*_ + *v*_*biomass*_*z*_*biomass*,*t*_Δ*t*. Secondly, by recognizing that *Y*_*t*_ is equivalent to *z*_*biomass*,*t*_ and making this substitution on both sides of Eq. (4), Eq. (4) reduces to Eq. (5).

The mass step taken at each time point in the SOA method, as shown in Eq. (5), is derived from the Taylor series expansion of *e*^*μx*^ around 0. The exponential formulation comes from the fact that the growth rate determined by a SM of metabolism is an exponential growth rate defined by the following differential equation^8^.

6 $$\frac{d{Y}_{t}}{dt}={\mu }_{t}{Y}_{t}$$

whose solution can be represented as follows:

7 $${Y}_{t+\Delta t}={Y}_{t}{e}^{\mu t}$$

to derive Eq. (5) that is used to advance biomass in the SOA, the first-order Taylor series expansion of *e*^*μt*^ is used. Recall that the Taylor series expansion of *f*(*t*) around point *a* is defined as follows.

8 $$\begin{array}{rcl}f(t) & = & f(a)+f{\prime} (a)(t-a)+\frac{f{\prime\prime} (a)}{2!}{(t-a)}^{2}\\  &  & +\frac{{f}^{(3)}(a)}{3!}{(t-a)}^{3}+\ldots +\frac{{f}^{(n)}(a)}{n!}{(t-a)}^{n}\end{array}$$

it is well known that *f*(*x*) = *e*^*μt*^ then *f*^(*n*)^(*x*) = *μ*^*n*^*e*^*μt*^ ∀*n*, this Taylor series expansion may be simplified as follows.

9 $$f(t)={e}^{a}+\mu {e}^{a}(t-a)+\frac{{\mu }^{2}{e}^{a}}{2!}{(t-a)}^{2}+\frac{{\mu }^{3}{e}^{a}}{3!}{(t-a)}^{3}+\ldots ++\frac{{\mu }^{n}{e}^{a}}{n!}{(t-a)}^{n}$$

finally, since this Taylor series expansion is around *a* = 0, the Taylor series expansion becomes what follows (knowing that *e*^0^ = 1).

10 $$f(t)=1+\mu t+\frac{{\mu }^{2}{t}^{2}}{2!}+\frac{{\mu }^{3}{t}^{3}}{3!}+\ldots ++\frac{{\mu }^{n}{t}^{n}}{n!}$$

in the standard SOA formulation, only the first term is used in the approximation of *e*^*μt*^, therefore:

11 $$g(t)=1+\mu t+O({t}^{2})$$

multiplying through by *Y*_*t*_ gives the biomass steps which are used by the SOA, namely Eq. (5). Therefore, the error in mass step estimates in the standard SOA method is on the order of the timescale squared, e.g. *O*(*h*^2^). One additional assumption is made in Eq. (4). The differential equation which describes the change in concentration of a specific metabolite is shown below.

12 $$\frac{d{z}_{i}}{dt}=\sum _{j\in J}{S}_{ij}{v}_{j,t}$$

by using the separation of variables as the solution method,

13 $$d{z}_{i}=\sum _{j\in J}{S}_{ij}{v}_{j,t}dt$$

14 $$d{z}_{i}=\sum _{j\in J}{S}_{ij}{\int }_{{t}_{o}}^{{t}_{0}+\Delta t}{v}_{j,t}dt+{C}_{1}$$

to derive Eq. (4) (used in the SOA) from Eq. (14), it must be assumed that for each time step (Δ*t) v*_*j*,*t*_ is constant.

#### Optimization- and explicit Runge-Kutta-based dFBA Approach (ORKA)

The dFBA method developed in this work is similar to SOA in the sense that both solve time points in a step-by-step and cumulative fashion. The **O**ptimization- and explicit **R**unge-**K**utta-based dFBA **A**pproach (**ORKA**) differs in that different approximations are used in the solutions attempts to increase the accuracy of the estimation of the concentration and mass steps.

1 $$Maximize\,{\mu }_{t}$$

2 $$\begin{array}{cc}{z}_{i,t+\Delta t}\ge 0 & \forall t\in [{t}_{o},{t}_{f}];i\in I{\prime} \end{array}$$

3 $${v}_{j,t}^{LB}\le {v}_{j,t}\le {v}_{j,t}^{UB}$$

15 $$\begin{array}{cc}{z}_{i,t+\Delta t}={z}_{i,t}+\sum _{j}{S}_{ij}{\Gamma }_{{\rm{j}},{\rm{t}}} & \forall t\in [{t}_{o},{t}_{f}];i\in I{\prime} \end{array}$$

16 $$\begin{array}{cc}{Y}_{t+\Delta t}={Y}_{t}+\Delta t{\frac{d{Y}_{t}}{dt}}_{rkest} & \forall t\in [{t}_{o},{t}_{f}]\end{array}$$

$$other\,constraints$$

where ${\frac{d{Y}_{t}}{dt}}_{rkest}$ represents the Runge-Kutta based estimate for the mass step of the model, and Γ_j,t_ represents an estimate of the integral of *v*_*j*,*t*_. Equation (15) expands on the accuracy of metabolic concentration estimates by leaving the integral term present and by removing the assumption that the reaction rate is time-independent in the time step concerned. This equation will estimate the integral using the multiple-applications Trapezoidal Rule of integration with the generalized explicit Runge-Kutta method. Equation (16) makes a more accurate estimate of the change in mass by using a Runge-Kutta method to better estimate the size of the mass step. With the notation altered to be more consistent with this work, the generic n^th^ order Runge-Kutta method is presented below.

17 $$\frac{dY}{dt}=f(t,{Y}_{t})$$

18 $${Y}_{t+\Delta t}={Y}_{t}+(\mathop{\sum }\limits_{n=1}^{N}{b}_{n}{k}_{n})\Delta t$$

19 $${Y}_{t+\Delta t}-{Y}_{t}=(\mathop{\sum }\limits_{n=1}^{N}{b}_{n}{k}_{n})\Delta t={\frac{dY}{dt}}_{rkest}$$

20 $${k}_{1}=f(t,{Y}_{t})$$

21 $${k}_{2}=f(t+{c}_{2}\Delta t,{Y}_{t}+{a}_{21}{{\rm{k}}}_{1}\Delta t)$$

22 $${k}_{3}=f(t+{c}_{3}\Delta t,{Y}_{t}+({a}_{21}{{\rm{k}}}_{1}+{a}_{32}{k}_{2})\Delta t)$$

$$\vdots $$

23 $${k}_{n}=f(t+{c}_{n}\Delta t,{Y}_{t}+\Delta t\mathop{\sum }\limits_{j=1}^{i-1}{a}_{ij}{k}_{j})$$

where, for an explicit Runge-Kutta method, the above method constants (*a*_*nm*_, *b*_*n*_, and *c*_*n*_) are represented in a triangular Butcher tableau such as shown below in Fig. 2B. As previously stated, biomass growth is exponential as shown in Eq. (6).

6 $$\frac{d{Y}_{t}}{dt}={\mu }_{t}{Y}_{t}$$

Therefore:

24 $$\frac{d{Y}_{t}}{dt}=f(t,{Y}_{t})={\mu }_{t}{Y}_{t}$$

however, since the value of *μ*_*t*_ is calculated by performing FBA on an SM, the SM must be solved for each *k*_*n*_ (Runge-Kutta derivative estimate for step *n*). Therefore, by this necessity, we would also know reaction rates at each time point where a *k*_*n*_ is solved for, namely, *v*_*j*,*t*_, *v*_*j*,*t* +_ *c*_2_Δ*t*, …, ${v}_{j,t+{c}_{{n}_{f}}\Delta t}$. Returning to Eq. (15), should the time points at which *k*_*n*_ occurs be equally spaced, then the multiple-application Trapezoidal Rule (i.e., the area of multiple trapezoids is used to estimate the integral rather than a single trapezoid) might be applied to solve the integral presented in Eq. (15). Evenly spaced points at which *k*_*n*_ values are calculated is defined that if ${c}_{{n}_{f}}-{c}_{{n}_{f}}$_−1_ = ${c}_{{n}_{f}-1}$ −${c}_{{n}_{f}-2}$ = … = *c*_2_−*c*_1_ is true, the points at which *k*_*n*_ are calculated are evenly spaced. Further, if ${c}_{{n}_{f}}$  ≠ 1, then 1−${c}_{{n}_{f}}$ = *c*_2_−*c*_1_ must also be true. This may seem like highly specific criterion; however, several specific Runge-Kutta methods or rules fit these descriptions. These include the explicit midpoint method, Heun’s Method, Heun’s Third Order Rule, Kutta’s Third Order Rule, and the 3/8-Rule Fourth Order Method, among others. The Butcher tableaus for these methods can be found in Fig. 2B. Using any of the above-mentioned methods, Eq. (15) could be restated as follows.

25 $$\begin{array}{cc}{z}_{i,t+\Delta t}={z}_{i,t}+\sum _{j}{S}_{ij}{\Gamma }_{{\rm{j}},{\rm{t}}} & \forall t\in T;i\in I{\prime} \end{array}$$

26 $${\Gamma }_{j,t}={{\rm{c}}}_{1}\Delta t\frac{{v}_{j,t}+{\sum }_{n\in N-({n}_{f})}^{n-1}{v}_{j,{t}_{n}}+{v}_{j,t+\Delta t}}{2({\sum }_{n\in N}1)}$$

27 $${t}_{n}={t}_{0}+{c}_{n}\Delta t$$

28 $${Y}_{{t}_{n}}={Y}_{{t}_{0}}+\Delta t\mathop{\sum }\limits_{a=1}^{n-1}{a}_{na}{k}_{a}$$

the only unknown quantity above is the Runge-Kutta estimate for the mass step (*v*_*j*,*t* + Δ*t*_). As the exact value of *z*_*i*,*t* + Δ*t*_ may be necessary to calculate *v*_*j*,*t* + Δ*t*_, it will be assumed that *v*_*j*,*t* + Δ*t*_ is equal to the arithmetic mean of the reaction rates in the *n* time points used in the Runge-Kutta method selected plus the starting point. Therefore, Eq. (26) may be rewritten as follows.

29 $$\begin{array}{cc}{\Gamma }_{j,t}={{\rm{c}}}_{1}\Delta t\frac{{v}_{j,t}+2\sum _{n\in N}{v}_{j,{t}_{n}}+\frac{{v}_{j,t}+\sum _{n\in N}{v}_{j,{t}_{n}}}{({\sum }_{n\in N}1)+1}}{2({\sum }_{n\in N}1)} & if\,{c}_{{n}_{f}}\ne 1\end{array}$$

note that this correction to Γ_*j*,*t*_ only applies when *c*_*nf*_ ≠ 1. Otherwise, the case when ${c}_{{n}_{f}}$ = 1 removes the need for an arithmetic estimate of the final data point and the following equation might be used.

30 $$\begin{array}{cc}{\Gamma }_{j,t}={{\rm{c}}}_{1}\Delta t\frac{{v}_{j,t}+2\sum _{n\in N-\{{n}_{f}\}}{v}_{j,{t}_{n}}+{v}_{j,{t}_{{n}_{f}}}}{2({\sum }_{n\in N}1)} & if\,{c}_{{n}_{f}}=1\end{array}$$

the advantage of using a multiple application Trapezoidal rule in estimating the integral in Eq. (15) is that the assumption of a constant reaction rate over the time step may be relaxed and that the error for the multiple application trapezoidal rule is *O*(Δ*t*^3^). From this, the ORKA method can be represented as follows.

$${\rm{For}}\,{\rm{each}}\,{\rm{time}}\,t\in [{t}_{0},{t}_{f}]:$$

1 $$Maximize\,{\mu }_{t}$$

2 $$\begin{array}{cc}{z}_{i,t+\Delta t}\ge 0 & \forall i\in I{\prime} \end{array}$$

3 $$\begin{array}{cc}{v}_{j,t}^{LB}\le {v}_{j,t}\le {v}_{j,t}^{UB} & \forall j\in J\end{array}$$

19 $${\frac{d{Y}_{t}}{dt}}_{rkest}=(\mathop{\sum }\limits_{n=1}^{R}{b}_{n}{k}_{n})\Delta t$$

16 $${Y}_{t+\Delta t}={Y}_{t}+\Delta t{\frac{d{Y}_{t}}{dt}}_{rkest}$$

26 $${z}_{i,t+\Delta t}={z}_{i,t}+\sum _{j}{S}_{ij}{\Gamma }_{{\rm{j}},{\rm{t}}}\,\forall i\in I{\prime} $$

29 $${\Gamma }_{j,t}=({c}_{2}-{{\rm{c}}}_{1})\Delta t\frac{2\sum _{n\in N}{v}_{j,{t}_{n}}-{v}_{j,{t}_{{n}_{0}}}+\frac{\sum _{n\in N}{v}_{j,{t}_{n}}}{{\sum }_{n\in N}1}}{2({\sum }_{n\in N}1)}\,if\,{c}_{nf}\ne 1$$

30 $${\Gamma }_{j,t}=({c}_{2}-{{\rm{c}}}_{1})\Delta t\frac{2{\sum }_{n\in N}{v}_{j,{t}_{n}}-{v}_{j,{t}_{{n}_{0}}}-{v}_{j,{t}_{{n}_{f}}}}{2({\sum }_{n\in N}1)}\,if\,{c}_{nf}=1$$

$$other\,constraints\,such\,as\,mass\,balance\,and\,nutrient\,uptake\,which\,are\,necessary\,for\,modeling$$

For each Runge-Kutta Step *n* ∈ *N*, denoting the current time as *t*_*c*_

20 $${k}_{n}=f({t}_{n},{Y}_{{t}_{n}})\,\forall n\in N$$

27 $${t}_{n}={t}_{c}+{c}_{n}\Delta t\,\forall n\in N$$

28 $${Y}_{{t}_{n}}={Y}_{{t}_{0}}+\Delta t\mathop{\sum }\limits_{a=1}^{n-1}{a}_{na}{k}_{a}\,\forall n\in N$$

to this point, the ORKA framework is deliberately general enough that any Runge-Kutta method which satisfies the criteria related to the values of *c*_1_ through *c*_*nf*_ might be selected. It should be noted that the multiple application Trapezoidal rule has an error floor in the order of *O*((*c*_2_−*c*_1_)*h*^3^). Therefore, the integral estimate will have lower error than third-order Runge Kutta methods, which generically have a global error of *O*(*h*^3^) because of their use of individual steps of the Runge Kutta method rather than the full time step. The integral estimate will be the limiting accuracy factor if fourth order Runge-Kutta methods (such as the 3/8-rule fourth order method) or better are used which have a global error of *O*(*h*^4^).

#### Limitation of ORKA to explicit Runge-Kutta Methods

Generally, Runge-Kutta methods are defined as either implicit or explicit. A method is defined as explicit if *a*_*ij*_ = 0∀*j* ≥ *i*, and implicit if this is not the case. Recall that *a*_*ij*_ defines the dependence of one derivative estimate step on another, as shown below.

23 $${k}_{n}=f(t+{c}_{n}\Delta t,{Y}_{t}+\Delta t\mathop{\sum }\limits_{j=1}^{i-1}{a}_{ij}{k}_{j})$$

if a method is explicit, each estimate depends only on previous estimates, whereas if a method is implicit, it may rely on future estimates or even itself. Implicit Runge-Kutta methods therefore are more difficult to implement, requiring the solution of a non-linear system of equations. In this work, *f*(*t*,*Y*) is the solution of a large system of under-defined linear equations (the stoichiometric model) where the best solution is selected by optimization. Using an implicit Runge-Kutta method would require the full model to be included in an under-defined non-linear system of equations and then to be solved by non-linear programming approaches. These approaches neither guarantee that a solution would be found nor a solution found would be optimal^48^. The sharply increased computational costs of using an implicit method combined with the complexity of implementation and the non-guarantee of an optimal solution has made implicit Runge-Kutta methods not worthwhile or attractive for implementation.

### Overview of the reconstruction of core metabolic models of leaf, root, seed, and stem tissues

The seed tissue has been modeled primarily based on a published MFA work^29^ allowing an accurate reconstruction of the central carbon metabolism of the seed. Next, the leaf tissue has been reconstructed as a phototrophic tissue to supply carbon to the seed tissue. We next have reconstructed the root model to provide a mechanism for the uptake of water and micronutrients necessary for plant growth. Finally, we have reconstructed the stem model to provide a logical link between the tissues. Additional works that have been used in the reconstruction of tissue models can be found in Data S1.

#### The seed tissue model

The general workflow which has been used for the development of the four core tissue models is illustrated in Fig. 3. The seed model has been developed first, with the central metabolic pathways based on a Metabolic Flux Analysis (MFA) of four seed genotypes published previously^29^. We then manually have filled gaps in this model with reactions based on literature and genomic evidence^7,49^ or with reactions being necessary for ensuring model connectivity. The stoichiometric coefficients of biomass precursors have been determined using sink reactions, dry biomass weight composition, and amino acid mass ratios provided in a previous work^29^ (see Text S1). The resultant seed tissue model focuses on storage, respiration, and growth, and consists of 418 reactions, 577 genes, and 390 metabolites GitHub p-ath773 repository (10.5281/zenodo.3735103) for this work.

#### The leaf tissue model

Next, we have reconstructed the leaf model by taking common reactions/pathways from the seed model and adding metabolic pathways for amino acids that are not synthesized in the seed. In addition, other leaf-specific pathways such as photosynthesis, carbon fixation, and gluconeogenesis and necessary transport reactions have also been added. We then have developed the biomass equation for the leaf tissue using that of a previously published *Arabidopsis* model^23^ (see Text S1), with minor adjustments. First, since the p-ath773 model is designed to focus on core metabolism, secondary metabolites were removed from biomass equation. Second, it was noticed that the amino acid histidine was missing as a primary metabolite from biomass composition. Histidine was added into the leaf biomass in proportion to other amino acids (See Data S1)^29^. The resultant leaf tissue model has focused on photosynthesis, respiration, gas exchange, fatty acid synthesis, and growth, and contains of 517 reactions, 666 genes, and 463 metabolites. We have included the leaf model in the GitHub p-ath773 repository (10.5281/zenodo.3735103).

#### The root and stem tissue models

We have constructed the root and stem models, similarly, by extracting common reactions/pathways from the seed model and adding necessary and root-/stem-specific transport and exchange reactions. Then exchange reactions have been added to allow the root to be linked to micronutrient uptake processes from the soil and the stem to be involved in inter-tissue transport processes. In the absence of *Arabidopsis*-specific estimates, the dry weight composition of switchgrass (*Panicum virgatum*) root and stem^31^ have been assumed to be equivalent to the biomass composition of these tissues in *Arabidopsis*. Thus, we have found the biomass of root and stem tissues to be composed entirely of carbohydrates. The resultant root tissue model focuses on nutrient uptake, transport, and growth, consisting of 149 reactions, 324 genes, and 149 metabolites, while the stem tissue model focuses on transport and growth, consisting of 167 reactions, 291 genes, and 154 metabolites. We have included the root and stem models in the GitHub p-ath773 repository (10.5281/zenodo.3735103).

#### Confidence scoring

We have defined reaction confidence scores in a manner consistent with a previously published protocol^7^. Confidence scores are integer values between 0 and 4, with higher values corresponding to higher confidence in the inclusion for a given reaction. In the scoring system used, 0 corresponds to an unevaluated reaction; 1 to a reaction included for modeling necessity; 2 to evidence from physiology or a genome annotation; 3 from knock-in knock-out *in vivo* experiments; and 4 for direct biochemical data giving evidence for that metabolic function. The distribution of confidence scores in the component tissue models of p-ath773 can be found in Fig. 2B through 2E. As is shown in these figures, score 3 evidence was not used as score 2 evidence was considered sufficient for model reconstruction and, if greater confidence was required, direct biochemical data could be found since *Arabidopsis* is a model system. Additional information on confidence scoring of the p-ath773 model can be found in Text S1.

#### Curation of these four tissue models

All reactions in all four models have been balanced both in terms of elements and charge. Thermodynamically infeasible cycles have also been resolved by removing reactions, breaking composite reactions, and adding metabolic costs to transport reactions. For all four tissue models, GPR links have been established through a largely automated workflow utilizing the KEGG API^50^ for the majority of reactions using the code included in the GitHub p-ath773 repository (10.5281/zenodo.3735103). This has been followed by having manually curated the GPR links and/or inclusion rational of reactions with non-KEGG identifiers. This information can be found in Data S1. The count of tissue model reactions present in KEGG-defined pathways is shown in Fig. 2A, giving an overview of each tissue models’ metabolic capabilities. The code developed to create these figures is included in the GitHub p-ath773 repository (10.5281/zenodo.3735103). The results of this automated workflow can be found in Data S2. Sources for reactions included in leaf, root, seed, and stem models are shown in Fig. 4B through 4E, respectively through confidence scoring (see Text S1).

### Linking tissue models utilizing metabolic constraints and ORKA

#### Application of ORKA to the p-ath773 model

The application of ORKA to the p-ath773 model is complicated by the fact that there is not a single biomass reaction, but rather four separate reactions, one for each tissue modeled: leaf, root, seed, and stem. Therefore, for the mass of the whole plant, the basic differential equation which defines the change in system mass with time is stated below.

29 $$\frac{d{Y}_{t}}{dt}=f(t,{Y}_{t})={\mu }_{plant,t}{Y}_{t}$$

where here *Y*_*t*_ indicates whole-plant mass. This could require some complex hand calculations to determine the value of the RHS of Eq. (29) since *μ*_*plant*,*t*_ is not calculated in the p-ath773 model, instead only individual tissue biomass growth rates are determined. This leads to a branching point in how to apply the ORKA method to the p-ath773 model: whether to use whole plant mass or individual tissue masses as the basis of biomass calculations. On one hand, as already stated, the biomass of the whole-plant system could be tracked, which would result in a more complex RHS and formulation of *f*(*t*, *Y*_*t*_). On the other hand, the biomass of each plant tissue can be tracked individually as stated in the following equation.

30 $$\frac{d{Y}_{\theta ,t}}{dt}={\mu }_{\theta ,t}{Y}_{\theta ,t}\,\theta \in \Theta $$

it has been decided to use the former method of tracking biomass because solving Eq. (30) at *Y*_*θ*,0_ = 0 yeilds only 0 as a solution. This can be shown in that the generic solution to Eq. (30) is formulated as follows.

31 $${Y}_{\theta ,t+\Delta t}={Y}_{\theta ,t}{e}^{{\mu }_{\theta ,t}t}\,\theta \in \Theta $$

by the multiplicative identity rule, *Y*_*θ*,*t* + Δ*t*_ = 0 if and only if *Y*_*θ*,*t*_ = 0 since no exponential function can take the value of 0. This presents two issues if this is the method of advancing tissue biomass: (i) no tissue can either appear in the system that is not there from the beginning, and (ii) no tissue can be removed from the system. This is particularly problematic for a plant system since certain tissues appear and are removed after the plant reaches certain levels of maturity, perhaps most notably flowers and seeds. Therefore, while more complex, determining the value of the right-hand side of Eq. (29) is preferable. Text S1 details the calculation of the RHS of Eq. (20). The end-result of this calculation is as follows.

32 $$\frac{d{Y}_{t}}{dt}=\frac{{e}^{{\mu }_{leaf,t}}{Y}_{leaf,t}}{{x}_{leaf,t}}\left[{x}_{leaf,t}{\mu }_{leaf,t}+\frac{d{\mu }_{leaf,t}}{dt}+{\xi }_{t}\right]$$

33 $$\begin{array}{rcl}{\xi }_{t} & = & {x}_{root,t}{\mu }_{root,t}+{x}_{seed,t}{\mu }_{seed,t}+{x}_{stem,t}{\mu }_{stem,t}\\  &  & +{x}_{leaf,t}({\mu }_{root,t}{\zeta }_{t}+{\mu }_{seed,t}{\rho }_{t}+{\mu }_{stem,t}{\delta }_{t})\frac{d{s}_{t}}{dt}+{x}_{root,t}\frac{d}{dt}(\mathrm{ln}({\omega }_{t}))\\  &  & +{x}_{seed,t}\frac{d}{dt}(ln({\eta }_{t}))+{x}_{stem,t}\frac{d}{dt}(\mathrm{ln}({\lambda }_{t}))\end{array}$$

34 $${\omega }_{t}=\frac{{x}_{root,t}{M}_{leaf,0}}{{x}_{leaf,t}{M}_{root,0}}$$

35 $${\eta }_{t}=\frac{{x}_{seed,t}{M}_{leaf,0}}{{x}_{leaf,t}{M}_{seed,0}}$$

36 $${\lambda }_{t}=\frac{{x}_{stem,t}{M}_{leaf,0}}{{x}_{leaf,t}{M}_{stem,0}}$$

37 $${\zeta }_{t}=\frac{{c}_{root}({c}_{leaf}{s}_{t}+{x}_{leaf,0})-{c}_{leaf}({c}_{root}{s}_{t}+{x}_{root,0})}{{c}_{leaf}^{2}{s}_{t}^{2}+2{c}_{leaf}{s}_{t}{x}_{leaf,0}+{x}_{leaf,0}^{2}}$$

38 $${\rho }_{t}=\frac{{c}_{seed}({c}_{leaf}{s}_{t}+{x}_{leaf,0})-{c}_{leaf}({c}_{seed}{s}_{t})}{{c}_{leaf}^{2}{s}_{t}^{2}+2{c}_{leaf}{s}_{t}{x}_{leaf,0}+{x}_{leaf,0}^{2}}$$

39 $${\delta }_{t}=\frac{{c}_{stem}({c}_{leaf}s+{x}_{leaf,0})-{c}_{leaf}({c}_{stem}s+{x}_{stem,0})}{{c}_{leaf}^{2}{s}^{2}+2{c}_{leaf}s{x}_{leaf,0}+{x}_{leaf,0}^{2}}$$

note that the above solution makes explicit use of the assumption that the time step used is one hour and that the growth rate unit is inverse hour. Further, an effort has been made in the above formulation to minimize the number of variables used and only the growth rate of the leaf tissue has been included (as the other growth rates of other tissues can be readily calculated from that of the leaf). The number of parameters has not been minimized since readability and compactness have been considered more important than minimizing the number of equations or parameters. This framework requires estimates of time derivatives for some parameters such as seeding level (*s*_*t*_) and several other quantities in Eq. (33). For all derivative estimates needed, a second-order accurate backwards finite-difference method has been used, as solutions to points previous in time will be known while solutions to points forward in time are unknown. For all parameters for which a derivative estimate needs be made, we have used the following equation where *ϕ* stands in for any parameter above.

40 $$\frac{d{\phi }_{t}}{dt}\approx \frac{3{\phi }_{t}-4{\phi }_{t-h}+{\phi }_{t-2h}}{2h}+O({h}^{2})$$

Note that the trapezoid rule estimates also rely on even step sizes. This allows for smaller error in both of these estimates. When calculating the derivatives for the first two time points, it is assumed that the parameter values are the same as that for the first time point, e.g. it is assumed that *ϕ*_−2*h*_ = *ϕ*_−*h*_ = *ϕ*_0_. This derivative estimate, particularly the equidistant points requirement, is an important consideration in choosing the particular Runge-Kutta method used in this work. These calculations can be found in the “calculate parameters needed to solve next step equation” lines of the pseudocode in Fig. 2A.

Given that the steps taken for the Runge-Kutta method have equally spaced values of *c*_*n*_ and that *O*(*h*^2^) where *h* = (*c*_2_ − *c*_1_)Δ*t* is the order of error for the estimation of the backward derivative, this limits the order of the Runge-Kutta methods which can be chosen for increased accuracy benefits. For instance, a third order Runge-Kutta method has a global error of *O*(h^3^), less than that of the backward derivative estimate. In this case, choosing any Runge-Kutta method which is higher than third order would merely add complexity with no benefits in terms of the error of the solution. Therefore, a third order Runge-Kutta method has been chosen for implementation with the p-ath773 model. Two commonly-used such methods are Heun’s and Kutta’s third order rules shown in Fig. 2B. We have chosen Heun’s third order rule for this application as it provides greater accuracy in the integral estimates (e.g. $h=1/3\cdot \Delta t$ as opposed to $h=1/2\cdot \Delta t$) and there are no negative values in the matrix of parameter *a*_*ij*_ of the Butcher tableau which have caused errors in earlier implementations of Kutta’s third-order rule to the p-ath773 model (but would not in the current model).

Given that the limiting order of error in this system is the error in parameter derivative estimates, this will be the order of error for ORKA calculations for the p-ath773 model. As the value of h is one third of that of the time step (Δ*t)*, we can state that the error would be on the order of $O(1/9\cdot \Delta {t}^{2})$. This is a significant improvement in the error over previously implementation of dFBA on the *Arabidopsis* models including that of Shaw and Cheung (2018)^19^, which by reason of using SOA and a time step of one day (as opposed to one hour) results in much higher error potential. Calculating on the basis of hours the big *O* error ratio between ORKA and the SOA used by Shaw and Cheung (2018)^19^ the is approximately 1:5184. This will provide two distinct advantages to the p-ath773 model. First, higher accuracy for calculations due to smaller step size and lower errors associated with approximations used. Second, increased solution stability, so that more solution steps may be taken without ballooning error.

### Other constraints in p-ath773’s ORKA framework

The tissue models were linked using techniques similar to a well-known computational framework known for modeling microbial communities^51^. This involved specifying how metabolites are allowed to move between tissues in logical ways, which will be described in greater detail later in this section. This framework includes a whole-plant objective which specifies fluxes in each tissue to maximize or minimize. Next, literature information including embryo mass^30,34^, initial tissue masses^33^, growth stages^32^, time points at which growth stages occur^32^, constraints to link tissue growth rates to appropriate tissue ratios, transpiration^52–54^, leaf surface area^40^, usability of provided light^36,37^, and defining changes in tissue mass ratios^32,34^ has been integrated into these models, which are typically overlooked in most other SMs. In this work, we have decided to simulate *Arabidopsis* biomass across 61 days (1464 hours) of growth, as all plant seeds are dispersed by approximately day 61, and after which *in vivo* data on plant growth and mass is sparse^32^. More specific details can be found in the following sub-sections. The full optimization-based framework used in this work has been provided in the GitHub p-ath773 repository (10.5281/zenodo.3735103) associated with this work.

#### Enforcing mass balance

As concentration is tracked using the ORKA method, the mass balance for the system need not be a strict equality, but rather metabolites should be allowed to be stored and that store should be allowed to be used up. To this end, the mass balance has been defined as follows:

41 $$\sum _{j\in J}{S}_{ij}{v}_{j}\ge -{z}_{i,{t}_{n}}\,{\rm{\forall }}i\in I{\rm{{\prime} }}$$

42 $$\sum _{j\in J}{S}_{ij}{v}_{j}\ge 0\,{\rm{\forall }}i\in I-I{\rm{{\prime} }}$$

these equations ensure that metabolites might be stored (i.e., more metabolite is produced than consumed) in all cases or available metabolite concentrations might be utilized. When such a concentration is utilized, the LHS of Eq. (41) becomes negative. Hence, the metabolite is consumed at a greater rate than it is produced, yet still obeys the law of concentration of mass by using up the present stores of that metabolite to account for the difference. These equations do not, however, guarantees against the model producing infeasible reaction rates, as large rates of metabolite production are allowable under Eq. (41). To limit the amount of any metabolite stored at a given time point, the following constraint is implemented.

43 $$\sum _{j\in J}{S}_{ij}{v}_{j}\le 10\,{\rm{\forall }}i\in I{\rm{{\prime} }}$$

this limits the rate of any metabolite’s storage to 10 mmol per gDW tissue per hour and represents that maximum of the allowable violation of the mass balance in the direction of metabolite storage. Such an allowable violation is allowed in all dFBA models where changes in metabolite concentration are allowed. This value is an arbitrary limit on the rate of allowed metabolite storage per hour since it seemed logical to create some limit on metabolite storage rates. While each metabolite likely has its own individual rate, this rate is not reported in literature for many metabolites, therefore a universal, arbitrary number was chosen.

#### Constraints on metabolite flow

As has already been suggested, it is not necessarily logical for metabolites to flow between tissues without suitable constraints on that flow. For instance, water is taken up by roots and transported first to the shoot, then to the leaves and seed tissue (if present). It would not, for instance, make sense for water to travel directly from the root tissue to the seed tissue. Therefore, instead of a single metabolite pool connecting tissue, there are metabolite pools connecting each pair of tissues. These pools are shown in Figs. 3 and 5 as arrows and circles between tissues. The following subsections describe how individual metabolites or groups of metabolites are constrained to logical flow through the system.

#### Water

Mathematically, for the flow of water these logical metabolite links take the following form.

44 $$0\le -{Y}_{root,t}{v}_{root,wate{r}_{in}}\le {v}_{root,wate{r}_{in}}^{bound}$$

45 $${Y}_{root,t}{v}_{root,wate{r}_{out}}=-\,{Y}_{stem,t}{v}_{stem,wate{r}_{in}}$$

46 $${Y}_{stem,t}{v}_{stem,wate{r}_{out}}=-\,({Y}_{leaf,t}{v}_{leaf,wate{r}_{in}}+{Y}_{seed,t}{v}_{,seed,wate{r}_{in}})$$

47 $${v}_{leaf,transpiration}={\tau }_{leaf}\,(when\,light)$$

equation (44) limits the rate of water uptake by the roots to between zero and some pre-defined bound (in the p-ath773 model uptake is defined as a negative flux rate, while output is defined as positive). Equation (45) states that all water output by the root goes to the stem and to no other tissue. Equation (46) ensures in turn that water output by the stem is taken up by either the leaf or seed tissues. The signs in Eqs. (44) through (46) ensure consistency with the sign definition of uptake and output in the p-ath773 model. Equation (47) enforces transpiration from the plant at a certain level calculated from literature sources^40,53,54^. Transpiration is only allowed during the day because it is assumed that the stomas are open during the day (or when light is available), allowing transpiration, and closed at night (or when light is not available). Transpiration is reported as approximately 2.95 mmol water per *m*^2^ per second^54^. This can be converted to 422.3 mmol water per gDW plant per hour based on the information such as the leaf area ratio^40^, which is scaled at each time point as appropriate to give the rate in mmol water per gDW leaf per hour.

#### Micronutrients

Micronutrients, such as nitrates, sulfates, and phosphates, follow much of the same flow pattern through the plant as does water. This is because water transports dissolved micronutrients to the rest of the plant through the xylem. The major differences are: (i) micronutrients will be used up in each tissue so that the amount of each micronutrient leaving each tissue will be less than that entering, which is modeled by Eqs. (48) and (52) below, and (ii) there is no equivalence of transpiration for micronutrients.

48 $$0\le {Y}_{root}{v}_{root,{\kappa }_{in}}\le {v}_{root,{\kappa }_{in}}^{bound}\,\forall \kappa \in {\rm{{\rm K}}}$$

49 $${Y}_{root,t}{v}_{root,{\kappa }_{out}}\le -\,{Y}_{root,t}{v}_{root,{\kappa }_{in}}\,\forall \kappa \in {\rm{{\rm K}}}$$

50 $${Y}_{root,t}{v}_{root,{\kappa }_{out}}=-\,{Y}_{stem,t}{v}_{stem,{\kappa }_{in}}\,\forall \kappa \in {\rm{{\rm K}}}$$

51 $${Y}_{stem,t}{v}_{stem,{\kappa }_{out}}\le -\,{Y}_{stem,t}{v}_{stem,{\kappa }_{in}}\,\forall \kappa \in {\rm{{\rm K}}}$$

52 $${Y}_{stem,t}{v}_{stem,{\kappa }_{out}}=-\,({Y}_{leaf,t}{v}_{leaf,{\kappa }_{in}}+{Y}_{seed,t}{v}_{seed,{\kappa }_{in}})\,\forall \kappa \in {\rm{{\rm K}}}$$

again, signs in the above equations are due to the model convention of denoting uptake of a metabolite as a negative flux, while output of a metabolite is denoted as a positive flux.

#### Sucrose

As is well known, sugars in plants are synthesized in photosynthetic tissue, and are transported to the rest of the tissues through the phloem. In the p-ath773 model, it is assumed that the vast majority of photosynthesis occurs in the leaf tissue, and the photosynthetic output of other tissues is negligible. This assumption is based on two factors: i) leaves are tissues specifically designed to carry out photosynthesis, and ii) photosynthesis relies on above-ground surface area to absorb light to drive the process, and leaves have by far the most surface area. Therefore, the flow of sucrose in the modeled plant system is as being exported by the leaf tissue in Eq. (53), transported through the stem tissue in Eq. (54) (allowing for some use of the sucrose by the tissue), and transported to the seed and root via the stem in Eq. (55). These equations are shown below.

53 $$-{Y}_{stem,t}{v}_{stem,sucros{e}_{in}}={Y}_{leaf,t}{v}_{leaf,sucros{e}_{out}}$$

54 $${Y}_{stem,t}{v}_{stem,sucros{e}_{out}}\le -\,{Y}_{stem,t}{v}_{stem,sucros{e}_{in}}$$

55 $${Y}_{stem,t}{v}_{stem,sucros{e}_{out}}=-\,({Y}_{root,t}{v}_{root,sucros{e}_{in}}+{Y}_{seed,t}{v}_{seed,sucros{e}_{in}})$$

#### Amino acids

The logical flow of amino acids has been defined explicitly via Eqs. (56) through (58) stated below, as having been synthesized in the leaf tissue and exported to seed tissue.

56 $$-{Y}_{stem,t}{v}_{stem,{x}_{in}}={Y}_{leaf,t}{v}_{leaf,{x}_{out}}\,\forall x\in X$$

57 $${Y}_{stem,t}{v}_{stem,{x}_{out}}=-{Y}_{stem,t}{v}_{stem,{x}_{in}}\,\forall x\in X$$

58 $${Y}_{stem,t}{v}_{stem,{x}_{out}}=-{Y}_{seed,t}{v}_{seed,{x}_{in}}\,\forall x\in X$$

this is because seed tissue has not been shown to produce all needed amino acids^29^, and the root and stem models do not require amino acids for biomass production in the defined biomass composition^31^. Essentially, these constraints ensure that all amino acids exported by the leaf are uptaken by the stem, Eq. (56); that these amino acids are not stored in the stem, Eq. (57); and that all amino acids are exported by the stem to the seed tissue, Eq. (58).

#### Oxygen and carbon dioxide

It is well known that photosynthesis produces molecular oxygen and that respiration produces carbon dioxide. Both processes occur in plants, with photosynthesis necessarily dominating when light is available and respiration dominating when light is not available. As such, in this framework it is specified that the p-ath773 model is a net oxygen producer and net carbon dioxide consumer when light is available whereas the p-ath773 model is a net oxygen consumer and net oxygen when light is not available. These restrictions are formulated in the following equations, where Eqs. (59) and (60) deal with conditions when light is available for growth while (61) and (62) apply when no light is available.

When light is available

59 $$-{Y}_{leaf,t}{v}_{leaf,C{O}_{2},in}\ge {Y}_{root,t}{v}_{root,C{O}_{2}out}+{Y}_{seed,t}{v}_{seed,C{O}_{2}out}+{Y}_{stem,t}{v}_{stem,C{O}_{2}out}$$

60 $$-{Y}_{leaf,t}{v}_{leaf,{O}_{2}out}\ge ({Y}_{root,t}{v}_{root,{O}_{2}in}+{Y}_{seed,t}{v}_{seed,{O}_{2}in}+{Y}_{stem,t}{v}_{stem,{O}_{2}in})$$

When light is not available

61 $$0\le {Y}_{leaf,t}{v}_{leaf,C{O}_{2}out}+{Y}_{root,t}{v}_{root,C{O}_{2}out}+{Y}_{seed,t}{v}_{seed,C{O}_{2}out}+{Y}_{stem,t}{v}_{stem,C{O}_{2}out}$$

62 $$0\le -({Y}_{leaf,t}{v}_{leaf,{O}_{2}in}+{Y}_{root,t}{v}_{root,{O}_{2}in}+{Y}_{seed,t}{v}_{seed,{O}_{2}in}+{Y}_{stem,t}{v}_{stem,{O}_{2}in})$$

to enforce these constraints, a parameter, called *π*_*t*_, is defined which takes a value of 1 if light is available for growth and zero otherwise for time *t*. This is incorporated into the model to simplify the above equations into two equations. Note that the “in” and “out” reactions are combined such that if the model is taking up a given metabolite the reaction rate will be negative, while exporting a given reaction would correspond to a positive reaction rate.

63 $$({Y}_{leaf,t}{v}_{leaf,C{O}_{2}}+{Y}_{root,t}{v}_{root,C{O}_{2}}+{Y}_{seed,t}{v}_{seed,C{O}_{2}}+{Y}_{stem,t}{v}_{stem,C{O}_{2}})(1-2{\pi }_{t})\ge 0$$

64 $$({Y}_{leaf,t}{v}_{leaf,{O}_{2}}+{Y}_{root,t}{v}_{root,{O}_{2}}+{Y}_{seed,t}{v}_{seed,{O}_{2}}+{Y}_{stem,t}{v}_{stem,{O}_{2}})(1-2{\pi }_{t})\le 0$$

the (1−2*π*_*t*_) term in the above equations serves as a binary switch alternating between values of −1 and 1 based on the availability of light. In addition to these constraints, some limit must be placed on the uptake of carbon dioxide and oxygen by the leaves of the plant. It has already been noted here the modeled transpiration occurs at 422.3 mmol water per gDW plant per hour^40,52–54^, and this is used as the basis for the exchange of other gasses as well. It is noted that the rate of carbon dioxide uptake is two order of magnitude less than the rate of water loss^52,53^, and an *in vivo* study identifies the rate of carbon dioxide flow into the leaf as 8 *μ*mol/*m*^2^·s^55^, which is converted using the Leaf Area Ratio to 1.14 mmol per gDW plant per hour. Assuming standard atmospheric composition (0.04% Carbon Dioxide and 21% Oxygen), then there are approximately 525 oxygen molecules per carbon dioxide molecule at ground level. Here, the limit of oxygen uptake is proportional (in terms of the composition of the atmosphere) to the limit of carbon dioxide uptake, specifically 598.5 mmol per gDW plant per hour is the oxygen uptake limit used. Further, as plants lack a system to transport gasses from one organ or tissue to another (i.e. a circulatory system in the animal sense) it has been assumed that each tissue is responsible for its own gas exchange. As the leaf is a tissue specifically designed for photosynthesis and gas exchange, it will be assumed that the gas exchange occurring in the leaf is at least one order of magnitude larger than that occurring in the rest of the plant. As the other tissues are modeled as heterotrophic (i.e. not significantly photosynthetic), the rate of oxygen uptake must be limited. Therefore, the limit of oxygen uptake for root, seed, and stem tissues is set at 59.85 mmol per gDW plant per hour.

#### Diurnal carbon storage patterns

Plants store carbohydrates in leaf and stem tissues in the form of starch (leaf and stem) and sucrose (stem) in a pattern where the rates of storage may be modeled by a sine wave with a period of 24 hours^54^. These equations are defined as follows.

65 $${v}_{leaf,starc{h}_{store}}={A}_{l}\,\sin ({f}_{l}(t+{b}_{l}))$$

66 $${v}_{stem,starc{h}_{store}}={A}_{st,1}\,\sin ({f}_{sf1}(t+{b}_{st,1}))$$

67 $${v}_{stem,sucros{e}_{store}}={A}_{st,2}\,\sin ({f}_{sf2}(t+{b}_{st,2}))$$

the calculations for defining the necessary parameters namely *A*_*l*_, *A*_*st*,1_, *A*_*st*,2_, *f*_*l*_, *f*_*st*,1_, *f*_*st*,2_, *b*_*l*_, *b*_*st*,1_, and *b*_*st*,2_ in Eqs. (65) through (67) can be found in Data S1. In summary, the necessary parameters listed above have been fit to experimental data by minimizing the sum of squared error between the Eqs. (65) through (67) using Microsoft Excel’s solver tool.

#### Linking tissue growth rates

We have discovered while building this model that tissue growth rates must have enforced links between growth rates of tissues in the system for two reasons: (i) linking tissue growth rates allows control of the tissue mass ratios so that they may be modeled as they occur in *Arabidopsis* and (ii) this prevents the problem of the model preferentially producing the “cheapest” biomass. The rate of biomass production determined by an SM is the growth rate of the biological system being modeled^8^; therefore, plant mass can be defined as:

68 $${M}_{\theta ,t+\Delta t}\,={M}_{\theta ,t}{e}^{{\mu }_{\theta ,t}t}\,\forall \theta \in \Theta $$

further, the ratio of the masses to two tissues can be defined with reference to a single tissue, such as leaf, in the following manner:

69 $${M}_{\theta ,t}\,=\frac{{x}_{\theta ,t}}{{x}_{leaf,t}}{M}_{leaf,t}\,\forall \theta \in \Theta $$

by having substituted Eq. (69) into Eq. (68) and simplifying the result (see Text S1), linear equations have been written to constrain biomass production rates of root, seed, and stem tissues with respect to leaf tissue as follows:

70 $${\mu }_{\theta ,t}=\,\mathrm{ln}\left(\frac{{x}_{\theta ,t+\Delta t}{M}_{leaf,t}}{{x}_{leaf,t+\Delta t}{M}_{\theta ,t}}\right)+{\mu }_{leaf,t}\,\forall \theta \in \Theta $$

the quantity inside the natural logarithm is already defined for root, seed, and stem tissues as *ω*_*t*_, *η*_*t*_, and *λ*_*t*_, respectively, in Eqs. (34) through (36). Therefore, the following constraints are used in the ORKA framework.

71 $${\mu }_{root,t}=\,\mathrm{ln}({\omega }_{t})+{\mu }_{leaf,t}$$

72 $${v}_{seed,biomass}=\{\begin{array}{ll}0 & {M}_{seed,t}=0,{s}_{t+\Delta t}=0\\ \mathrm{ln}({\eta }_{t})+{\mu }_{leaf,t} & {M}_{seed,t}\ne 0,{s}_{t+\Delta t}\ne 0\\ {\mu }_{leaf,t} & {M}_{seed,t}\ne 0,{s}_{t+\Delta t}=0\\ -{\mu }_{leaf,t} & {M}_{seed,t}=0,{s}_{t+\Delta t}\ne 0\end{array}$$

73 $${\mu }_{stem,t}=\,\mathrm{ln}({\lambda }_{t})+{\mu }_{leaf,t}$$

equation (72) requires further explanation as to why it is not a single function as Eqs. (71) and (73). For the first condition, if there is no seed mass at the initial time point and no seeding level at the next time point (meaning the next time point should also have no seed mass) then there should be no growth of the seed tissue. The second condition is when there is both seed tissue at the current point and at the next time point; therefore, this function is analogous to Eqs. (71) and (73). The final two conditions are artifacts of the exponential nature of the growth rates determined by SMs. The third condition deals with the instance when the seed tissue first appears in the p-ath773 model system. This results in the value of *M*_*seed*,0_ being zero, resulting in the limit of *η*_*t*_ → ∞ as *M*_*seed*,0_ → 0. Similarly, as *η*_*t*_ → ∞ then $\mathrm{ln}({\eta }_{t})\to \infty$. As the model cannot capture infinite growth (and that very high rates of growth would likely result in the auto-cannibalism of existing tissues), we have decided to model the growth in this instance as equal to *μ*_*leaf*,*t*_. while technically not true, this is because it does set an achievable growth rate for the model. Similarly, at the last time point in which seed tissue is part of the system. This results in *x*_*seed*_ = 0. As the fraction of seed mass in the system approaches zero (*x*_*seed*_ → 0), *η*_*t*_ → 0 and as this occurs $\mathrm{ln}({\eta }_{t})\to \,-\infty$. Again, this is obviously an issue since infinite negative growth would be both unrealistic and would result in an infinite ray in the p-ath773 model solution, effectively preventing the solution. Instead, similar to the previous case, growth rate is fixed to the negative rate of the growth of the leaf tissue, −*μ*_*leaf*,*t*_. At this point, it is worthwhile to discuss how seed biomass is lost in a non-productive way (i.e., biomass components are not returned to the metabolic model when seed biomass is lost).

#### Modeling the loss of seed tissue to seed dispersal

One of the most metabolically costly activities for many species, including for *Arabidopsis,* is reproduction. The seed contain a large amount of metabolites which may be metabolized by the embryo to sustain it and allow it to grow before it can photosynthesize. These stored metabolites include fatty acids, proteins, and sugars^56–58^. Further, *Arabidopsis* plants produce a very large number of seeds, on the order of approximately 28,000 seeds per gram dry weight of vegetative mass^34^. To properly model this metabolic investment, the model must ensure that these costly metabolites from the seeds are not returned to the plant metabolism when seed biomass is lost in seed dispersal. To explain how this could happen, generally the biomass reaction consumes metabolites such as amino acids, fatty acids, sugars, and other necessary compounds in its production and in these cases, *μ*_*seed*,*t*_ > 0. Conversely, when seed mass is being lost from the system, *μ*_*seed*,*t*_ < 0, the biomass precursors are produced from the biomass pseudo-metabolite, and without careful constraints, this loss of seed biomass could cause these precursor metabolites which constitute biomass to remobilize (e.g. used in the metabolism for metabolite production elsewhere) into the metabolic model, resulting in the use of these resources which should be lost to the plant. Essentially, this would simulate a plant consuming the stores of metabolites in its own seeds, rather than releasing those seeds with its stores intact. Instead, to allow modeling of the complete and non-productive loss of seed biomass, an extra equation called “biomass loss” has been defined to be identical to the biomass equation except it does not produce the biomass pseudo-metabolite. This allows the definition of the following constraint which is in effect during the silique ripening growth stage.

74 $${\mu }_{seed,t}=-{v}_{biomassloss,t}$$

this ensures that lost biomass is not re-introduced into the plant metabolism but that it is modeled as lost.

#### Defining the usage of seed stores by the seedling

For the earliest stages of *Arabidopsis* growth, here named as seed germination stage and seed germination to leaf development transition, a seedling’s primary source of carbon is its reserves of stored carbohydrates, proteins, and lipids. It has been shown that seeds have stores of approximately 0.425 *μ*g of sucrose, 6 *μ*g of fatty acids, and 6 *μ*g of proteins (modeled here as component amino acids) available^30^. As no information concerning the pattern of usage of the seed storage has been found, it has been assumed that the stores are utilized at a constant rate during the duration of the seed germination period and that all the storage is fully consumed by the end of the seed germination to leaf development transition stage, which has been defined the point at which the cotyledons are fully open and leaf development intensifies^32^. The rate at which the seedling should uptake the seed storage has been determined by identifying the moles (mmol) of each major component of the seed storage and dividing by the time over which the seedling consumes those. This has resulted in a mmol · h^−1^ quantity. See Data S1 for this calculation. This quantity has then been scaled by plant mass to result in a mmol·gDW^−1^ · h^−1^ quantity, which is used to bound the uptake rates of stored metabolites in the seed. As the leaf has proven to be the most metabolically active tissue, it is assumed that the leaf tissue of an *Arabidopsis* seedling uptakes the stored fatty acids, amino acids, and carbohydrates that are provided for seedling growth during the seed germination stage when the leaves have no access to light (see Fig. 5, Seed Germination).

#### Defining initial plant and tissue ratios

As the model advances plant and tissue masses with respect to time, the establishment of initial mass for plant and tissues has become important in this framework. Experimental evidence has shown that *Arabidopsis* seeds have a fresh weight (FW) of 25.3 *μ*g and have only about 7% water content^30^. The embryo itself is assumed equal to the seed mass less the mass of seed stores of sucrose (0.425 *μ*g), Fatty Acids (6 *μ*g), and proteins (6 *μ*g)^30^. Having assumed that the dry matter content ratio holds for the embryo as well, this has left approximately 11.0 *μ*g dry weight (DW) for the embryo. As information on the ratio of tissue masses in *Arabidopsis* has not been documented in literature, the general ratio for herbaceous plants has been used as a starting point, namely 0.46:0.24:0.3 leaf:root:stem FW^57,59^. This ratio has been converted to DW ratio for stoichiometric modeling. Experimental data has shown that the dry matter content of leaf tissue is 0.212 DW/FW, of root tissue is 0.170 DW/FW, and of the stem tissue is 0.176 DW/FW^57^. Having converted the FW ratios to DW ratios has given the ratio of 0.511:0.267:0.211 leaf:root:stem DW. While the dry matter content of an embryonic *Arabidopsis* is much higher than that of a mature plant (the source of the utilized dry matter content ratios), this DW tissue ratio has non-the-less been assumed to be accurate for the embryo due to lack of evidence to the contrary.

#### Defining stage times

Time points which define the transition between different stages of growth have been taken from a single source of experimental evidence^32^. Stage transitions selected include the transition to stage 0.70 (Seed Germination to Leaf Development transition in Fig. 5), stage 6.00 (Leaf Development to Flower Production transition in Fig. 5), and stage 8.00 (Flower Production to Silique Ripening transition in Fig. 5). Not all lifecycle stage transitions for which there is experimental evidence have been incorporated into this model. In some cases, this has been due to a lack of metabolic relevance, such as the transition from stage 1.04 to stage 1.05 where the plant transitions from 4 rosette leaves to 5 rosette leaves that are greater than 1mm in length. This has not been important to the p-ath773 model as a ratio of plant mass to leaf surface area ratio is used instead^34^ (see Data S1). Others that cannot be modeled by the current framework include tissues such as stage 5.10 which is when the first flower bud is visible^32^, as the current p-ath773 model has no flower bud tissue. The length of the seed ripening stage has also been determined by experimental evidence^34^.

#### Defining the change in tissue mass ratios with growth stage

Using available literature evidence, two endpoints for the plant tissue mass ratios have been defined when no seeds are present and all seeds are produced^32^. The transition between these states are assumed to be linear with respect to a parameter called seeding, defined above as *s*. These relationships are then modeled as:

75 $${x}_{leaf,t}={c}_{leaf}\,\ast \,{s}_{t}+{x}_{leaf,0}$$

76 $${x}_{root,t}={c}_{root}\,\ast \,{s}_{t}+{x}_{root,0}$$

77 $${x}_{seed,t}={c}_{seed}\,\ast \,{s}_{t}+{x}_{seed,0}$$

78 $${x}_{stem,t}={c}_{stem}\,\ast \,{s}_{t}+{x}_{stem,0}$$

$${c}_{leaf}=-0.2514;{c}_{root}=-\,0.02862;{c}_{seed}=0.2030;{c}_{stem}=0.07698$$

$${x}_{leaf,0}=0.511;\,{x}_{root,0}=0.267;\,{x}_{seed,0}=0;\,{x}_{stem,0}=0.211$$

where *x*_*tissue*_ has been defined as the tissue mass fraction with respect to the total mass of the plant, *c*_*tissue*_ is defined as the change in tissue mass fraction with respect to seeding, and *x*_*tissue*_ is defined as the initial mass fraction of each tissue. The gain in the seeding parameter has been assumed to be linear with time and is fit to experimental time point describing the fraction of flowers produced^32^ (see Data S1 and Text S1).

#### Defining the availability of light

The amount of light available to the model to use for photosynthesis has been defined initially by literature sources used for other constraints^56^, and scaled by the transmittance of that light source (fluorescent lights)^36^ and the absorbance of *Arabidopsis* leaves^36^ and surface area to plant mass of *Arabidopsis leaves*^40^. This has been approximately estimated to be 4.00 mmol·gDW plant^−1^·h^−1^. This value has been shown to be 21.50% of the total photons output by the fluorescent light (see Data S1 and Text S1).

#### Defining model maintenance and senescence costs

An important consideration in any SM is the definition of a maintenance cost, which is typically defined as ATP hydrolysis^7^. Biomass-based maintenance and senescence costs have been defined as they have been suggested as more accurate or applicable for plant systems^38,39^, but have not yet been used in an SM. We have defined maintenance and senescence costs as a biomass drain on each tissue scaled by tissue mass in Eq. (30). A maintenance cost value of k_m_ = 0.03 day^−1^ has been defined which is in an order of magnitude typical for plant systems^60^, and the same value has been defined for plant senescence, k_s_, as this parameter appears to be generally of the same order of magnitude^38,39^. These rates are then converted into their per hour equivalent and scaled by tissue mass to enforce these constraints. Only a single constraint has been defined for both phenomena as both are biomass drains whose effect is additive. Literature evidence, including pictorial evidence of plant phenotype at various growth stages, appears to suggest that the rate of plant senescence increases drastically as the flowering production stage finishes and the silique ripening phases begin (in literature, growth stage 0.65 to 9.70)^32^. Further, it appears that the plant no longer maintains current mass, but allows tissues to die and desiccate^32^. This has been included in the p-ath773 model in that plant senescence is increased by an order of magnitude and plant maintenance is set to zero following the end of the Flower Production stage.

#### Defining model objective functions

For all analyses and results, the objective function of p-ath773 has been to maximize the sum of the biomass production rates for all four tissues according to the following equation (referred to as the default objective).

79 $$maximize\,z={v}_{growth,leaf}+{v}_{growth,root}+{v}_{growth,seed}+{v}_{growth,stem}$$

where *z* has been defined as the objective variable with *v*_*growth,tissue*_ being defined as the rate of biomass production, in units of h^−1^, of the tissue referenced. The maximization of this objective function is approximately equivalent to maximizing the growth rate (change in mass per unit time) of the plant as a whole. This objective function, in early model iterations, has led to one major issue, namely how to avoid the model producing only the metabolically “cheapest” tissue which could result in the maximum objective value but is biologically unrealistic. This is addressed by Eqs. (23) through (28) and will be further discussed later in the methods section.

It has been noted that the maximization of plant biomass has not been the only feasible objective function for plant SM system; for instance, one alternate objective function is the maximization of plant photonic efficiency^13,22^. This objective has generally been framed as minimizing the amount of light used by the plant system, given a required growth rate^13,22^. As the purp authors gratefully acknowledge funding ose of this paper is to showcase the ORKA method, rather than the p-ath773 model, alternative objective functions have not been implemented but are possible to implement.

Flux Variability Analysis (FVA) has also been performed on the p-ath773 model which uses all previously defined constraints and the previously defined ORKA method. All flux bounds and constraints are the same and the FVA has an objective function defined as follows:

80 $$maximize\,or\,minimize\,z={\sigma }_{j}{v}_{j}$$

where the FVA model solution has been iterated for each reaction *j*, and *σ*_*j*_ has been valued at 1 for the current reaction whose maximum and minimum are to be investigated and 0 for all others and is stepped through first maximizing and then minimizing each reaction. Due to restrictions of the time allowed for model solutions, nine points has been selected at which to perform FVA. These points are 1 hour after germination (HAG, seed germination stage, dark), 70 HAG (seed germination to leaf development transition, light), 90 HAG (seed germination to leaf development transition, dark), 177 HAG (leaf development stage, light), 181 HAG (leaf development stage, light), 770 HAG (flower production stage, light), 810 HAG (flower production stage, dark), 1155 HAG (flower production to silique ripening transition, light), 1170 HAG (flower production to silique ripening transition, dark), 1190 HAG (silique ripening stage, dark), 1199 HAG (silique ripening stage, light). These results generally showed narrow ranges for allowable flux rates.

### Software platforms used

Some of the files used in this work and included in the GitHub repository are files of programming codes, and all code is not written in the same programming language. Text S1 lists which programming language each file which is a code utilizes. For Python code, version 3.3 is used; for Perl, both version 5.26 (unix) and Strawberry Perl version 5.24.0.1 (windows) were used; GAMS code utilizes version 24.7.4. Some codes included in GitHub p-ath773 repository (10.5281/zenodo.3735103) for this work have been run using the Holland Computing Center at the University of Nebraska, Lincoln. In addition, portions of the GitHub p-ath773 repository utilize the module the LWP (the world-wide web library for Perl) version 6.39 and have been run on a Windows desktop computer.

## Supplementary information

Supplementary Text S1

Supplementary Dataset 1

Supplementary Dataset 2

## Data Availability

The authors declare that the code supporting the findings of this study are available in the p-ath773 GitHub repository (10.5281/zenodo.3735103).
