# Supplementary material for: Introducing an Optimization- and explicit Runge-Kutta- based Approach to Perform Dynamic Flux Balance Analysis
Source: Sci Rep. 2020 Jun 8;10:9241. doi: 10.1038/s41598-020-65457-4 (PMC7280247; doi:10.1038/s41598-020-65457-4)
Supplement: Supplementary file 1 — Supplementary Text S1 [file 41598_2020_65457_MOESM1_ESM.pdf]

Introducing an Optimization- and explicit Runge-Kutta- based Approach to Perform Dynamic Flux Balance Analysis

Supplemental Files

Authors: Wheaton L. Schroeder and Rajib Saha

Contents

Supplemental materials legends ... 3

Symbols used ... 4

- Sets ... 4
- Variables ... 4
- Parameters ... 4
- Functions ... 5

Abbreviations used ... 7

Definition of biomass composition ... 8

- With respect to seed biomass ... 8
- With respect to leaf biomass ... 8

Reaction confidence scoring. ... 9

Enforcing the logical flow of metabolites between tissues. ... 10

Defining the diurnal patterns of carbohydrate storage. ... 12

Defining the change in tissue mass ratios with growth stage. ... 13

Defining the availability of light. ... 14

Defining the mass step between time points. ... 15

Supplemental materials legends

Text_S1.pdf: A Microsoft Word file designed to help navigate the files, code, and data provided via GitHub (DOI: 10.5281/zenodo.3735103) that will be used to as well as to outline the general file structure and workflow used with the p-ath773 model to make model implementation easier. This GitHub repository allows this work to be reproduced. This file also contains lists of symbols and abbreviations used. This file also contains supplemental methods including detailed descriptions of how the biomass compositions, confidence scores, diurnal patterns of metabolite storage, light availability, and the whole-plant mass step were determined.

Data_S1.xlsx: This file is a Microsoft Excel file which store a wide variety of information concerning the p-ath773 model. This include the manually-curated GPR results for each tissue model, the calculations pertaining to the determination of the biomass equation for each tissue model, calculations for various parameters used in the p-ath773 model to incorporate literature data, and calculations pertaining to the diurnal storage and uptake of carbohydrates.

Data_S2.xlsx: This file is a Microsoft Excel file which store the reaction rate for all reactions at all time points, the concentration for all metabolites at all time points, and graphs related Figs. 7, 8, and 9.

Symbols used

Sets

I: Set of metabolites in a given model, individual elements are indicated by i.
X: set of amino acids which are synthesized by the leaf and exported to other tissues. $X \subset I$
I': Set of metabolites for which concentration is tracked. $I' \subset I$
U: set of micronutrients which the root uptakes from the soil individual elements are u. U $\subset I$
J: Set of reactions in a given model, individual elements are indicated by j.
Θ: Set of tissues in the model
T: set of time points over which the model is solved, individual elements are indicated by t.
N: Runge-Kutta steps of the chosen or generic Runge-Kutta method, with elements denoted by n. The final step is denoted n_f, therefore $N = [n_0, n_f]$.
a: Index for a generic set over which a summation is performed.
K: Set of micronutrients uptaken by the roots, with elements denoted by κ. K $\subset I$.

Variables

$v_{\theta,biomass,t} \equiv \mu_{\theta,t}$: Rate of biomass production in tissue θ at time t.
$v_{\theta,reaction,t}$: Rate of reaction j in tissue θ at time t.
$z_{i,t}$: Concentration of metabolite i at time t.
Y_t: Biomass concentration at time t. This is used both in the general formulation of SOA and ORKA, as well as to indicate the overall plant biomass.
$Y_{\theta,t}$: Biomass concentration of tissue θ at time t.

Parameters

Parameters are symbols which have a constant value, or a value which can be calculated from other constant values and variable values which have already been solved, at each time point, but may change value between time points.
Δt: Size of time step taken by the given DFBA method.
$v_{j,t}^{LB}$: Lower bound of rate of reaction j at timepoint t.
$v_{j,t}^{UB}$: Upper bound of rate of reaction j at timepoint t.
S_{ij}: Stoichiometric coefficient of metabolite i in reaction j.
a: Generic number around which a Taylor series expansion is made.
C_1: Generic parameter of undefined value which appears in intermediate steps for solving ODEs.
$\Gamma_{j,t}$: Multiple application Trapazoid rule-based integral estimate of the integral of $v_{j,t}$ from the first to the second time point.
b_n: Parameter associated with the generic Runge-Kutta of the nth order. These parameters are used to combine Runge-Kutta step size estimates to get the final step size estimate.
k_n: n^{th} step size estimate of the dependent variable made by the Runge-Kutta method.
c_n: Step size of the independent variable in the Runge-Kutta method used. Largest index of c_n is $c_{n_{max}}$.
a_{nm}: Parameters associated with generic Runge-Kutta methods which is used to make sub-steps of the independent variable for estimates of k_n.

$x_{\theta,t}$: Mass fraction of the total plant which is accounted for by tissue θ at time t.
$x_{\theta,0}$: Mass fraction of the total plant which is accounted for by tissue θ at time 0 (initial condition).
c_{θ}: The rate of change in tissue θ mass fraction with respect to seeding. Used to have a linear biomass fraction 'slider' based on the maturity of the plant.
s_t: Level of seeding at time t. This parameter is used to indicate plant maturity and to simulate the increase in seed tissue mass fraction (and corresponding decrease of other tissues) as time passes.
ξ_t: Parameter used to split the calculation of $\frac{dY_t}{dt}$ into multiple equations to make the formulation more readable.
ω_t: Parameter used to split the calculation of $\frac{dY_t}{dt}$ into multiple equations to make the formulation more readable. Deals with the change in plant mass fraction that is root tissue.
η_t: Parameter used to split the calculation of $\frac{dY_t}{dt}$ into multiple equations to make the formulation more readable. Deals with the change in plant mass fraction that is seed tissue.
λ_t: Parameter used to split the calculation of $\frac{dY_t}{dt}$ into multiple equations to make the formulation more readable. Deals with the change in plant mass fraction that is stem tissue.
ζ_t: Parameter used to split the calculation of $\frac{dY_t}{dt}$ into multiple equations to make the formulation more readable. Deals with the change in plant mass fraction that is stem tissue.
ρ_t: Parameter used to split the calculation of $\frac{dY_t}{dt}$ into multiple equations to make the formulation more readable. Deals with the change in plant mass fraction that is stem tissue.
δ_t: Parameter used to split the calculation of $\frac{dY_t}{dt}$ into multiple equations to make the formulation more readable. Deals with the change in plant mass fraction that is stem tissue.
ϕ: Generic time-dependent parameter, used to show an equation that applies to a number of parameters.
τ_{leaf}: Transpiration rate of water from the leaf when the stomata are open during the day. Calculated from literature.
$v_{root,water_{in}}^{bound}$: Limit on rate at which the root tissue can take up water.
$v_{root,\kappa_{in}}^{bound}$: Limit on the rate at which the root tissue can take up micronutrients
A_l: Amplitude of diurnal starch storage pattern in leaf.
f_l: Frequency of diurnal starch storage pattern in leaf.
b_l: X-intercept of diurnal starch storage pattern in leaf.
$A_{st,1}$: Amplitude of diurnal starch storage pattern in stem.
$f_{st,1}$: Frequency of diurnal starch storage pattern in stem.
$b_{st,1}$: X-intercept of diurnal starch storage pattern in stem.
$A_{st,2}$: Amplitude of diurnal sucrose storage pattern in stem.
$f_{st,2}$: Frequency of diurnal sucrose storage pattern in stem.
$b_{st,2}$: X-intercept of diurnal sucrose storage pattern in stem.
π_t: Binary parameter whose value states whether or not a

Functions

$f(t)$: Generic function dependent on t.
$f(t, Y_t)$: Generic function dependent on t and Y_t.

$f^{(n)}(t)$: n[th] derivative of generic function $f(t)$.
$g(t)$: Function estimated from $f(t)$ using a Taylor series expansion.
$O(h^n)$: Big O notation used to indicate the order of error for an estimated function, where h is the variable by which the error is defined an n is the order of that error.

Abbreviations used

For the convenience of our readers, a list of abbreviations used is given below.
GPR: Gene-Protein-Reaction
SM: Stoichiometric Model
GSM: Genome-Scale Model
GEM: GEnome-scale Model
FBA: Flux Balance Analysis
dFBA: dynamic Flux Balance Analysis
FVA: Flux Variability Analysis
LP: Linear Problem
Arabidopsis: *Arabidopsis thaliana*
LHS: Left-Hand Side
RHS: Right-Hand Side
wrt: with respect to
gDW: grams Dry Weight
DW: Dry Weight
gFW: grams Fresh Weight
FW: Fresh Weight
MFA: Metabolic Flux Analysis
KEGG: Kyoto Encyclopedia of Genes and Genomes
DAG: Days After Germination
HAG: Hours After Germination
COBRA: COnstraint-Based Reconstruction and Analysis
SOA: Static Optimization-based dFBA Approach
ORKA: Optimization- and explicit Runge-Kutta dFBA Approach
HAG: Hours After Germination

Definition of biomass composition

With respect to seed biomass

The MFA study referenced had identified the biomass amino acid composition with respect to 14 amino acids (others destroyed during hydrolysis) and used proteinogenic statistical analysis to identify the relative abundance of the other amino acids in biomass. The biomass composition with respect to lipids and carbohydrates was also determined in this MFA, but no integrated biomass composition (comparing relative abundance of individual amino acids, lipids, and carbohydrates in a single analysis) was provided [26]. Therefore, the MFA-determined rates of fatty acids, amino acids, and carbohydrates being added to plant biomass as sink reactions have been used as a basis of comparison to determine the relative amounts of each type of molecule in seed biomass. See Supplemental File 5 for these calculations.

With respect to leaf biomass

In the SM study used to define leaf biomass composition [18], histidine has been added to the biomass equation as it was not present, and certain compounds comprising a very small mass fraction of biomass, such as coniferyl alcohol and hexadecanoic acid, have not been included so as to reduce model size without unduly sacrificing accuracy of biomass composition. See Supplemental File 5 for how leaf biomass was determined.

Reaction confidence scoring.

Reactions with score of zero either have been user-defined reactions with no direct physical equivalent, such as the biomass reaction, which are nonetheless essential in stoichiometric modeling or are artificial and blocked reactions that have been defined in order to help organize the model file by KEGG-defined pathways to make manual editing easier. Reactions with a score of one have been determined as reactions necessary for modeling, but for which we have provided no literature-based evidence for their inclusion. These reactions are typically non-enzymatic, or transport/exchange reactions required for biomass production. Reaction confidence scores of two, three, or four indicate that evidence exist for its inclusion in the model. For most reactions, genomic evidence (score of two) has been considered sufficient for their inclusion. Genomic evidence has been automatically determined using Supplemental File 18, which automates the establishment of GPR links using KEGG. This is typically done by identifying proteins by their Enzyme Classification (EC) numbers associated with each reaction, then identifying arabidopsis genes which can produce those enzymes. Some reactions without KEGG identifiers have had their GPR defined in the same manner, but manually as opposed to in an automated workflow. This has included oxidative phosphorylation and photosynthesis. For reactions without defined GPR links, knock-in/knock-out type studies (score of three) were not been consulted. This is because direct literature evidence (score of four) is preferable and can be found for much of core-carbon metabolism as *Arabidopsis thaliana* is a model plant species.

Enforcing the logical flow of metabolites between tissues.

It has been well known that each tissue in a plant system is capable of gas exchange. It has also been well known that root tissue is generally non-photosynthetic and we have assumed that the carbon fixation (photosynthesis) of the stem and seed tissues are negligible compared to that of the leaves. This is because we have assumed that photosynthetic rate is a function of surface area, and it is well known that the surface area of the stem and seed tissues are much less than that of the leaf tissue. Therefore, root, stem, and seed tissues have been constrained such that they uptake oxygen and export carbon dioxide across the system boundary (dashed line in the system diagrams of Fig. 1). The leaf tissue has been constrained such that the carbon fixation rate when light is available is greater than the rate at which carbon dioxide is exported by the other tissue so that the net effect of the entire plant system is carbon fixation. In all tissue interaction constraints, including that for carbon dioxide and those described later in this sub-section, exchange flux rates (units of mmol/gDW·h) have been scaled by the mass of each tissue (gDW) to ensure mole balance (all fluxes having units of mmol/h) in these constraints. It has been well known that the roots of a plant uptake micronutrients (such as nitrogen, phosphorous, and sulfur sources), ions (such as protons), and water for use by the rest of the plant system. Similarly, it has been well known that the stem tissue transports these nutrients to other plant tissue. Therefore, in p-ath780, the root tissue has uptaken water, phosphate, ammonia, sulfate, and protons across the system boundary. Tissue interaction constraints are then defined such that the amounts of water, phosphate, ammonia, sulfate, and protons stem has uptaken is less than the amount uptaken by the root. Similarly, the amounts of water, phosphate, ammonia, sulfate, and protons uptaken by leaf and seed tissue combined have been constrained to be less than or equal to that exported by the stem. In addition, it has been well known that during the daylight hours, plants lose water through the process of transpiration. In p-ath780, transpiration is modeled as the export of water from the leaf to outside the system boundary. Transpiration for arabidopsis is measured as 0.2 mmol $H_2O{\cdot}m^{-2}{\cdot}s^{-1}$ [29]. Scaling this by the leaf surface area ratio of arabidopsis, 0.0398 m^2 leaf surface area per gDW leaf biomass [28], has given a transpiration rate of 28.6315 mmol/gDW·h with respect to leaf mass.

Amino acid interactions between tissues have been defined out of modeling necessity. From a Metabolic Flux Analysis (MFA) of arabidopsis seed tissue, 14 amino acids have been identified and their rates of production from pathways inherent to the seed tissue were identified [26]. In truth, 16 amino acids had been measured, but due to the methods used by the study, aspartic acid and asparagine were indistinguishable (listed as one amino acid Asx), as were glutamic acid and glutamine (listed as Glx) [26]. As seed biomass has been determined to be composed of all 20 amino acids [26] and we have found no evidence for the synthesis of the remaining amino acids in the seed tissue, we have assumed that the remaining amino acids were synthesized in the leaf tissue and transported to the seed tissue. A total of 14 amino acids have been allowed to be transported between leaf and seed tissue via the stem tissue, including the four amino acids not produced by the seed tissue: arginine, cysteine, methionine, and tryptophan, and ten other amino acids which the seed tissue model cannot synthesize at a rate equal to its needs for growth or were otherwise found necessary for plant system growth were allowed to be transported between tissues. These ten extra amino acids are: glutamate, glycine, alanine, lysine, aspartate, glutamine, serine, proline, valine, and threonine. All amino acids use the same set of three constraints. The net effect of these constraints has been that the amount of any amino acid exported by the leaf tissue is equal to the amount uptaken by the seed tissue, but that the metabolites are required to first pass through the extracellular environment of the stem tissue (e.g. pass through some vascular tissue).

It has been well known that sugars synthesized in the leaves are transported to non-photosynthetic tissues via plant vascular tissue. In p-ath780, this has been modeled by three constraints which ensure that: i) the amount of sucrose uptaken by the stem is equal to the amount exported by the leaf, ii) the amount of sucrose exported by the stem is less than or equal to that imported by the stem, iii) the amount of sucrose uptaken by the root and seed tissues combined is equal to the amount of sucrose exported by the stem tissue.

Defining the diurnal patterns of carbohydrate storage.

It has been experimentally shown that plant tissues store sugars during periods of growth where light is available, and consume those store when light is no longer available [32]. Experimental data concerning the extent to which this occurs over a 12 hours light and 12 hours dark diurnal metabolic cycle has shown that the peak storage of starch in the leaf tissue is approximately 32.8 μmol hexose/gFW, while the trough storage is approximately 3.6 μmol hexose/gFW on a 24-hour period [32]. The slope of this curve has been used to define the rate at which starch is uptaken or stored (see Supplemental File 5). This storage/uptake cycle has been fit to a sine wave with a 24-hour period (frequency 0.261799 h^{-1}) with an amplitude of $9.44\cdot10^{-3}$ mmol starch dimer/gDW·h and an x-intercept of 0.0726 hours (indicating the plant takes about 4.3 minutes to switch between storage and usage modes). Similarly, it has been demonstrated that the shoot (stem) tissue of arabidopsis likewise stores and uptakes starch and sucrose [37] in a manner which can be similarly fit to a sine curve to describe rate. These sine curve amplitudes have been determined to be $1.66\cdot10^{-2}$ mmol/gDW·h and $1.25\cdot10^{-3}$ mmol/gDW·h of starch dimer and sucrose, respectively. Both curves have a 24-hour period and an x-intercept of zero. In p-ath780, these sine curves form constraints which have required uptake or storage of starch and sucrose at the appropriate points in the diurnal cycle in all stages where photosynthesis occurs (e.g. all except Seed Germination). In the exceptional case of Seed Germination, internal starch storage and uptake has been constrained to zero.

Defining the change in tissue mass ratios with growth stage.

It has been assumed that the ratios between leaf, root, and stem tissues are consistent with that of a generic herbaceous plant, 0.511:0.267:0.211 leaf:root:stem DW [31], for all growth states. Combining this with an initial seed tissue mass of 0, this forms one end point for tissue mass ratio change modeling for when *s=0*. For stages which have seed mass (Flower Production, Flower Production to Silique Ripening Transition, and Silique Ripening) a parameter, called seeding, defined as *s* above, has been defined which indicates the fraction of maximum seed mass that is currently a part of the plant system. In determining the rate of tissue mass fraction change, the leaf, seed, and stem harvest masses have been noted as 163.7 mg DW, 127.9 mg DW, and 188 mg DW, respectively [24], and this is used as the other end point for tissue mass ratio change when *s=1*. When assuming that the ratio of 0.267:0.211 root:stem tissue holds, when all seeds are produced and part of the plant system, the tissue mass ratios are 0.2598:0.2387:0.2030:0.2984 leaf:root:seed:stem DW. It is assumed that the transition between these states is linear wrt the seeding parameter which can be used to update the tissue mass ratios. These relationships of tissue mass fraction of entire plant DW are defined as follows:

$$x_{leaf} = c_{leaf} * seeding + x_{leaf,0} \quad (36)$$

$$x_{root} = c_{root} * seeding + x_{root,0} \quad (37)$$

$$x_{seed} = c_{seed} * seeding + x_{seed,0} \quad (38)$$

$$x_{stem} = c_{stem} * seeding + x_{stem,0} \quad (39)$$

$$c_{leaf} = -0.2514; c_{root} = -0.02862; c_{seed} = 0.2030; c_{stem} = 0.07698$$

$$x_{leaf,0} = 0.511;\ x_{root,0} = 0.267;\ x_{seed,0} = 0;\ x_{stem,0} = 0.211$$

Where x_{tissue} has been defined as the tissue mass fraction with respect to the total mass of the plant, c_{tissue} is defined as the change in tissue mass fraction with respect to seeding, and x_{tissue} is defined as the initial mass fraction of each tissue. The gain in the seeding parameter has been assumed to be linear with time, and is fit to experimental time point describing the fraction of flowers produced [24], see Supplemental File 5 for calculations. Due to the lack of data suitable for modeling purposes and the lack of a flower model in p-ath780, it is assumed that flower tissue is equivalent to seed tissue, and it has been assumed that the fraction of flowers open can be equated to the seeding parameter. For example, when 10% of total flowers have opened at time 35.9 days after germinating (DAG) then seeding has a value of 0.1. Using a linear regression fit, we have determined that the seeding parameter gains 0.00237 per hour during the Flower Production stage. Similarly, as the first seeds are lost at 48 DAG and all seeds have been loosed at 61 DAG, a linear regression has resulted in a seeding parameter loss of -0.00359 per hour in the Silique Ripening stage (Figure 1). The overlap between these two stages has used a per hour seeding parameter change equal to the sum of the change in the Flower Production and Silique Ripening stages (-0.00109 per hour) (Flower Production to Silique Ripening Transition, Figure 1).

Defining the availability of light.

According to a source which we have used for other experimental data which informs p-ath780, arabidopsis plants have grown under 12 hour light and 12 hour dark diurnal cycles under 130 $\mu mol/m^2 \cdot s$ intensity fluorescent light [32]. Fluorescent light has thin transmittance peaks at 544 and 609 nm [33], whereas peak absorption for arabidopsis leaves are 440 and 680 nm [34]; therefore, the light which has been provided to the plant for growth does not perfectly match that which can be used by the plant for photosynthesis. By having assumed that all light absorbed by the leaf is usable in photosynthesis (either photosystem), and calculating the number of available photons for the photosystem, it has been determined that the availability of photons for photosystem use is 100.6 $mmol/m^2 \cdot s$. This has been converted to a usable form for modeling by defining the availability of photons in terms of plant dry weight. Using an average value for leaf surface area per mg DW plant [34], the availability of photons for the photosystem is 4.00 mmol/gDW plant·h. This value has been shown to be 21.50% of the total photons output by the fluorescent light. See Supplemental File 5 for details on this calculation.

Defining the mass step between time points.

Generally, if we could assume a constant exponential growth rate the differential equation for exponential growth can be defined as:

$$\frac{dM}{dt} = v_{growth}M$$

Problem: v_{growth} is also a function of time and plant mass, so this differential equation is not strictly true. We know that exponential growth can be described as:

$$M = M_0 e^{v_{growth}*t}$$

Taking the time derivative:

$$\frac{dM}{dt} = M_0 * \frac{d}{dt}\left(e^{v_{growth}*t}\right)$$

Using the chain rule:

$$\frac{dM}{dt} = M_0 * \frac{d}{du}(e^u) * \frac{du}{dt}$$

$$u = v_{growth} * t$$

$$\frac{du}{dt} = \frac{d}{dt}\left(v_{growth} * t\right) = v_{growth} * \frac{d}{dt}(t) + t * \frac{d}{dt}\left(v_{growth}\right) = v_{growth} + t * \frac{d}{dt}\left(v_{growth}\right)$$

Where the derivative of u was found using the product rule. Substituting and solving we get:

$$\frac{dM}{dt} = M_0 * \frac{d}{du}(e^u) * \left(v_{growth} + t * \frac{d}{dt}\left(v_{growth}\right)\right)$$

$$\frac{dM}{dt} = M_0 * e^u * \left(v_{growth} + t * \frac{d}{dt}\left(v_{growth}\right)\right)$$

$$\frac{dM}{dt} = M_0 * e^{v_{growth}*t} * \left(v_{growth} + t * \frac{d}{dt}\left(v_{growth}\right)\right)$$

Some numerical approximation of $\frac{dv_{growth}}{dt}$ will need to be made. However, we do not know the total growth rate, instead we know the growth rate of individual tissues, therefore more accurately we have:

$$M_{plant} = M_{leaf,0}e^{v_{leaf,bio}*t} + M_{root,0}e^{v_{root,bio}*t} + M_{seed,0}e^{v_{seed,bio}*t} + M_{stem,0}e^{v_{stem,bio}*t}$$

Generalizing the differential equation findings for the above equation gives:

$$\frac{dM_{plant}}{dt} = \sum_{tissues}\left[M_{tissue,0} * e^{v_{tissue,bio}*t} * \left(v_{tissue,bio} + t * \frac{d}{dt}\left(v_{tissue,bio}\right)\right)\right]$$

This might be simplified by acknowledging that the time step should be equal to one, so:

$$\boldsymbol{\frac{dM_{plant}}{dt} = \sum_{tissues}\left[M_{tissue,0} * e^{v_{tissue,bio}} * \left(v_{tissue,bio} + \frac{d}{dt}\left(v_{tissue,bio}\right)\right)\right]}$$

Expanded this yields:

$$\frac{dM_{plant}}{dt} = M_{leaf,0} * e^{v_{leaf,bio}} * \left(v_{leaf,bio} + \frac{d}{dt}\left(v_{leaf,bio}\right)\right) + M_{root,0} * e^{v_{root,bio}} * \left(v_{root,bio} + \frac{d}{dt}\left(v_{root,bio}\right)\right) + M_{seed,0} * e^{v_{seed,bio}} * \left(v_{seed,bio} + \frac{d}{dt}\left(v_{seed,bio}\right)\right) + M_{stem,0} * e^{v_{stem,bio}} * \left(v_{stem,bio} + \frac{d}{dt}\left(v_{stem,bio}\right)\right)$$

This can be redefined using the following relations between each tissues growth rate and that of the leaf growth rate. These are the relations:

$$v_{root,bio} = \ln\left(\frac{x_{root}M_{leaf,0}}{x_{leaf}M_{root,0}}\right) + v_{leaf,bio}$$
$$v_{seed,bio} = ln\left(\frac{x_{seed}M_{leaf,0}}{x_{leaf}M_{seed,0}}\right) + v_{leaf,bio}$$
$$v_{stem,bio} = \ln\left(\frac{x_{stem}M_{leaf},0}{x_{leaf}M_{stem,0}}\right) + v_{leaf,bio}$$

Making these substitutions yields:

$$\frac{dM_{plant}}{dt} = M_{leaf,0} * e^{v_{leaf,bio}} * \left(v_{leaf,bio} + \frac{d}{dt}\left(v_{leaf,bio}\right)\right) + M_{root,0} * e^{\ln\left(\frac{x_{root}M_{leaf,0}}{x_{leaf}M_{root,0}}\right) + v_{leaf,bio}} * \left(\ln\left(\frac{x_{root}M_{leaf,0}}{x_{leaf}M_{root,0}}\right) + v_{leaf,bio} + \frac{d}{dt}\left(\ln\left(\frac{x_{root}M_{leaf,0}}{x_{leaf}M_{root,0}}\right) + v_{leaf,bio}\right)\right) + M_{seed,0} * e^{ln\left(\frac{x_{seed}M_{leaf,0}}{x_{leaf}M_{seed,0}}\right) + v_{leaf,bio}} * \left(ln\left(\frac{x_{seed}M_{leaf,0}}{x_{leaf}M_{seed,0}}\right) + v_{leaf,bio} + \frac{d}{dt}\left(ln\left(\frac{x_{seed}M_{leaf,0}}{x_{leaf}M_{seed,0}}\right) + v_{leaf,bio}\right)\right) + M_{stem,0} * e^{\ln\left(\frac{x_{stem}M_{leaf,0}}{x_{leaf}M_{stem,0}}\right) + v_{leaf,bio}} * \left(\ln\left(\frac{x_{stem}M_{leaf,0}}{x_{leaf}M_{stem,0}}\right) + v_{leaf,bio} + \frac{d}{dt}\left(\ln\left(\frac{x_{stem}M_{leaf,0}}{x_{leaf}M_{stem,0}}\right) + v_{leaf,bio}\right)\right)$$

Applying exponential rules and simplifying the above result gives:

$$\frac{dM_{plant}}{dt} = M_{leaf,0} * e^{v_{leaf,bio}} * \left(v_{leaf,bio} + \frac{d}{dt}\left(v_{leaf,bio}\right)\right) + M_{root,0} e^{v_{leaf,bio}} e^{\ln\left(\frac{x_{root} M_{leaf,0}}{x_{leaf} M_{root,0}}\right)}$$
$$* \left(\ln\left(\frac{x_{root} M_{leaf,0}}{x_{leaf} M_{root,0}}\right) + v_{leaf,bio} + \frac{d}{dt}\left(\ln\left(\frac{x_{root} M_{leaf,0}}{x_{leaf} M_{root,0}}\right) + v_{leaf,bio}\right)\right) + M_{seed,0}$$
$$* e^{v_{leaf,bio}} e^{ln\left(\frac{x_{seed} M_{leaf,0}}{x_{leaf} M_{seed,0}}\right)}$$
$$* \left(ln\left(\frac{x_{seed} M_{leaf,0}}{x_{leaf} M_{seed,0}}\right) + v_{leaf,bio} + \frac{d}{dt}\left(ln\left(\frac{x_{seed} M_{leaf,0}}{x_{leaf} M_{seed,0}}\right) + v_{leaf,bio}\right)\right) + M_{stem,0}$$
$$* e^{v_{leaf,bio}} e^{\ln\left(\frac{x_{stem} M_{leaf,0}}{x_{leaf} M_{stem,0}}\right)}$$
$$* \left(\ln\left(\frac{x_{stem} M_{leaf,0}}{x_{leaf} M_{stem,0}}\right) + v_{leaf,bio} + \frac{d}{dt}\left(\ln\left(\frac{x_{stem} M_{leaf,0}}{x_{leaf} M_{stem,0}}\right) + v_{leaf,bio}\right)\right)$$

$$\frac{dM_{plant}}{dt} = M_{leaf,0} * e^{v_{leaf,bio}} * \left(v_{leaf,bio} + \frac{d}{dt}\left(v_{leaf,bio}\right)\right)$$
$$+ M_{root,0} \frac{x_{root} M_{leaf,0}}{x_{leaf} M_{root,0}} e^{v_{leaf,bio}} \left(\ln\left(\frac{x_{root} M_{leaf,0}}{x_{leaf} M_{root,0}}\right) + v_{leaf,bio}\right.$$
$$\left. + \frac{d}{dt}\left(\ln\left(\frac{x_{root} M_{leaf,0}}{x_{leaf} M_{root,0}}\right) + v_{leaf,bio}\right)\right) + M_{seed,0} \frac{x_{seed} M_{leaf,0}}{x_{leaf} M_{seed,0}}$$
$$* e^{v_{leaf,bio}} \left(ln\left(\frac{x_{seed} M_{leaf,0}}{x_{leaf} M_{seed,0}}\right) + v_{leaf,bio} + \frac{d}{dt}\left(ln\left(\frac{x_{seed} M_{leaf,0}}{x_{leaf} M_{seed,0}}\right) + v_{leaf,bio}\right)\right)$$
$$+ M_{stem,0} \frac{x_{stem} M_{leaf,0}}{x_{leaf} M_{stem,0}}$$
$$* e^{v_{leaf,bio}} \left(\ln\left(\frac{x_{stem} M_{leaf,0}}{x_{leaf} M_{stem,0}}\right) + v_{leaf,bio} + \frac{d}{dt}\left(\ln\left(\frac{x_{stem} M_{leaf,0}}{x_{leaf} M_{stem,0}}\right) + v_{leaf,bio}\right)\right)$$

$$\frac{dM_{plant}}{dt} = e^{v_{leaf,bio}} \left[M_{leaf,0}\left(v_{leaf,bio} + \frac{d}{dt}\left(v_{leaf,bio}\right)\right)\right.$$
$$+ \frac{x_{root} M_{leaf,0}}{x_{leaf}}\left(\ln\left(\frac{x_{root} M_{leaf,0}}{x_{leaf} M_{root,0}}\right) + v_{leaf,bio} + \frac{d}{dt}\left(\ln\left(\frac{x_{root} M_{leaf,0}}{x_{leaf} M_{root,0}}\right) + v_{leaf,bio}\right)\right)$$
$$+ \frac{x_{seed} M_{leaf,0}}{x_{leaf}}\left(ln\left(\frac{x_{seed} M_{leaf,0}}{x_{leaf} M_{seed,0}}\right) + v_{leaf,bio} + \frac{d}{dt}\left(ln\left(\frac{x_{seed} M_{leaf,0}}{x_{leaf} M_{seed,0}}\right) + v_{leaf,bio}\right)\right)$$
$$\left. + \frac{x_{stem} M_{leaf,0}}{x_{leaf}}\left(\ln\left(\frac{x_{stem} M_{leaf,0}}{x_{leaf} M_{stem,0}}\right) + v_{leaf,bio} + \frac{d}{dt}\left(\ln\left(\frac{x_{stem} M_{leaf,0}}{x_{leaf} M_{stem,0}}\right) + v_{leaf,bio}\right)\right)\right]$$

Here it might be best to define some parameters used above. The term x_T represents the mass ratio of of tissues T to overall plant mass for the next time step, in other words:

$$x_{leaf} = \frac{M_{leaf}}{M_{plant}}$$

$$x_{root} = \frac{M_{root}}{M_{plant}}$$
$$x_{seed} = \frac{M_{seed}}{M_{plant}}$$
$$x_{stem} = \frac{M_{stem}}{M_{plant}}$$

Continuing:

$$\frac{dM_{plant}}{dt} = e^{v_{leaf,bio}} M_{leaf,0} \left[\left(v_{leaf,bio} + \frac{d}{dt}(v_{leaf,bio}) \right) + \frac{x_{root}}{x_{leaf}} \left(\ln\left(\frac{x_{root} M_{leaf,0}}{x_{leaf} M_{root,0}} \right) + v_{leaf,bio} + \frac{d}{dt}\left(\ln\left(\frac{x_{root} M_{leaf,0}}{x_{leaf} M_{root,0}} \right) + v_{leaf,bio} \right) \right) + \frac{x_{seed}}{x_{leaf}} \left(ln\left(\frac{x_{seed} M_{leaf,0}}{x_{leaf} M_{seed,0}} \right) + v_{leaf,bio} + \frac{d}{dt}\left(ln\left(\frac{x_{seed} M_{leaf,0}}{x_{leaf} M_{seed,0}} \right) + v_{leaf,bio} \right) \right) + \frac{x_{stem}}{x_{leaf}} \left(\ln\left(\frac{x_{stem} M_{leaf,0}}{x_{leaf} M_{stem,0}} \right) + v_{leaf,bio} + \frac{d}{dt}\left(\ln\left(\frac{x_{stem} M_{leaf,0}}{x_{leaf} M_{stem,0}} \right) + v_{leaf,bio} \right) \right) \right]$$

$$\frac{dM_{plant}}{dt} = e^{v_{leaf,bio}} M_{leaf,0} \left[\left(v_{leaf,bio} + \frac{d}{dt}(v_{leaf,bio}) \right) + \left(\frac{x_{root}}{x_{leaf}} \ln\left(\frac{x_{root} M_{leaf,0}}{x_{leaf} M_{root,0}} \right) + \frac{x_{root}}{x_{leaf}} v_{leaf,bio} + \frac{x_{root}}{x_{leaf}} \frac{d}{dt}\left(\ln\left(\frac{x_{root} M_{leaf,0}}{x_{leaf} M_{root,0}} \right) + v_{leaf,bio} \right) \right) + \left(\frac{x_{seed}}{x_{leaf}} ln\left(\frac{x_{seed} M_{leaf,0}}{x_{leaf} M_{seed,0}} \right) + \frac{x_{seed}}{x_{leaf}} v_{leaf,bio} + \frac{x_{seed}}{x_{leaf}} \frac{d}{dt}\left(ln\left(\frac{x_{seed} M_{leaf,0}}{x_{leaf} M_{seed,0}} \right) + v_{leaf,bio} \right) \right) + \left(\frac{x_{stem}}{x_{leaf}} \ln\left(\frac{x_{stem} M_{leaf,0}}{x_{leaf} M_{stem,0}} \right) + \frac{x_{stem}}{x_{leaf}} v_{leaf,bio} + \frac{x_{stem}}{x_{leaf}} \frac{d}{dt}\left(\ln\left(\frac{x_{stem} M_{leaf,0}}{x_{leaf} M_{stem,0}} \right) + v_{leaf,bio} \right) \right) \right]$$

$$\frac{dM_{plant}}{dt} = e^{v_{leaf,bio}} M_{leaf,0} \left[v_{leaf,bio} + \frac{d}{dt}(v_{leaf,bio}) + \frac{x_{root}}{x_{leaf}} \ln\left(\frac{x_{root} M_{leaf,0}}{x_{leaf} M_{root,0}} \right) + \frac{x_{root}}{x_{leaf}} v_{leaf,bio} + \frac{x_{root}}{x_{leaf}} \frac{d}{dt}\left(\ln\left(\frac{x_{root} M_{leaf,0}}{x_{leaf} M_{root,0}} \right) + v_{leaf,bio} \right) + \frac{x_{seed}}{x_{leaf}} ln\left(\frac{x_{seed} M_{leaf,0}}{x_{leaf} M_{seed,0}} \right) + \frac{x_{seed}}{x_{leaf}} v_{leaf,bio} + \frac{x_{seed}}{x_{leaf}} \frac{d}{dt}\left(ln\left(\frac{x_{seed} M_{leaf,0}}{x_{leaf} M_{seed,0}} \right) + v_{leaf,bio} \right) + \frac{x_{stem}}{x_{leaf}} \ln\left(\frac{x_{stem} M_{leaf,0}}{x_{leaf} M_{stem,0}} \right) + \frac{x_{stem}}{x_{leaf}} v_{leaf,bio} + \frac{x_{stem}}{x_{leaf}} \frac{d}{dt}\left(\ln\left(\frac{x_{stem} M_{leaf,0}}{x_{leaf} M_{stem,0}} \right) + v_{leaf,bio} \right) \right]$$

$$\frac{dM_{plant}}{dt} = e^{v_{leaf,bio}} M_{leaf,0} \left[v_{leaf,bio} + \frac{x_{root}}{x_{leaf}} \ln\left(\frac{x_{root} M_{leaf,0}}{x_{leaf} M_{root,0}}\right) + \frac{x_{root}}{x_{leaf}} v_{leaf,bio} \right.$$
$$+ \frac{x_{seed}}{x_{leaf}} ln\left(\frac{x_{seed} M_{leaf,0}}{x_{leaf} M_{seed,0}}\right) + \frac{x_{seed}}{x_{leaf}} v_{leaf,bio} + \frac{x_{stem}}{x_{leaf}} \ln\left(\frac{x_{stem} M_{leaf,0}}{x_{leaf} M_{stem,0}}\right) + \frac{x_{stem}}{x_{leaf}} v_{leaf,bio}$$
$$+ \frac{d}{dt}\left(v_{leaf,bio}\right) + \frac{x_{root}}{x_{leaf}} \frac{d}{dt}\left(\ln\left(\frac{x_{root} M_{leaf,0}}{x_{leaf} M_{root,0}}\right) + v_{leaf,bio}\right)$$
$$+ \frac{x_{seed}}{x_{leaf}} \frac{d}{dt}\left(ln\left(\frac{x_{seed} M_{leaf,0}}{x_{leaf} M_{seed,0}}\right) + v_{leaf,bio}\right)$$
$$\left. + \frac{x_{stem}}{x_{leaf}} \frac{d}{dt}\left(\ln\left(\frac{x_{stem} M_{leaf,0}}{x_{leaf} M_{stem,0}}\right) + v_{leaf,bio}\right) \right]$$

Combine derivative terms for the sake of combining like terms:

$$\frac{dM_{plant}}{dt} = e^{v_{leaf,bio}} M_{leaf,0} \left[v_{leaf,bio} + \frac{x_{root}}{x_{leaf}} \ln\left(\frac{x_{root} M_{leaf,0}}{x_{leaf} M_{root,0}}\right) + \frac{x_{root}}{x_{leaf}} v_{leaf,bio} \right.$$
$$+ \frac{x_{seed}}{x_{leaf}} ln\left(\frac{x_{seed} M_{leaf,0}}{x_{leaf} M_{seed,0}}\right) + \frac{x_{seed}}{x_{leaf}} v_{leaf,bio} + \frac{x_{stem}}{x_{leaf}} \ln\left(\frac{x_{stem} M_{leaf,0}}{x_{leaf} M_{stem,0}}\right) + \frac{x_{stem}}{x_{leaf}} v_{leaf,bio}$$
$$+ \frac{d}{dt}\left(v_{leaf,bio} + \frac{x_{root}}{x_{leaf}} \ln\left(\frac{x_{root} M_{leaf,0}}{x_{leaf} M_{root,0}}\right) + \frac{x_{root}}{x_{leaf}} v_{leaf,bio} + \frac{x_{seed}}{x_{leaf}} ln\left(\frac{x_{seed} M_{leaf,0}}{x_{leaf} M_{seed,0}}\right) \right.$$
$$\left.\left. + \frac{x_{seed}}{x_{leaf}} v_{leaf,bio} + \frac{x_{stem}}{x_{leaf}} \ln\left(\frac{x_{stem} M_{leaf,0}}{x_{leaf} M_{stem,0}}\right) + \frac{x_{stem}}{x_{leaf}} v_{leaf,bio} \right) \right]$$

For the sake of compactness, let us make the following substitutions:

$$\boldsymbol{\mu(t) = \frac{x_{root} M_{leaf,0}}{x_{leaf} M_{root,0}}}$$
$$\boldsymbol{\theta(t) = \frac{x_{seed} M_{leaf,0}}{x_{leaf} M_{seed,0}}}$$
$$\boldsymbol{\lambda(t) = \frac{x_{stem} M_{leaf,0}}{x_{leaf} M_{stem,0}}}$$

Making the above equation:

$$\frac{dM_{plant}}{dt} = e^{v_{leaf,bio}} M_{leaf,0} \left[v_{leaf,bio} + \frac{x_{root}}{x_{leaf}} \ln(\mu(t)) + \frac{x_{root}}{x_{leaf}} v_{leaf,bio} + \frac{x_{seed}}{x_{leaf}} ln(\theta(t)) \right.$$
$$+ \frac{x_{seed}}{x_{leaf}} v_{leaf,bio} + \frac{x_{stem}}{x_{leaf}} \ln(\lambda(t)) + \frac{x_{stem}}{x_{leaf}} v_{leaf,bio}$$
$$+ \frac{d}{dt}\left(v_{leaf,bio} + \frac{x_{root}}{x_{leaf}} \ln(\mu(t)) + \frac{x_{root}}{x_{leaf}} v_{leaf,bio} + \frac{x_{seed}}{x_{leaf}} ln(\theta(t)) + \frac{x_{seed}}{x_{leaf}} v_{leaf,bio} \right.$$
$$\left.\left. + \frac{x_{stem}}{x_{leaf}} \ln(\lambda(t)) + \frac{x_{stem}}{x_{leaf}} v_{leaf,bio} \right) \right]$$

For the sake of clarity, let us consider only the derivative term:

$$\frac{d}{dt}\left(v_{leaf,bio}+\frac{x_{root}}{x_{leaf}}\ln(\mu(t))+\frac{x_{root}}{x_{leaf}}v_{leaf,bio}+\frac{x_{seed}}{x_{leaf}}ln(\theta(t))+\frac{x_{seed}}{x_{leaf}}v_{leaf,bio}+\frac{x_{stem}}{x_{leaf}}\ln(\lambda(t))+\frac{x_{stem}}{x_{leaf}}v_{leaf,bio}\right)$$

Distributing the derivative:

$$\frac{d}{dt}\left(v_{leaf,bio}\right)+\frac{d}{dt}\left(\frac{x_{root}}{x_{leaf}}\ln(\mu(t))\right)+\frac{d}{dt}\left(\frac{x_{root}}{x_{leaf}}v_{leaf,bio}\right)+\frac{d}{dt}\left(\frac{x_{seed}}{x_{leaf}}ln(\theta(t))\right)+\frac{d}{dt}\left(\frac{x_{seed}}{x_{leaf}}v_{leaf,bio}\right)+\frac{d}{dt}\left(\frac{x_{stem}}{x_{leaf}}\ln(\lambda(t))\right)+\frac{d}{dt}\left(\frac{x_{stem}}{x_{leaf}}v_{leaf,bio}\right)$$

Using the product rule:

$$\frac{d}{dt}\left(v_{leaf,bio}\right)+\frac{x_{root}}{x_{leaf}}\frac{d}{dt}(\ln(\mu(t)))+\ln(\mu(t))\frac{d}{dt}\left(\frac{x_{root}}{x_{leaf}}\right)+\frac{x_{root}}{x_{leaf}}\frac{d}{dt}\left(v_{leaf,bio}\right)+v_{leaf,bio}\frac{d}{dt}\left(\frac{x_{root}}{x_{leaf}}\right)+\frac{x_{seed}}{x_{leaf}}\frac{d}{dt}\left(ln(\theta(t))\right)+ln(\theta(t))\frac{d}{dt}\left(\frac{x_{seed}}{x_{leaf}}\right)+\frac{x_{seed}}{x_{leaf}}\frac{d}{dt}\left(v_{leaf,bio}\right)+v_{leaf,bio}\frac{d}{dt}\left(\frac{x_{seed}}{x_{leaf}}\right)+\frac{x_{stem}}{x_{leaf}}\frac{d}{dt}(\ln(\lambda(t)))+\ln(\lambda(t))\frac{d}{dt}\left(\frac{x_{stem}}{x_{leaf}}\right)+\frac{x_{stem}}{x_{leaf}}\frac{d}{dt}\left(v_{leaf,bio}\right)+v_{leaf,bio}\frac{d}{dt}\left(\frac{x_{stem}}{x_{leaf}}\right)$$

Combining like terms:

$$\frac{x_{leaf}}{x_{leaf}}\frac{d}{dt}\left(v_{leaf,bio}\right)+\frac{x_{root}}{x_{leaf}}\frac{d}{dt}\left(v_{leaf,bio}\right)+\frac{x_{seed}}{x_{leaf}}\frac{d}{dt}\left(v_{leaf,bio}\right)+\frac{x_{stem}}{x_{leaf}}\frac{d}{dt}\left(v_{leaf,bio}\right)+\ln(\mu(t))\frac{d}{dt}\left(\frac{x_{root}}{x_{leaf}}\right)+v_{leaf,bio}\frac{d}{dt}\left(\frac{x_{root}}{x_{leaf}}\right)+ln(\theta(t))\frac{d}{dt}\left(\frac{x_{seed}}{x_{leaf}}\right)+v_{leaf,bio}\frac{d}{dt}\left(\frac{x_{seed}}{x_{leaf}}\right)+\ln(\lambda(t))\frac{d}{dt}\left(\frac{x_{stem}}{x_{leaf}}\right)+v_{leaf,bio}\frac{d}{dt}\left(\frac{x_{stem}}{x_{leaf}}\right)+\frac{x_{root}}{x_{leaf}}\frac{d}{dt}(\ln(\mu(t)))+\frac{x_{seed}}{x_{leaf}}\frac{d}{dt}\left(ln(\theta(t))\right)+\frac{x_{stem}}{x_{leaf}}\frac{d}{dt}(\ln(\lambda(t)))$$

$$\left(\frac{x_{leaf}}{x_{leaf}}+\frac{x_{root}}{x_{leaf}}+\frac{x_{seed}}{x_{leaf}}+\frac{x_{stem}}{x_{leaf}}\right)\frac{d}{dt}\left(v_{leaf,bio}\right)+\left(\ln(\mu(t))+v_{leaf,bio}\right)\frac{d}{dt}\left(\frac{x_{root}}{x_{leaf}}\right)+l\left(n(\theta(t))+v_{leaf,bio}\right)\frac{d}{dt}\left(\frac{x_{seed}}{x_{leaf}}\right)+\left(\ln(\lambda(t))+v_{leaf,bio}\right)\frac{d}{dt}\left(\frac{x_{stem}}{x_{leaf}}\right)+\frac{1}{x_{leaf}}\left(x_{root}\frac{d}{dt}(\ln(\mu(t)))+x_{seed}\frac{d}{dt}\left(ln(\theta(t))\right)+x_{stem}\frac{d}{dt}(\ln(\lambda(t)))\right)$$

$$\frac{1}{x_{leaf}}\left(x_{leaf}+x_{root}+x_{seed}+x_{stem}\right)\frac{d}{dt}\left(v_{leaf,bio}\right)+\left(\ln(\mu(t))+v_{leaf,bio}\right)\frac{d}{dt}\left(\frac{x_{root}}{x_{leaf}}\right)$$
$$+\left(ln(\theta(t))+v_{leaf,bio}\right)\frac{d}{dt}\left(\frac{x_{seed}}{x_{leaf}}\right)+\left(\ln(\lambda(t))+v_{leaf,bio}\right)\frac{d}{dt}\left(\frac{x_{stem}}{x_{leaf}}\right)$$
$$+\frac{1}{x_{leaf}}\left(x_{root}\frac{d}{dt}(\ln(\mu(t)))+x_{seed}\frac{d}{dt}\left(ln(\theta(t))\right)+x_{stem}\frac{d}{dt}(\ln(\lambda(t)))\right)$$

Since there are only four tissues and x_T is the mass fraction of that tissue in the total plant:

$$x_{leaf}+x_{root}+x_{seed}+x_{stem}=1$$

Therefore:

$$\frac{1}{x_{leaf}}\frac{d}{dt}\left(v_{leaf,bio}\right)+\left(\ln(\mu(t))+v_{leaf,bio}\right)\frac{d}{dt}\left(\frac{x_{root}}{x_{leaf}}\right)+\left(ln(\theta(t))+v_{leaf,bio}\right)\frac{d}{dt}\left(\frac{x_{seed}}{x_{leaf}}\right)$$
$$+\left(\ln(\lambda(t))+v_{leaf,bio}\right)\frac{d}{dt}\left(\frac{x_{stem}}{x_{leaf}}\right)$$
$$+\frac{1}{x_{leaf}}\left(x_{root}\frac{d}{dt}(\ln(\mu(t)))+x_{seed}\frac{d}{dt}\left(ln(\theta(t))\right)+x_{stem}\frac{d}{dt}(\ln(\lambda(t)))\right)$$

Further, each tissues mass fraction is a linear function of some overarching variable s, representing the fraction of seeds present in the plant mass compared to the maximum fraction of seed mass. This variable is generally called “seeding”. These relations are:

$$x_{leaf}=c_{leaf}s+x_{leaf,0}$$
$$x_{root}=c_{root}s+x_{root,0}$$
$$x_{seed}=c_{seed}s$$
$$x_{stem}=c_{stem}s+x_{stem,0}$$

Making the appropriate substitutions:

$$\frac{1}{x_{leaf}}\frac{d}{dt}\left(v_{leaf,bio}\right)+\left(\ln(\mu(t))+v_{leaf,bio}\right)\frac{d}{dt}\left(\frac{c_{root}s+x_{root,0}}{c_{leaf}s+x_{leaf,0}}\right)$$
$$+\left(ln(\theta(t))+v_{leaf,bio}\right)\frac{d}{dt}\left(\frac{c_{seed}s}{c_{leaf}s+x_{leaf,0}}\right)$$
$$+\left(\ln(\lambda(t))+v_{leaf,bio}\right)\frac{d}{dt}\left(\frac{c_{stem}s+x_{stem,0}}{c_{leaf}s+x_{leaf,0}}\right)$$
$$+\frac{1}{x_{leaf}}\left(x_{root}\frac{d}{dt}(\ln(\mu(t)))+x_{seed}\frac{d}{dt}\left(ln(\theta(t))\right)+x_{stem}\frac{d}{dt}(\ln(\lambda(t)))\right)$$

For now, consider only the derivative terms with variable s in them to avoid excessive repetitive writing. Let us look at the first such term:

$$\frac{d}{dt}\left(\frac{c_{root}s + x_{root,0}}{c_{leaf}s + x_{leaf,0}}\right)$$

Applying the quotient rule:

$$\frac{d}{dt}\left(\frac{c_{root}s + x_{root,0}}{c_{leaf}s + x_{leaf,0}}\right) = \frac{\left(c_{leaf}s + x_{leaf,0}\right)\frac{d}{dt}\left(c_{root}s + x_{root,0}\right) - \left(c_{root}s + x_{root,0}\right)\frac{d}{dt}\left(c_{leaf}s + x_{leaf,0}\right)}{\left(c_{leaf}s + x_{leaf,0}\right)^2}$$

Simplifying by recognizing $x_{root,0}$, $x_{leaf,0}$, c_{root}, c_{leaf} are constants:

$$\frac{d}{dt}\left(\frac{c_{root}s + x_{root,0}}{c_{leaf}s + x_{leaf,0}}\right) = \frac{c_{root}\left(c_{leaf}s + x_{leaf,0}\right)\frac{d}{dt}(s) - c_{leaf}\left(c_{root}s + x_{root,0}\right)\frac{d}{dt}(s)}{c_{leaf}^2 s^2 + 2c_{leaf}s x_{leaf,0} + x_{leaf,0}^2}$$

Combining like terms:

$$\frac{d}{dt}\left(\frac{c_{root}s + x_{root,0}}{c_{leaf}s + x_{leaf,0}}\right) = \frac{c_{root}\left(c_{leaf}s + x_{leaf,0}\right) - c_{leaf}\left(c_{root}s + x_{root,0}\right)}{c_{leaf}^2 s^2 + 2c_{leaf}s x_{leaf,0} + x_{leaf,0}^2}\frac{d}{dt}(s)$$

For compactness, let us define:

$$\boldsymbol{\zeta = \frac{c_{root}\left(c_{leaf}s + x_{leaf,0}\right) - c_{leaf}\left(c_{root}s + x_{root,0}\right)}{c_{leaf}^2 s^2 + 2c_{leaf}s x_{leaf,0} + x_{leaf,0}^2}}$$

Therefore:

$$\frac{d}{dt}\left(\frac{c_{root}s + x_{root,0}}{c_{leaf}s + x_{leaf,0}}\right) = \zeta\frac{d}{dt}(s)$$

Similarly:

$$\frac{d}{dt}\left(\frac{c_{seed}s}{c_{leaf}s + x_{leaf,0}}\right) = \frac{\left(c_{leaf}s + x_{leaf,0}\right)\frac{d}{dt}(c_{seed}s) - (c_{seed}s)\frac{d}{dt}\left(c_{leaf}s + x_{leaf,0}\right)}{\left(c_{leaf}s + x_{leaf,0}\right)^2}$$

$$\frac{d}{dt}\left(\frac{c_{seed}s}{c_{leaf}s + x_{leaf,0}}\right) = \frac{c_{seed}\left(c_{leaf}s + x_{leaf,0}\right)\frac{d}{dt}(s) - c_{leaf}(c_{seed}s)\frac{d}{dt}(s)}{c_{leaf}^2 s^2 + 2c_{leaf}s x_{leaf,0} + x_{leaf,0}^2}$$

$$\frac{d}{dt}\left(\frac{c_{seed}s}{c_{leaf}s + x_{leaf,0}}\right) = \frac{c_{seed}\left(c_{leaf}s + x_{leaf,0}\right) + c_{leaf}(c_{seed}s)}{c_{leaf}^2 s^2 + 2c_{leaf}s x_{leaf,0} + x_{leaf,0}^2}\frac{d}{dt}(s)$$

Let:

$$\boldsymbol{\rho = \frac{c_{seed}(c_{leaf}s + x_{leaf,0}) - c_{leaf}(c_{seed}s)}{c_{leaf}^2 s^2 + 2c_{leaf}s x_{leaf,0} + x_{leaf,0}^2}}$$

Then:

$$\frac{d}{dt}\left(\frac{c_{seed}s}{c_{leaf}s + x_{leaf,0}}\right) = \rho \frac{d}{dt}(s)$$

And finally:

$$\frac{d}{dt}\left(\frac{c_{stem}s + x_{stem,0}}{c_{leaf}s + x_{leaf,0}}\right)$$
$$= \frac{(c_{leaf}s + x_{leaf,0})\frac{d}{dt}(c_{stem}s + x_{stem,0}) - (c_{stem}s + x_{stem,0})\frac{d}{dt}(c_{leaf}s + x_{leaf,0})}{(c_{leaf}s + x_{leaf,0})^2}$$
$$\frac{d}{dt}\left(\frac{c_{stem}s + x_{stem,0}}{c_{leaf}s + x_{leaf,0}}\right) = \frac{c_{stem}(c_{leaf}s + x_{leaf,0})\frac{d}{dt}(s) - c_{leaf}(c_{stem}s + x_{stem,0})\frac{d}{dt}(s)}{c_{leaf}^2 s^2 + 2c_{leaf}s x_{leaf,0} + x_{leaf,0}^2}$$
$$\frac{d}{dt}\left(\frac{c_{stem}s + x_{stem,0}}{c_{leaf}s + x_{leaf,0}}\right) = \frac{c_{stem}(c_{leaf}s + x_{leaf,0}) - c_{leaf}(c_{stem}s + x_{stem,0})}{c_{leaf}^2 s^2 + 2c_{leaf}s x_{leaf,0} + x_{leaf,0}^2}\frac{d}{dt}(s)$$

Let:

$$\boldsymbol{\iota = \frac{c_{stem}(c_{leaf}s + x_{leaf,0}) - c_{leaf}(c_{stem}s + x_{stem,0})}{c_{leaf}^2 s^2 + 2c_{leaf}s x_{leaf,0} + x_{leaf,0}^2}}$$

Then:

$$\frac{d}{dt}\left(\frac{c_{stem}s + x_{stem,0}}{c_{leaf}s + x_{leaf,0}}\right) = \omega \frac{d}{dt}(s)$$

Making the appropriate substitutions:

$$\frac{1}{x_{leaf}}\frac{d}{dt}(v_{leaf,bio}) + (\ln(\mu(t)) + v_{leaf,bio})\zeta\frac{d}{dt}(s) + (ln(\theta(t)) + v_{leaf,bio})\rho\frac{d}{dt}(s)$$
$$+ (\ln(\lambda(t)) + v_{leaf,bio})\omega\frac{d}{dt}(s)$$
$$+ \frac{1}{x_{leaf}}\left(x_{root}\frac{d}{dt}(\ln(\mu(t))) + x_{seed}\frac{d}{dt}(ln(\theta(t))) + x_{stem}\frac{d}{dt}(\ln(\lambda(t)))\right)$$

Combining like terms:

$$\frac{1}{x_{leaf}}\frac{d}{dt}\left(v_{leaf,bio}\right)$$
$$+\left(\left(\ln(\mu(t))+v_{leaf,bio}\right)\zeta+\left(ln(\theta(t))+v_{leaf,bio}\right)\rho+\left(\ln(\lambda(t))+v_{leaf,bio}\right)\omega\right)\frac{d}{dt}(s)$$
$$+\frac{1}{x_{leaf}}\left(x_{root}\frac{d}{dt}(\ln(\mu(t)))+x_{seed}\frac{d}{dt}\left(ln(\theta(t))\right)+x_{stem}\frac{d}{dt}(\ln(\lambda(t)))\right)$$

Substituting the derivative term back into the overall equation gives:

$$\frac{dM_{plant}}{dt}=e^{v_{leaf,bio}}M_{leaf,0}\left[v_{leaf,bio}+\frac{x_{root}}{x_{leaf}}\ln(\mu(t))+\frac{x_{root}}{x_{leaf}}v_{leaf,bio}+\frac{x_{seed}}{x_{leaf}}ln(\theta(t))\right.$$
$$+\frac{x_{seed}}{x_{leaf}}v_{leaf,bio}+\frac{x_{stem}}{x_{leaf}}\ln(\lambda(t))+\frac{x_{stem}}{x_{leaf}}v_{leaf,bio}+\frac{1}{x_{leaf}}\frac{d}{dt}\left(v_{leaf,bio}\right)$$
$$+\left(\left(\ln(\mu(t))+v_{leaf,bio}\right)\zeta+\left(ln(\theta(t))+v_{leaf,bio}\right)\rho+\left(\ln(\lambda(t))+v_{leaf,bio}\right)\omega\right)\frac{d}{dt}(s)$$
$$\left.+\frac{1}{x_{leaf}}\left(x_{root}\frac{d}{dt}(\ln(\mu(t)))+x_{seed}\frac{d}{dt}\left(ln(\theta(t))\right)+x_{stem}\frac{d}{dt}(\ln(\lambda(t)))\right)\right]$$

$$\frac{dM_{plant}}{dt}=\frac{e^{v_{leaf,bio}}M_{leaf,0}}{x_{leaf}}\left[x_{leaf}v_{leaf,bio}+x_{root}\ln(\mu(t))+x_{root}v_{leaf,bio}+x_{seed}ln(\theta(t))\right.$$
$$+x_{seed}v_{leaf,bio}+x_{stem}\ln(\lambda(t))+x_{stem}v_{leaf,bio}+\frac{d}{dt}\left(v_{leaf,bio}\right)$$
$$+x_{leaf}\left(\left(\ln(\mu(t))+v_{leaf,bio}\right)\zeta+\left(ln(\theta(t))+v_{leaf,bio}\right)\rho\right.$$
$$\left.+\left(\ln(\lambda(t))+v_{leaf,bio}\right)\omega\right)\frac{d}{dt}(s)$$
$$\left.+\left(x_{root}\frac{d}{dt}(\ln(\mu(t)))+x_{seed}\frac{d}{dt}\left(ln(\theta(t))\right)+x_{stem}\frac{d}{dt}(\ln(\lambda(t)))\right)\right]$$

$$\frac{dM_{plant}}{dt}=\frac{e^{v_{leaf,bio}}M_{leaf,0}}{x_{leaf}}\left[x_{leaf}v_{leaf,bio}+x_{root}\left(\ln(\mu(t))+v_{leaf,bio}\right)+x_{seed}\left(ln(\theta(t))+v_{leaf,bio}\right)\right.$$
$$+x_{stem}\left(\ln(\lambda(t))+v_{leaf,bio}\right)+\frac{d}{dt}\left(v_{leaf,bio}\right)$$
$$+x_{leaf}\left(\left(\ln(\mu(t))+v_{leaf,bio}\right)\zeta+\left(ln(\theta(t))+v_{leaf,bio}\right)\rho\right.$$
$$\left.+\left(\ln(\lambda(t))+v_{leaf,bio}\right)\omega\right)\frac{d}{dt}(s)$$
$$\left.+\left(x_{root}\frac{d}{dt}(\ln(\mu(t)))+x_{seed}\frac{d}{dt}\left(ln(\theta(t))\right)+x_{stem}\frac{d}{dt}(\ln(\lambda(t)))\right)\right]$$

Let:

$$\boldsymbol{\psi(t)=\ln(\mu(t))+v_{leaf,bio}}$$
$$\boldsymbol{\pi(t)=\ln(\theta(t))+v_{leaf,bio}}$$
$$\boldsymbol{\kappa(t)=\ln(\lambda(t))+v_{leaf,bio}}$$

Making the substitutions:

$$\frac{dM_{plant}}{dt} = \frac{e^{v_{leaf,bio}} M_{leaf,0}}{x_{leaf}} \Big[x_{leaf} v_{leaf,bio} + x_{root}\psi(t) + x_{seed}\pi(t) + x_{stem}\kappa(t) + \frac{d}{dt}\left(v_{leaf,bio}\right) + x_{leaf}\left(\psi(t)\zeta(t) + \pi(t)\rho(t) + \kappa(t)\omega(t)\right)\frac{d}{dt}(s) + x_{root}\frac{d}{dt}(\ln(\mu(t))) + x_{seed}\frac{d}{dt}\left(ln(\theta(t))\right) + x_{stem}\frac{d}{dt}(\ln(\lambda(t))) \Big]$$

Making a final substitution separating growth rates from equations dependent on time points:

$$\frac{dM_{plant}}{dt} = \frac{e^{v_{leaf,bio}} M_{leaf,0}}{x_{leaf}} \left[x_{leaf} v_{leaf,bio} + \frac{d}{dt}\left(v_{leaf,bio}\right) + \xi \right]$$

$$\xi(t) = x_{root}\psi(t) + x_{seed}\pi(t) + x_{stem}\kappa(t) + x_{leaf}\left(\psi(t)\zeta(t) + \pi(t)\rho(t) + \kappa(t)\omega(t)\right)\frac{d}{dt}(s) + x_{root}\frac{d}{dt}(\ln(\mu(t))) + x_{seed}\frac{d}{dt}\left(ln(\theta(t))\right) + x_{stem}\frac{d}{dt}(\ln(\lambda(t)))$$

Therefore the derivative is:

$$\boldsymbol{\frac{dM_{plant}}{dt} = \frac{e^{v_{leaf,bio}} M_{leaf,0}}{x_{leaf}} \left[x_{leaf} v_{leaf,bio} + \frac{d}{dt}\left(v_{leaf,bio}\right) + \xi(t) \right]}$$

$$\boldsymbol{\xi(t) = x_{root}\psi(t) + x_{seed}\pi(t) + x_{stem}\kappa(t) + x_{leaf}\left(\psi(t)\zeta(t) + \pi(t)\rho(t) + \kappa(t)\iota(t)\right)\frac{d}{dt}(s) + x_{root}\frac{d}{dt}(\ln(\mu(t))) + x_{seed}\frac{d}{dt}\left(ln(\theta(t))\right) + x_{stem}\frac{d}{dt}(\ln(\lambda(t)))}$$

$$\boldsymbol{\psi(t) = \ln(\mu(t)) + v_{leaf,bio}}$$

$$\boldsymbol{\pi(t) = \ln(\theta(t)) + v_{leaf,bio}}$$

$$\boldsymbol{\kappa(t) = \ln(\lambda(t)) + v_{leaf,bio}}$$

$$\boldsymbol{\mu(t) = \frac{x_{root} M_{leaf,0}}{x_{leaf} M_{root,0}}}$$

$$\boldsymbol{\theta(t) = \frac{x_{seed} M_{leaf,0}}{x_{leaf} M_{seed,0}}}$$

$$\boldsymbol{\lambda(t) = \frac{x_{stem} M_{leaf,0}}{x_{leaf} M_{stem,0}}}$$

$$\boldsymbol{\zeta = \frac{c_{root}\left(c_{leaf}s + x_{leaf,0}\right) - c_{leaf}\left(c_{root}s + x_{root,0}\right)}{c_{leaf}^2 s^2 + 2c_{leaf} s x_{leaf,0} + x_{leaf,0}^2}}$$

$$\boldsymbol{\rho = \frac{c_{seed}\left(c_{leaf}s + x_{leaf,0}\right) - c_{leaf}\left(c_{seed}s\right)}{c_{leaf}^2 s^2 + 2c_{leaf} s x_{leaf,0} + x_{leaf,0}^2}}$$

$$\boldsymbol{\iota = \frac{c_{stem}\left(c_{leaf}s + x_{leaf,0}\right) - c_{leaf}\left(c_{stem}s + x_{stem,0}\right)}{c_{leaf}^2 s^2 + 2c_{leaf} s x_{leaf,0} + x_{leaf,0}^2}}$$

All equations not dependent on growth rate will be treated as parameters at that time point, whereas those that are dependent on the biomass rate will be treated as variables. Therefore:

Parameters	Variables
$\boldsymbol{x_{leaf}, x_{root}, x_{seed}, x_{stem}}$<br>$\boldsymbol{\mu(t), \theta(t), \lambda(t)}$<br>$\boldsymbol{\zeta, \rho, \omega}$	$\boldsymbol{v_{leaf,bio}}$<br>$\boldsymbol{\frac{d}{dt}(v_{leaf,bio})}$<br>$\boldsymbol{\frac{dM_{plant}}{dt}}$<br>$\boldsymbol{\psi(t), \pi(t), \kappa(t), \xi(t)}$
